# Alkyne Derivatives of SARS-CoV-2 Main Protease Inhibitors Including Nirmatrelvir Inhibit by Reacting Covalently with the Nucleophilic Cysteine

**DOI:** 10.1021/acs.jmedchem.2c01627

**Published:** 2023-02-09

**Authors:** Lennart Brewitz, Leo Dumjahn, Yilin Zhao, C. David Owen, Stephen M. Laidlaw, Tika R. Malla, Dung Nguyen, Petra Lukacik, Eidarus Salah, Adam D. Crawshaw, Anna J. Warren, Jose Trincao, Claire Strain-Damerell, Miles W. Carroll, Martin A. Walsh, Christopher J. Schofield

**Affiliations:** † Chemistry Research Laboratory, Department of Chemistry and the Ineos Oxford Institute for Antimicrobial Research, University of Oxford, 12 Mansfield Road, Oxford OX1 3TA, United Kingdom; ‡ Diamond Light Source Ltd., Harwell Science and Innovation Campus, Didcot OX11 0DE, United Kingdom; § Research Complex at Harwell, Harwell Science and Innovation Campus, Didcot OX11 0FA, United Kingdom; ∥ Wellcome Centre for Human Genetics, Nuffield Department of Medicine, University of Oxford, Oxford OX3 7BN, United Kingdom

## Abstract

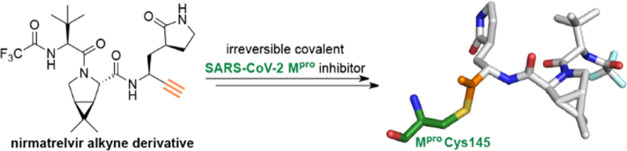

Nirmatrelvir (PF-07321332) is a nitrile-bearing small-molecule inhibitor that, in combination with ritonavir, is used to treat infections by severe acute respiratory syndrome coronavirus-2 (SARS-CoV-2). Nirmatrelvir interrupts the viral life cycle by inhibiting the SARS-CoV-2 main protease (M^pro^), which is essential for processing viral polyproteins into functional nonstructural proteins. We report studies which reveal that derivatives of nirmatrelvir and other M^pro^ inhibitors with a nonactivated terminal alkyne group positioned similarly to the electrophilic nitrile of nirmatrelvir can efficiently inhibit isolated M^pro^ and SARS-CoV-2 replication in cells. Mass spectrometric and crystallographic evidence shows that the alkyne derivatives inhibit M^pro^ by apparent irreversible covalent reactions with the active site cysteine (Cys145), while the analogous nitriles react reversibly. The results highlight the potential for irreversible covalent inhibition of M^pro^ and other nucleophilic cysteine proteases by alkynes, which, in contrast to nitriles, can be functionalized at their terminal position to optimize inhibition and selectivity, as well as pharmacodynamic and pharmacokinetic properties.

## Introduction

**Figure 1:**
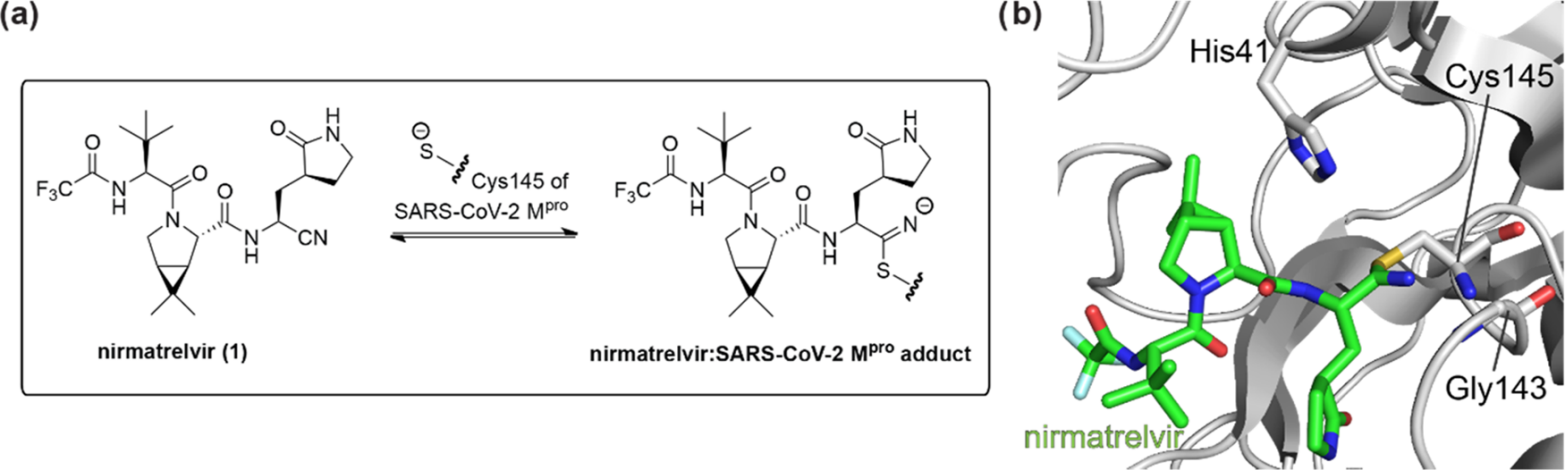
Nirmatrelvir inhibits SARS-CoV-2 M^pro^ by reversible covalent reaction with the nucleophilic Cys145. (a) Reversible covalent reaction of nirmatrelvir (PF-07321332, **1**) with the nucleophilic thiolate of SARS-CoV-2 M^pro^ Cys145 (deprotonated by His41).^1^ (b) View from a crystal structure of SARS-CoV-2 M^pro^ (gray cartoon) in complex with nirmatrelvir (**1**; green carbon backbone) (PDB ID: 7TE0(22)). The thioimidate is located in an oxyanion hole (involving the main chain amide NHs of Cys145 and Gly143).

In late 2021, the small-molecule active pharmaceutical ingredient (API) of paxlovid, i.e., nirmatrelvir (PF-07321332, **1**; Figure 1a),^1^ was approved for emergency use in humans to treat COVID-19 (in combination with ritonavir). Nirmatrelvir is a selective inhibitor of the SARS-CoV-2^2^ main protease (M^pro^ or 3C-like protease, 3CL^pro^).^1^ M^pro^ is a nucleophilic cysteine protease that, together with the papain-like cysteine protease (PL^pro^), catalyzes hydrolysis of viral polyproteins to give functional nonstructural proteins (nsps), a process essential for the viral life cycle, rendering M^pro^ and PL^pro^ attractive drug targets.^3−8^ The therapeutic use of nirmatrelvir is precedented by many small-molecule viral protease inhibitors that are used to treat infections by human immunodeficiency virus (HIV) and hepatitis C virus (HCV).^9^

SARS-CoV-2 M^pro^ may be a more viable medicinal chemistry target than PL^pro^ because its fold and substrate selectivities are reported to be different from human proteases;^10,11^ M^pro^ is highly conserved among coronaviruses, including SARS-CoV-2 variants of clinical concern.^12^ Indeed, nirmatrelvir inhibits SARS-CoV-2 variants, including omicron^13−17^ and other coronaviruses,^1,18−20^ but does not substantially inhibit most tested human cysteine proteases in vitro.^1,21^

**Figure 2:**
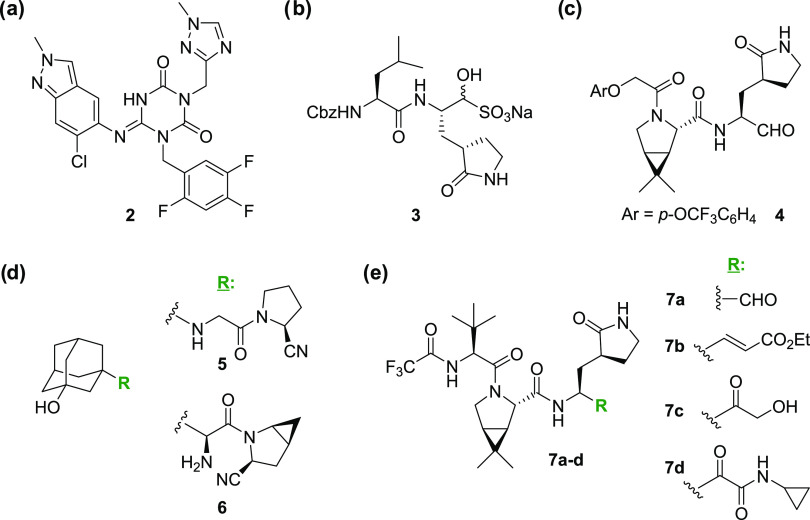
SARS-CoV-2 M^pro^ and DPP4 inhibitors. (a) Ensitrelvir (S-217622, **2**),^23^ (b) GC376 (**3**),^25,26,31^ (c) MI-09 (**4**),^27^ (d) vildagliptin (**5**),^44^ and saxagliptin (**6**), and (e) selected reported nirmatrelvir derivatives (**7a–d**).^19^

Considerable efforts for the development of SARS-CoV-2 M^pro^ inhibitors have focused on substrate-based inhibitors, which react covalently with the nucleophilic thiolate of the catalytically active Cys145 of M^pro^, a strategy that is well precedented for inhibiting viral (and other) proteases.^9^ One noncovalently binding M^pro^ inhibitor, i.e., Shionogi’s ensitrelvir (S-217622, **2**; Figure 2a), has been recently approved for clinical use.^23^

Nirmatrelvir (**1**) is particular amongst reported covalent SARS-CoV-2 M^pro^ inhibitors because it employs a nitrile group as an electrophilic warhead. The nitrile of **1** covalently reacts with Cys145, as shown by crystallography (Figure 1b),^18^ likely to give a complex in which a thioimidate electron pair occupies the M^pro^ oxyanion hole (which is formed by the main chain amide NHs of Cys145 and Gly143; Figure 1b), mimicking binding by tetrahedral intermediates in catalysis.^24^ By contrast, other covalently reacting M^pro^ inhibitors employ different electrophilic warheads such as, e.g., α-ketoamides,^11,25,26^ aldehydes^27−30^ and precursors,^25,26,31^ α-hydroxymethylketones and α-acyloxymethylketones,^32−35^ and Michael acceptors,^10^ among others (Figure 2b,c).^6,7,20,36−43^

The use of a nitrile group as a warhead was apparently key to the successful development of nirmatrelvir (**1**), in part because the nonspecific reactivity of nitriles with off-target nucleophiles may be lower than that of many other available electrophilic warheads and in part because the covalent reaction of **1** with the thiolate of Cys145 is reversible.^1^ The use of nitriles as electrophilic warheads in protease inhibitors has previously been demonstrated to be of clinical use, as shown by vildagliptin and saxagliptin, which are the APIs of clinically used type 2 diabetes therapeutics that inhibit human dipeptidylpeptidase-4 (DPP4) by a reversible covalent reaction of their nitrile group with the active site serine residue of DPP4 (Figure 2d).^44^ Nitriles are also present in investigational therapeutics^45^ and have inter alia been employed as electrophilic warheads in substrate-derived inhibitors of SARS-CoV M^pro^ in 2013.^46^

Researchers at Pfizer have reported structure–activity relationship (SAR) studies on nirmatrelvir, which focused on investigating the effect of the P4 substrate-equivalent residue and of ketobenzothiazole groups substituting the nitrile on inter alia inhibitor potency.^1^ Indeed, only a few additional SAR studies on nitrile warhead-bearing covalent SARS-CoV-2 M^pro^ inhibitors have been reported to date, with these mainly focusing on altering the nirmatrelvir P4 substrate-equivalent residue,^24^ on introducing nitrile electrophilic warheads to other inhibitor scaffolds than nirmatrelvir,^109^ and on using azanitriles as electrophilic warheads for the predicted covalent reaction with the thiolate of Cys145.^47^ Recently, studies on nirmatrelvir derivatives in which the nitrile group has been substituted with alternative electrophilic warheads, including aldehydes, benzyloxy/hydroxymethylketones, Michael acceptors, and α-ketoamide groups, which have been previously used in M^pro^ inhibitors other than nirmatrelvir, have been reported with the derivatives being shown to retain high potency in vitro and in cells (Figure 2e).^19^

The substitution of the nirmatrelvir nitrile group for the isoelectronic alkyne group has not yet been explored. Alkynes are particularly attractive (latent) electrophiles for covalent reactions with nucleophilic cysteine proteases, in part because they are isoelectronic with nitriles and share their linear geometry; however, unlike nitriles, they can be functionalized at their terminal position with substituents that could, in principle, bind the protease S′ sites and thus alter inhibitor binding kinetics and be used to improve selectivity or pharmacokinetic properties. The irreversible covalent reaction of both nonactivated terminal and internal alkynes with the active site cysteine thiols of human and viral cysteine proteases has been reported,^48−52^ but such studies have not been reported with SARS-CoV-2 M^pro^. Amino acid-derived activated bromo alkynes have been identified as a result of a fragment screen that covalently react with Cys145 through a yet unidentified mechanism involving loss of bromide.^53^

It is proposed that the efficiency of the addition of thiols to alkynes is largely proximity-driven, i.e., is promoted by preorganization of the reactants.^48^ The reaction of deubiquitinases (DUBs), including SARS-CoV-2 PL^pro^, with ubiquitin and/or other ubiquitin-like protein modifiers (e.g., ISG15) bearing C-terminal nonactivated terminal alkynes has been extensively studied.^49−51,54^ Such activity probes react efficiently with the DUB active site cysteine thiolate, in part, likely because of the small size of the alkyne group, which fits well into the relatively tight DUB active sites and because of the efficient (allosteric) binding of ubiquitin or ubiquitin-like modifiers by DUBs, which position the alkyne electrophile close to the nucleophilic cysteine thiolate.^50^ The use of C-terminally alkynylated ubiquitin and ISG15 activity probes has found widespread application, in part because of the relatively low electrophilicity of alkynes, minimizing the reaction with off-target nucleophiles lacking a templating effect. It has been recently shown that the template effect required to enable efficient reactions of alkynes with nucleophilic thiols is not limited to relatively large protein components, but extends to small-molecule enzyme inhibitors, i.e., an alkyne derivative of the nitrile-bearing investigational drug odanacatib was shown to irreversibly react with the nucleophilic thiolate of a human cathepsin.^48^

Here, we report studies which reveal that derivatives of nirmatrelvir (**1**) bearing activated and nonactivated alkynes, as well as alkyne derivatives of other substrate-derived peptidomimetic M^pro^ inhibitors, are efficient covalent M^pro^ inhibitors, both against isolated M^pro^ enzyme and M^pro^ in cells. The mechanism of inhibition was investigated using crystallography and mass spectrometry (MS) and was shown to involve covalent reactions of the thiolate of the M^pro^ active site Cys145 with the alkynes.

## Results

### Nirmatrelvir Derivatives with a Nonactivated Terminal Alkyne Inhibit M^pro^

**Scheme 1:**
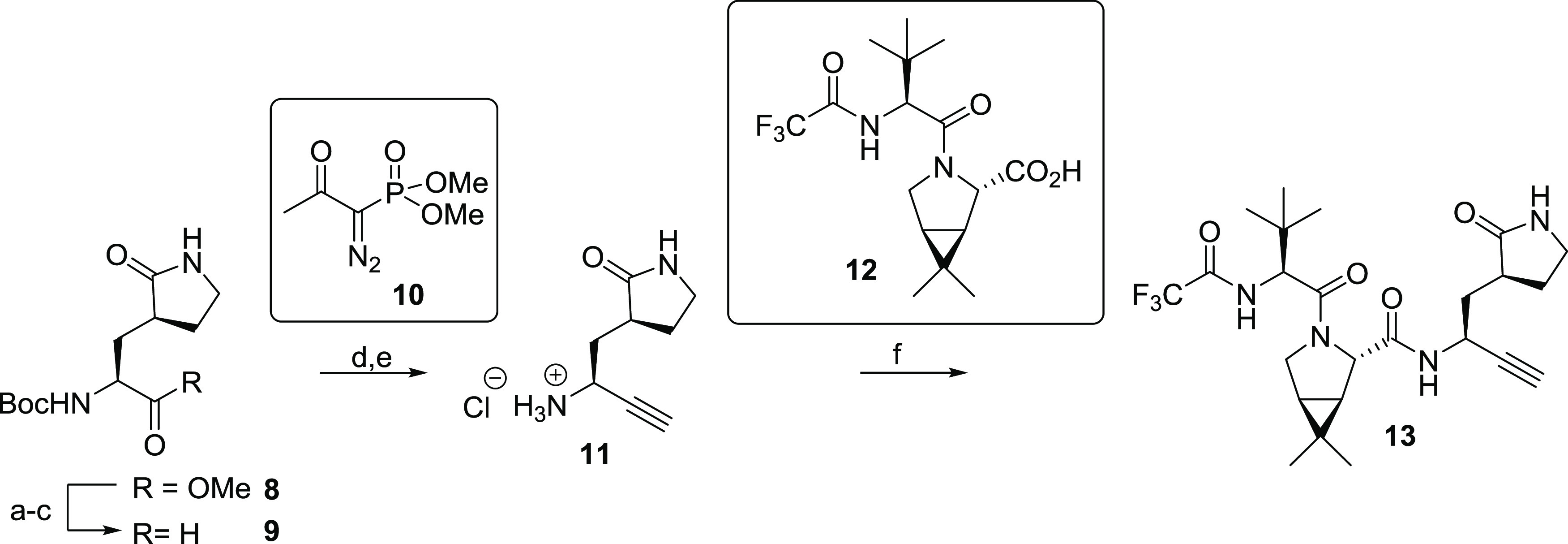
Synthesis of Alkyne-Bearing Nirmatrelvir Derivative **13** Reagents and conditions: (a) LiOH, THF/H_2_O, 0 °C to room temperature (rt), 83%; (b) Me(OMe)NH·HCl, 1,1′-carbonyldiimidazole (CDI), ^*i*^Pr_2_NEt, CH_2_Cl_2_, rt, 58%; (c) LiAlH_4_, THF/Et_2_O, −78 °C, 93%; (d) **10**,^59,60^ K_2_CO_3_, MeOH, rt, 43%; (e) HCl (4 M in dioxane), CH_2_Cl_2_, rt, app. quant.; (f) **12**,^1^ COMU,^55^*N*-methylmorpholine (NMM), DMF/CH_2_Cl_2_, 0 °C to rt, 17%.

To investigate the capacity of using nonactivated terminal alkynes as electrophilic warheads to covalently inhibit SARS-CoV-2 M^pro^, the nirmatrelvir-derived alkyne **13** was synthesized by COMU^55^-mediated amide coupling of the reported acid **12**(1) with alkyne **11** (17% yield following HPLC purification; Scheme 1). Note that the comparatively low yield is a result of material loss during HPLC purification, which was required to obtain sufficiently pure materials when reactions were performed on a laboratory scale; the purification process can likely be optimized to avoid the use of HPLC when reactions are performed on a larger scale, as done by Pfizer researchers during their synthesis of **1**.^1^

Alkyne **11** was synthesized in five steps from the commercially sourced methylester **8**, i.e., saponification, Weinreb amide formation,^56^ LiAlH_4_-mediated reduction to the reported aldehyde **9**,^57,58^ alkynylation of **9** using the Ohira–Bestmann reagent **10**,^59,60^ and Boc deprotection (Scheme 1). An alternative four-step synthesis of **11** involving the LiBH_4_-mediated reduction of **8** to the corresponding alcohol, followed by oxidation to **9**(57,58) using Dess–Martin periodinane,^61^ alkynylation of **9** using **10**, and Boc deprotection was less reproducible and resulted in higher levels of C-α epimerization at the aldehyde stage, as evidenced by ^1^H NMR analysis.

The half-maximum inhibitory concentrations (IC_50_ values) of nirmatrelvir (**1**) and nirmatrelvir alkyne **13** were determined with purified recombinant SARS-CoV-2 M^pro^ using a reported solid-phase extraction coupled to mass spectrometry (SPE-MS)-based M^pro^ inhibition assay that directly monitors M^pro^-catalyzed hydrolysis of a synthetic 37-mer oligopeptide substrate based on the sequence of the N-terminal M^pro^ self-cleavage site (ALNDFSNSGSDVLYQPPQTSITSAVLQ/SGFRKMAFPS-NH_2_; “/” indicates the cleavage site).^43,62^ Note that the N-terminally acetylated C-terminal product peptide (Ac-SGFRKMAFPS-NH_2_) was employed as an internal standard to avoid the identification of false positive hits due to inhibitor-induced suppression of ionization of the product peptide (Supporting Figures S1 and S2).

The results reveal that alkyne **13** inhibits M^pro^ approximately 5-fold less efficiently than nirmatrelvir (**1**), highlighting the potential of nonactivated terminal alkynes to efficiently inhibit M^pro^ (IC_50_ ≤ 0.025 μM for **1** and ∼0.14 μM for **13**; Table 1, entries i and ii). Note, however, that the reduction in potency of **13** compared to **1** may be more pronounced because **1** inhibits M^pro^ at concentrations close to the lowest detection limit of the SPE-MS assay.

**Table 1:**
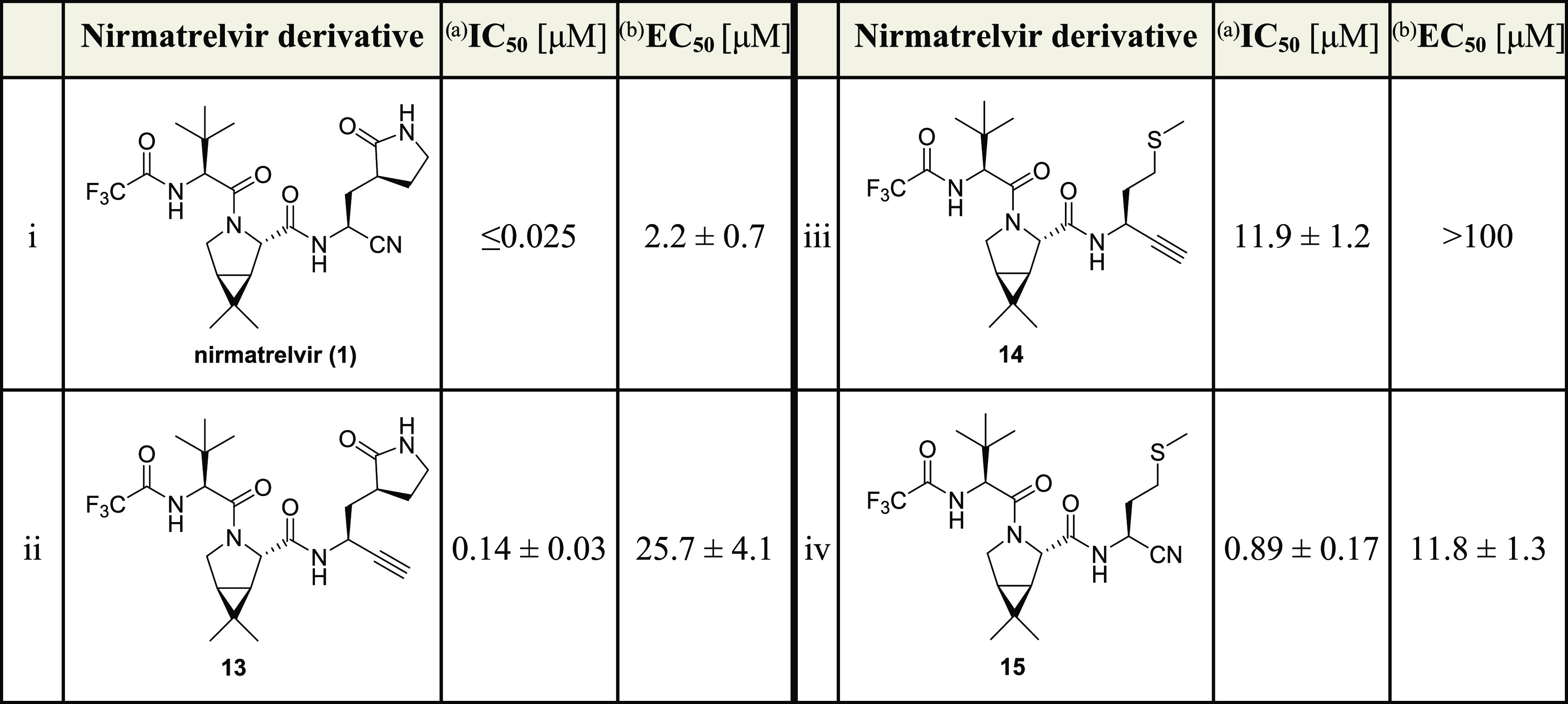
Inhibition of SARS-CoV-2 M^pro^ by Nirmatrelvir Derivatives Bearing a Terminal Alkyne

a Inhibition assays were performed using SPE-MS as described employing SARS-CoV-2 M^pro^ (0.05 μM) and ALNDFSNSGSDVLYQPPQTSITSAVLQ/SGFRKMAFPS-NH_2_ as a substrate (2.0 μM) in the presence of an internal standard (Supporting Figures S1 and S2).^43,62^ The results are means of three independent repeats, each composed of technical duplicates (*n* = 3; mean ± standard deviation, SD). Representative dose–response curves are shown in Figure 3a.

b Representative dose–response curves for cell-based assays are shown in Supporting Figure S3a. The results are means of three independent repeats (*n* = 3; mean ± SD).

The ability of nirmatrelvir alkyne derivative **13** to inhibit SARS-CoV-2 progression in infected VeroE6 cells was assessed in the absence of CP-100356, which is reported to inhibit the efflux pump-mediated removal of **1** from cells.^1^ Importantly, the half-maximum effective concentrations (EC_50_ values) revealed that alkyne **13** inhibits SARS-CoV-2 progression in cells, however, ∼11-fold less efficiently than nirmatrelvir (EC_50_ ∼ 2.2 μM for **1** and ∼25.7 μM for **13**; Table 1, entries i and ii), a difference consistent with the studies with isolated M^pro^. Alkyne **13** was apparently not cytotoxic by the 3-(4,5-dimethylthiazol-2-yl)-2,5-diphenyltetrazolium bromide (MTT) cell viability assay (Supporting Table S1). Note that further biological investigations are required because the interpretation of cell-based SARS-CoV-2 progression inhibition data can be challenging and metabolism of alkynes in animals may influence toxicity (Figure 3).^63^

**Figure 3:**
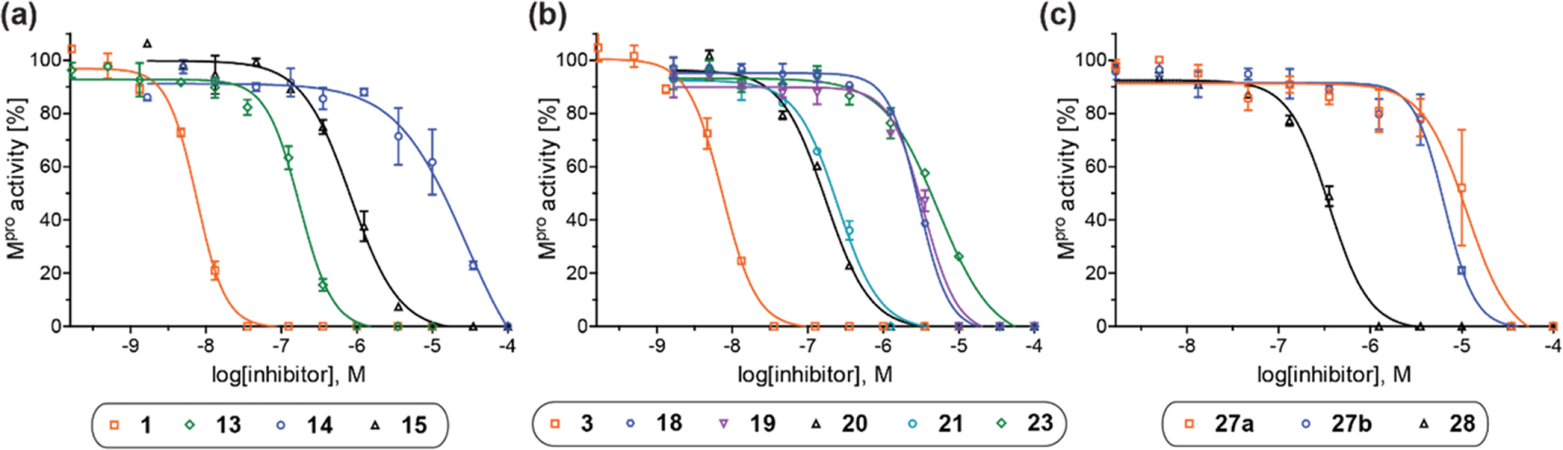
Dose–responses observed for nitrile- or alkyne-bearing SARS-CoV-2 M^pro^ inhibitors. Representative dose–response curves of M^pro^ inhibitors shown in (a) Table 1, (b) Table 2, and (c) Table 3. High *Z*′-factors^66^ (>0.5 for each inhibition plate) indicate excellent solid-phase extraction coupled to mass spectrometry (SPE-MS) assay quality (Supporting Figure S5).

Nirmatrelvir alkyne derivatives with a methionine at the substrate P1 equivalent position were of particular interest for SAR studies, because efficient SARS-CoV-2 M^pro^ inhibitors with a methionine at this position have been reported.^25,64^ Thus, the alkyne **14** (Table 1, entry iii) was synthesized from the reported methionine alkyne building block corresponding to **11**(65) to probe the effect of the P1 substrate-equivalent substituent on inhibitor potency (Supporting Figure S4). To enable comparison with nirmatrelvir (**1**), the corresponding methionine nitrile analogue of nirmatrelvir (**15**; Table 1, entry iv) was synthesized from commercially sourced methionine amide by adapting Pfizer’s reported synthesis of **1** (Supporting Figure S4).^1^

Inhibition assays reveal that substituting the P1 substrate-equivalent glutamine derivative of nirmatrelvir (**1**) for a methionine derivative, as in **15**, results in a ∼35-fold decrease in inhibition potency vs isolated SARS-CoV-2 M^pro^ (IC_50_ ≤ 0.025 μM for **1** and ∼0.9 μM for **15**; Table 1, entries i and iv) but only in a ∼5-fold decrease in inhibiting SARS-CoV-2 progression in infected VeroE6 cells compared to **1** (EC_50_ ∼ 2.2 μM for **1** and ∼11.8 μM for **15**; Table 1, entries i and iv). These observations highlight the importance of the cycloglutamine P1 Gln analogue at the P1 substrate-equivalent residue present in **1** and other inhibitors for efficient inhibition of isolated M^pro^. Interestingly, nitrile **15** inhibited SARS-CoV-2 progression in infected cells ∼2-fold more efficient than alkyne **13** (EC_50_ ∼ 11.8 μM for **15** and ∼25.7 μM for **13**; Table 1, entries iv and ii), despite its ∼6-fold reduced potency in inhibiting isolated M^pro^ (Table 1). Note that the alkyne methionine derivative **14** was ∼85-fold less efficient in inhibiting isolated M^pro^ than **13** (IC_50_ ∼ 11.9 μM, Table 1, entry iii), indicating that nonactivated terminal alkynes may be of interest as electrophilic warheads in M^pro^ inhibitors other than those related to **1**.

### Alkyne Derivatives of Investigational COVID-19 Therapeutics Inhibit M^pro^

**Scheme 2:**
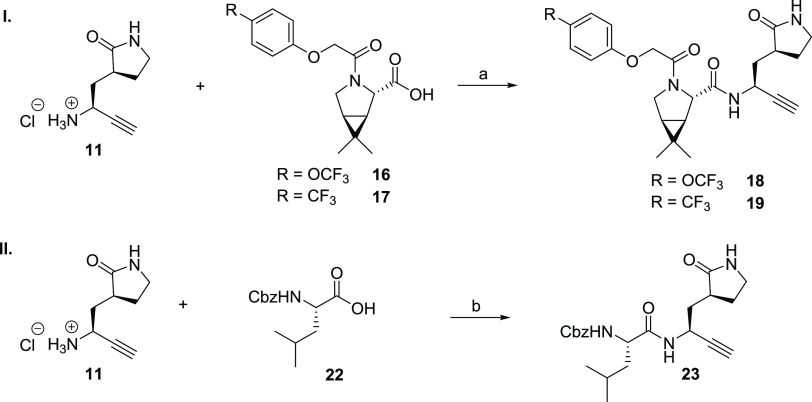
Synthesis of Alkyne Derivatives of the Investigational COVID-19 Therapeutics GC376 (**3**) and MI-09 (**4**) Reagents and conditions: (a) **16**(27) or **17**, COMU,^55^ NMM, DMF/CH_2_Cl_2_, 0 °C to rt, 14 and 13%, respectively; (b) COMU,^55^ NMM, DMF/CH_2_Cl_2_, 0 °C to rt, 33%.

To investigate the potential of alkynes as electrophilic warheads of substrate-derived SARS-CoV-2 M^pro^ inhibitors other than those closely related to nirmatrelvir (**1**), we synthesized alkyne derivatives of the aldehyde MI-09 (**4**, Figure 2c), which has been reported to efficiently inhibit M^pro^ both in vitro and in animal model studies.^27^ Note that the structure of MI-09 (**4**) differs from that of nirmatrelvir (**1**), both with respect to its electrophilic warhead and its P3/P4 substrate-equivalent substituents, while both molecules have the same P1 and P2 substrate-equivalent residues. The MI-09 alkyne derivatives **18** and **19** were synthesized by COMU^55^-mediated amide coupling of alkyne **11** with the reported acid **16**(27) or **17** in 14 and 13% yield, respectively, following HPLC purification (Scheme 2). For comparison, the corresponding nitrile-bearing MI-09 derivatives **20** and **21**, which have not been previously reported, were synthesized according to procedures for the synthesis of **1** (Table 2 and Supporting Figure S6).^1^

**Table 2:**
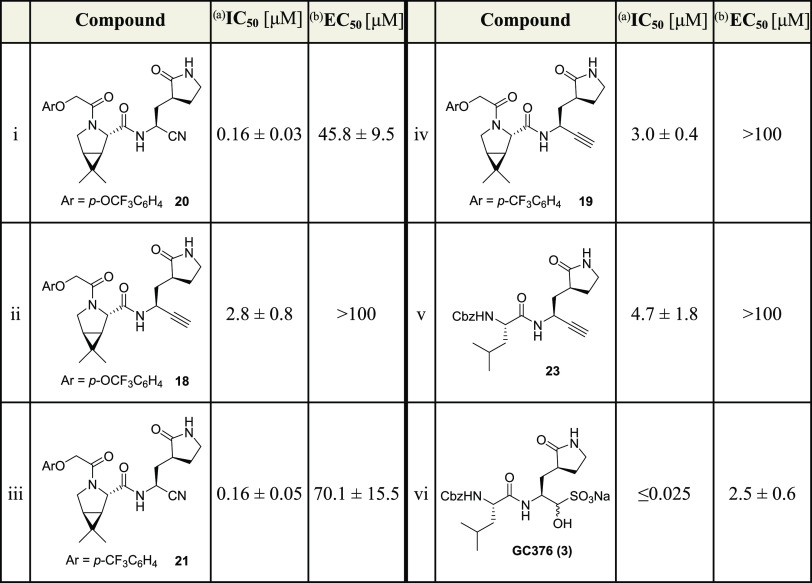
Inhibition of SARS-CoV-2 M^pro^ by Alkyne Derivatives of Investigational COVID-19 Small-Molecule Therapeutics^a^,^b^

a Inhibition assays were performed using SPE-MS as described employing SARS-CoV-2 M^pro^ (0.05 μM) and ALNDFSNSGSDVLYQPPQTSITSAVLQ/SGFRKMAFPS-NH_2_ as a substrate (2.0 μM) in the presence of an internal standard (Supporting Figures S1 and S2).^43,62^ The results are means of three independent repeats, each composed of technical duplicates (n = 3; mean ± SD). Representative dose–response curves are shown in Figure 3b.

b Representative dose–response curves for cell-based assays are shown in Supporting Figure S3b. The results are means of three independent repeats (n = 3; mean ± SD). Cbz: −C(O)OCH_2_C_6_H_5_.

The alkyne derivative **23** of GC376 (**3**, Figure 2b), which is a broad-spectrum pan-coronavirus M^pro^ inhibitor,^25,26,31^ was synthesized by COMU^55^-mediated amide coupling of commercially sourced Z-Leu-OH (**22**) with alkyne **11** in 33% yield following HPLC purification as a 5:1 mixture of diastereomers (Scheme 2).

The MI-09-derived nitrile **20** inhibits isolated SARS-CoV-2 M^pro^ less efficiently than nirmatrelvir (**1**), but with a similar efficiency as the nirmatrelvir alkyne derivative **13** (IC_50_ ∼ 0.16 μM, Table 2, entry i). By contrast, the MI-09 alkyne derivative **18** was ∼17-fold less potent than **20** and ∼20-fold less potent than **13** (IC_50_ ∼ 2.8 μM, Table 2, entry ii), in accord with a similar trend observed for **1** and **13**, which is about 5-fold less efficient than **1** (Table 1). Similar to nitrile **21**, which inhibits M^pro^ with comparable potency as nitrile **20** (IC_50_ ∼ 0.16 μM, Table 2, entry iii), its alkyne derivative **19** was as potent as the corresponding alkyne **18**, within experimental error (IC_50_ ∼ 3.0 μM, Table 2, entry iv). Although alkynes **18** and **19,** as well as nitriles **20** and **21,** were potent inhibitors of isolated SARS-CoV-2 M^pro^, they did not efficiently inhibit SARS-CoV-2 progression in infected VeroE6 cells (Table 2). The ability of nitriles **20** and **21** to inhibit SARS-CoV-2 progression in infected VeroE6 cells was ∼20- to ∼30-fold reduced than that of nirmatrelvir (**1**), while alkynes **18** and **19** did not show any inhibitory activity over the tested concentration range, highlighting the importance of the P3 and P4 substrate-equivalent positions for efficient inhibition, in accord with previous observations by Pfizer.^1^

The GC376-derived alkyne **23** inhibited M^pro^ about 2-fold less efficiently than the MI-09-derived alkynes **18** and **19** (IC_50_ ∼ 4.7 μM, Table 2, entry i) and more than a 100-fold less efficiently than GC376 (Table 2, entry vi). GC376-derived alkyne **23** inhibited M^pro^ also about ∼30-fold less efficient than the nirmatrelvir-derived alkyne **13**, an observation which may in part reflect its less rigid structure compared to nirmatrelvir (**1**), rendering it less optimized for efficient binding to the M^pro^ active site, and in part its reduced purity (5:1 diastereomeric mixture). Note that while GC376 (**3**) inhibited SARS-CoV-2 progression in infected VeroE6 cells with similar efficiency to that reported (EC_50_ ∼ 2.5 μM, Table 2, entry vi),^25,26^ alkyne **23** did not show any inhibitory activity over the tested concentration range (Table 2, entry v). Nonetheless, the results clearly illustrate the general utility of nonactivated terminal alkynes as electrophilic warheads for inhibitors of isolated M^pro^, including those which are attached to less tight-binding inhibitor scaffolds than that of **1**.

### Nirmatrelvir Derivatives with C-Terminal-Derivatized Alkynes Inhibit M^pro^

The reactivity of alkynes with nucleophiles may be altered by introducing substituents other than a proton at the terminal position. In the case of M^pro^ inhibition, substituents of interest include sterically bulky groups that may bind in the S′ pockets and those which alter the electronic properties of the alkyne and thus its reactivity and selectivity profile with respect to the reaction with nucleophiles, e.g., electron-withdrawing or -donating groups.

**Scheme 3:**
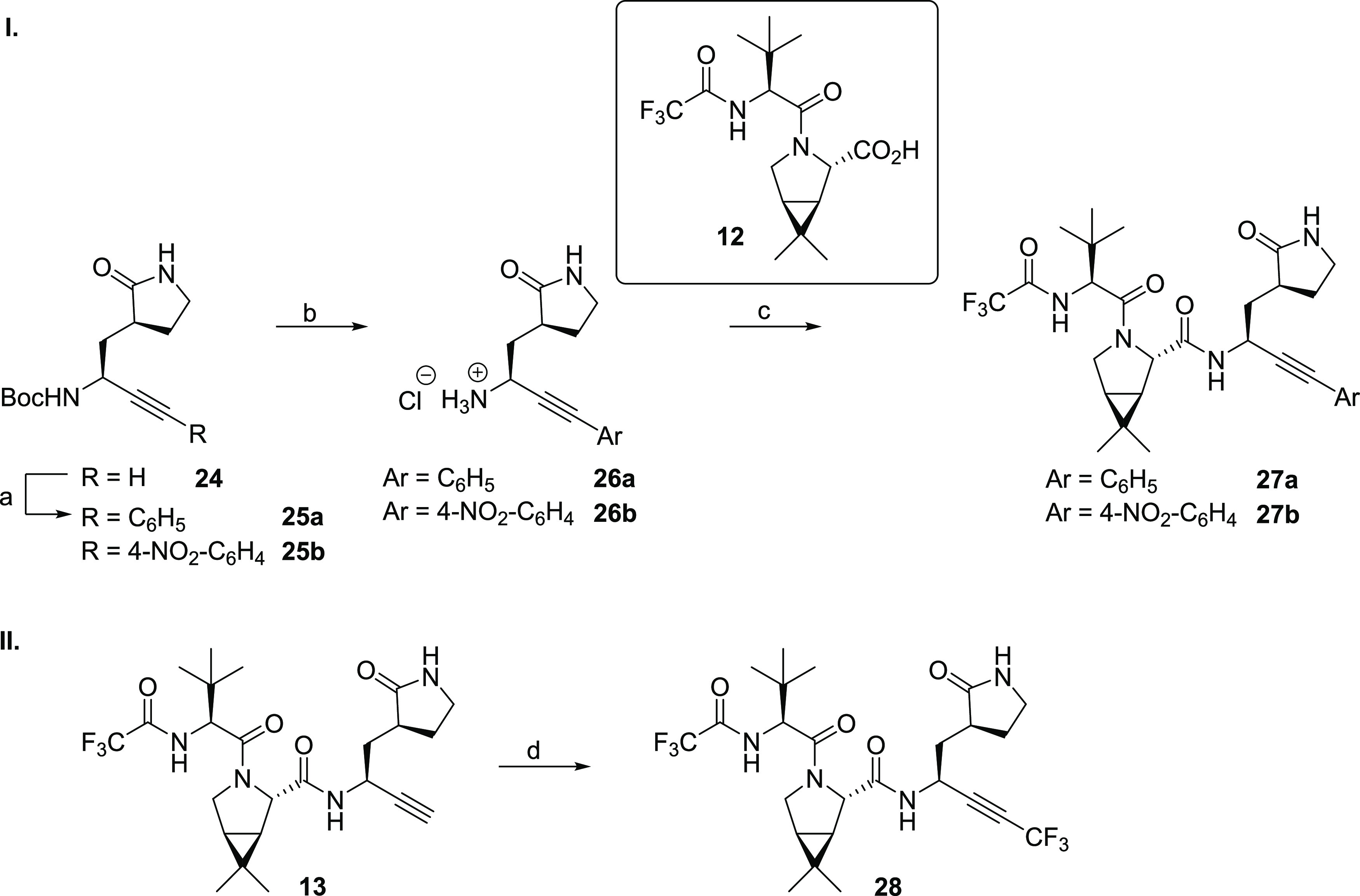
Synthesis of Nirmatrelvir Derivatives Bearing an Internal Alkyne Reagents and conditions: (a) aryl iodide, CuI (5 mol %), PdCl_2_(PPh_3_)_2_ (2.5 mol %), NEt_3_/THF, 80 °C, 46% (**25a**) or 44% (**25b**); (b) HCl (4 M in dioxane), CH_2_Cl_2_, rt, app. quant.; (c) **12**,^1^ COMU,^55^ NMM, DMF/CH_2_Cl_2_, 0 °C to rt, 17% (**27a**) or 11% (**27b**); (d) TMSCF_3_, CuI, K_2_CO_3_, *N*,*N*,*N*′,*N*′-tetramethylethylenediamine (TMEDA), DMF, rt, air, 26%.

Initially, we investigated derivatives **27a** and **27b** bearing aryl-substituted alkynes as SARS-CoV-2 M^pro^ inhibitors, in part because previous work has shown that the S2′ pocket can accommodate aromatic groups. Derivatives **27a** and **27b** were synthesized by Sonogashira coupling^67,68^ of **24**, an intermediate in the synthesis of building block **11** (Scheme 1), with aryl iodides, followed by Boc deprotection, by COMU^55^-mediated amide coupling with the reported acid **12**,^1^ and then HPLC purification (Scheme 3).

In addition to the nirmatrelvir derivatives **27a** and **27b** bearing aryl-substituted internal alkynes, the terminal alkyne of **13** was capped with an electron-withdrawing CF_3_ group to enhance its electrophilicity; the presence of an *N*-trifluoroacetyl group in nirmatrelvir was also important in its in vivo optimization.^1^ The synthesis of **28** was achieved by employing a reported Cu(I)-mediated CH activation reaction (Scheme 3),^69^ which was performed at a late stage in the synthesis, as it was challenging to carry a CF_3_-capped alkyne derivative of **11** through the entire synthesis, as done for the aryl-capped alkynes **27a** and **27b**. Thus, the reaction of **13** with TMSCF_3_ afforded **28** in 26% yield following HPLC purification.

The unoptimized phenyl-capped nirmatrelvir alkyne derivatives **27a** and **27b** inhibit isolated SARS-CoV-2 M^pro^ ∼80- to ∼50-fold less efficiently than the terminal nirmatrelvir alkyne derivative **13** (IC_50_ ∼ 11.0 and ∼7.1 μM, respectively; Table 3, entries ii and iii). By contrast, the CF_3_-capped alkyne **28** inhibits M^pro^ with a similar potency as the underivatized terminal alkyne **13** (IC_50_ ∼ 0.22 μM, Table 3, entry iv), within experimental error, an observation which is remarkable considering that the size of the CF_3_ group ranges in between those of isopropyl and *tert*-butyl groups.^70,71^ Thus, the loss of inhibition potency upon capping the alkyne of **13** with the tested phenyl groups is likely to be, in a substantial part, a result of electronic effects.

**Table 3:**
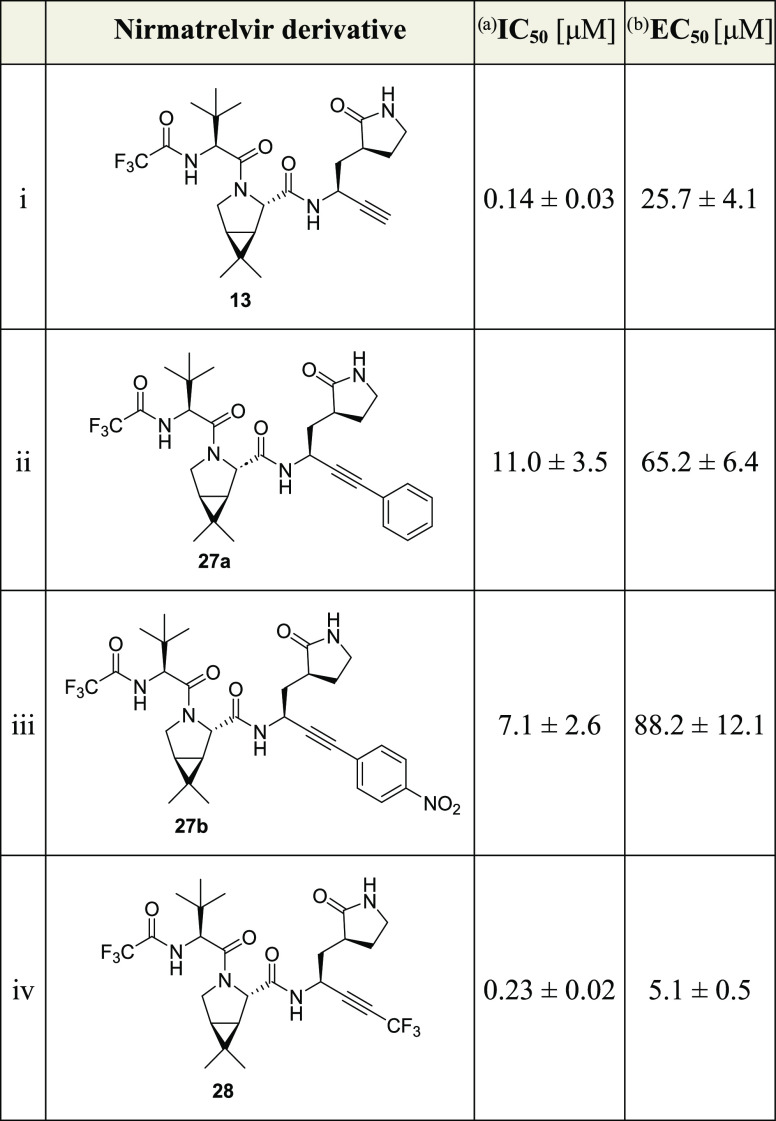
Inhibition of SARS-CoV-2 M^pro^ by Nirmatrelvir Derivatives Bearing a C-Terminal Derivatized Alkyne

a Inhibition assays were performed using SPE-MS as described employing SARS-CoV-2 M^pro^ (0.05 μM) and ALNDFSNSGSDVLYQPPQTSITSAVLQ/SGFRKMAFPS-NH_2_ as a substrate (2.0 μM) in the presence of an internal standard (Supporting Figures S1 and S2).^43,62^ The results are means of three independent repeats, each composed of technical duplicates (*n* = 3; mean ± SD). Representative dose–response curves are shown in Figure 3c.

b Representative dose–response curves for cell-based assays are shown in Supporting Figure S3c. The results are means of three independent repeats (*n* = 3; mean ± SD).

The phenyl-capped alkyne derivatives **27a** and **27b** inhibit SARS-CoV-2 progression in infected VeroE6 cells ∼2- to ∼3-fold less efficiently than the terminal alkyne **13** (EC_50_ ∼ 65.2 and ∼88.2 μM, respectively; Table 3, entries ii and iii). By contrast, the CF_3_-capped alkyne **28** inhibits SARS-CoV-2 progression in infected VeroE6 cells ∼5-fold more efficiently than the terminal alkyne **13** (EC_50_ ∼ 5.1 μM, Table 3, entry iv), even though both **13** and **28** inhibit isolated SARS-CoV-2 M^pro^ with similar potency; this observation may reflect the enhanced electrophilicity of **28** or other, currently unknown, factors such as, e.g., improved pharmacodynamic and/or pharmacokinetic properties. Importantly, the results reveal that capping the terminal alkyne of **13** (and likely other terminal alkynes) appears to be a suitable strategy to improve the activity of alkyne M^pro^ inhibitors in cells. Note that while alkyne **28** inhibits SARS-CoV-2 progression in infected VeroE6 cells ∼2-fold less efficiently than nirmatrelvir (**1**) and GC376 (**3**), its potency is ∼2-fold higher than that of nirmatrelvir methionine nitrile **15**, suggesting that the structure of **13** or **28** can be further optimized to increase cellular activity further, potentially to achieve similar or better inhibitory activity in cells than observed for **1** and **3**.

### Alkyne-Bearing M^pro^ Inhibitors Inhibit Ser144Ala SARS-CoV-2 M^pro^ Catalysis

The Ser144Ala SARS-CoV-2 M^pro^ variant has been observed in humans and is reported to reduce the inhibition efficacy of nirmatrelvir by >10-fold.^72,73^ Ser144 is located on the loop that forms the M^pro^ oxyanion hole and is adjacent to the nucleophilic Cys145, the backbone NH of which, along with those of Gly143 and Ser144, helps stabilize the tetrahedral oxyanionic intermediates during catalysis. Ser144 variations can reduce the potency of nirmatrelvir, and it has thus been proposed that Ser144 M^pro^ variations may enable SARS-CoV-2 to develop resistance toward nirmatrelvir treatment.^72,73^ We therefore investigated the efficacy of the nirmatrelvir alkyne derivatives against the Ser144Ala M^pro^ variant using SPE-MS inhibition assays (Supporting Table S2).

The results show that the Ser144Ala variant is catalytically less active than wildtype (WT) M^pro^ and that nirmatrelvir (**1**) inhibits Ser144Ala M^pro^ less efficiently than WT M^pro^ (∼4-fold), in accord with previous reports,^72,73^ likely in both cases because of impaired oxyanion stabilization. Importantly, the results show that the nirmatrelvir alkyne derivatives **13**, **14**, **27a**, **27b**, and **28** also inhibited Ser144Ala M^pro^, however, ∼2- to ∼4-fold less efficiently than WT M^pro^ (Supporting Table S2). Interestingly, the MI-09^27^-derived nitrile **20** inhibits Ser144Ala M^pro^ ∼20-fold less efficiently than WT M^pro^, whereas the corresponding alkyne **18** inhibits Ser144Ala M^pro^ only ∼3-fold less efficiently than WT M^pro^. Although further work is required, these results reveal the effect of catalytically relevant M^pro^ active site variations, in particular substitution of an oxyanion hole-related residue (Ser144), to differentially impact on the relative inhibition efficiency of alkyne- and nitrile-based inhibitors and, by implication, of other electrophilic warheads.

### Alkyne-Bearing M^pro^ Inhibitors Do Not Affect SARS-CoV-2 PL^pro^ Catalysis

Since the covalent reaction of the active site cysteine Cys111 of SARS-CoV-2 PL^pro^ with alkyne electrophiles has been described,^51,54^ the selectivity of the synthetic alkyne SARS-CoV-2 M^pro^ inhibitors **13**, **14**, **18**, **19**, **23**, **27a**, **27b**, and **28** was determined using reported SPE-MS PL^pro^ inhibition assays, which directly monitor PL^pro^-catalyzed hydrolysis of a substrate-derivate oligopeptide.^74^ In addition, the nitrile M^pro^ inhibitors **15**, **20**, and **21** were investigated for PL^pro^ inhibition. None of the tested M^pro^ inhibitors inhibited SARS-CoV-2 PL^pro^, in accord with the reported lack of PL^pro^ inhibition by nirmatrelvir (Supporting Table S3).^74^

### Nirmatrelvir Alkyne Derivative **13** Inhibits M^pro^ by Covalent Reaction with Cys145

The mechanism by which the alkyne **13** inhibits SARS-CoV-2 M^pro^ was next investigated by crystallography. Twelve microcrystals, which were obtained by cocrystallization of M^pro^ with **13**, were analyzed using the VMXm microfocus beamline at the Diamond Light Source synchrotron;^75,76^ the diffraction data were merged, and a structure of M^pro^ in complex with **13** was solved by molecular replacement using a reported M^pro^ structure (PDB ID: 6YB7) as a search model (*P*2_1_2_1_2 space group, 1.9 Å resolution; Supporting Figure S7 and Table S4). The overall M^pro^ protein folds in the M^pro^:**13** complex and the reported M^pro^:**1** complex (PDB ID: 7TE0(22)) structures are very similar (RMSD = 0.313 Å, Supporting Figure S7).

**Figure 4:**
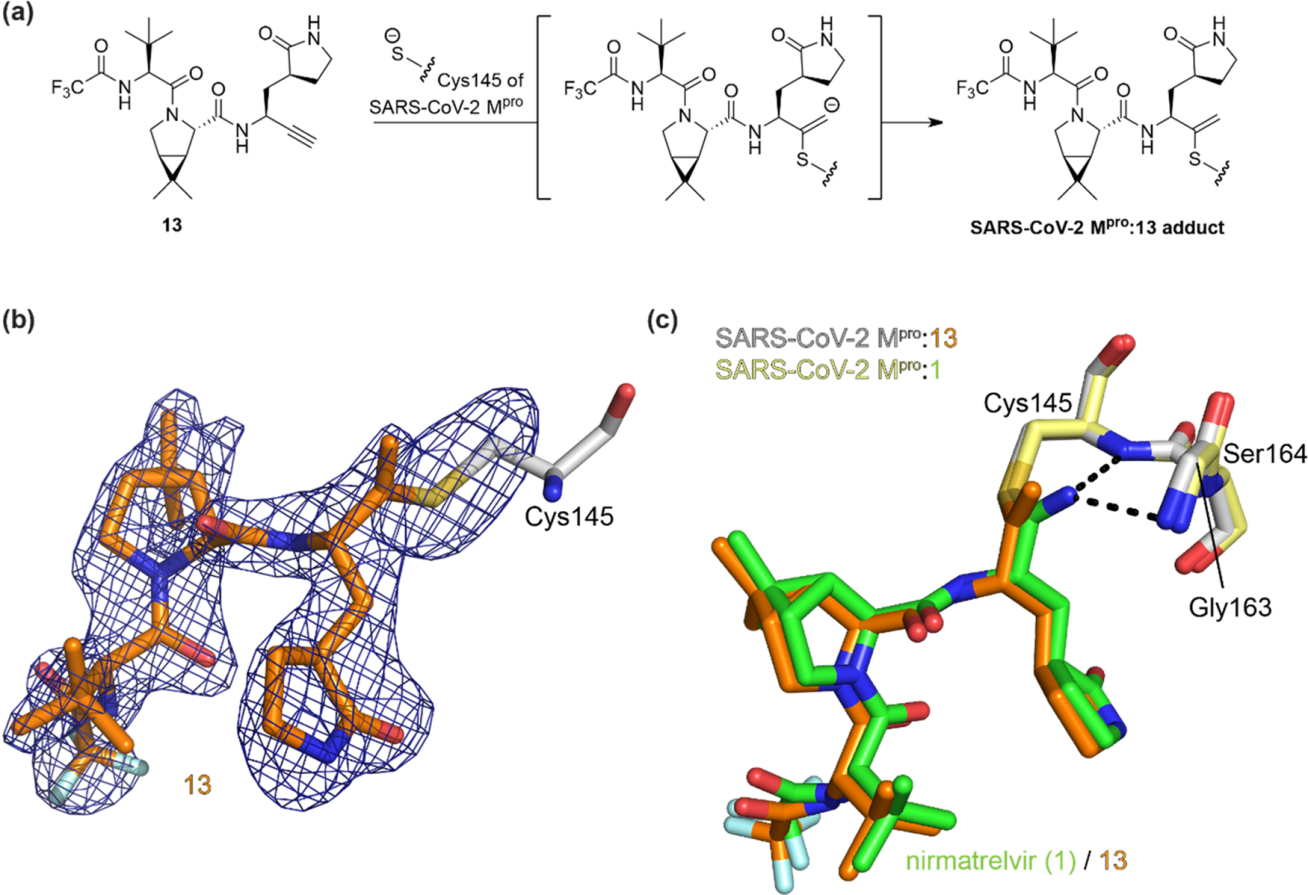
Crystallographic evidence that the alkyne of the nirmatrelvir derivative **13** reacts covalently with the nucleophilic thiolate of M^pro^ Cys145. Color code: SARS-CoV-2 M^pro^, gray; carbon backbone of **13** in complex with M^pro^ is in orange; oxygen, red; nitrogen, blue; sulfur, yellow; and fluorine, light blue. (a) Reaction of M^pro^ with alkyne **13**; (b) representative OMIT electron density map (*mF*_o_–*DF*_c_) contoured to 3σ around Cys145 and **13** in complex with M^pro^ (PDB ID: 8B2T); (c) superimposition of a view from the M^pro^:**13** complex structure (PDB ID: 8B2T) with the reported M^pro^:nirmatrelvir (**1**) complex structure (pale yellow: M^pro^; green: carbon backbone of **1** in complex with M^pro^; PDB ID: 7TE0(22)) showing the interaction of **1** but not **13** with residues forming the oxyanion hole, i.e., Cys145 and Gly143.

The M^pro^:**13** complex structure unambiguously shows that the nucleophilic thiolate of Cys145 covalently reacts with the terminal alkyne group of **13** at the more electrophilic internal alkyne position (distance of the Cys145 S-atom to the internal C-atom of the vinyl group: 1.8 Å; Figure 4a). The groups at the P1-4 substrate-equivalent positions of **13** occupy the same M^pro^ substrate binding pockets as observed for **1**, i.e., the cycloglutamine group occupies the S1 pocket, the bicyclic leucine isostere occupies the hydrophobic S2 pocket, the *tert*-butyl group is solvent-exposed, and the trifluoroacetamide occupies the S4 pocket, and are positioned to interact with M^pro^ via similar H-bonding interactions as observed for **1** (Supporting Figure S8). In contrast with the M^pro^:**1** complex structure, in the alkyne **13** structure, the terminal olefin C-atom of the vinyl thioether formed by the reaction with Cys145 is not positioned in the oxyanion hole (distances of the terminal olefin C-atom to the Cys145 and Gly143 main chain N-atoms: 3.6 and 3.8 Å, respectively) compared to the thioimidate nitrogen in **1** (distances of the thioimidate N-atom to the Cys145 and Gly143 main chain N-atoms: 3.1 and 3.3 Å, respectively; PDB ID: 7R7H(32)), indicating the absence of H-bonding-type interactions of the intermediate vinyl anion with the oxyanion hole in the M^pro^:**13** complex (Figure 4). Although care should be taken in correlating the precise mechanism in solution with crystal structures, this observation suggests that the proposed vinyl anion intermediate is protonated, potentially by one of the oxyanion hole NH groups or by water. Note that two SARS-CoV-2 M^pro^ complex structures in which the nucleophilic thiolate of Cys145 has reacted with activated bromo alkynes of unspecific protease inhibitors have been reported; in these structures, the terminal olefin C-atom of the vinyl thioether formed with Cys145 is positioned in the oxyanion hole (Supporting Figure S9).^53^ The apparently different binding mode of **13** compared to **1** with respect to the oxyanion hole is of interest, given the differences in relative potencies for some of the nitriles and alkynes versus WT and Ser144Ala M^pro^, as described above.

### Evidence That Nirmatrelvir Alkyne Derivative **13** Is an Irreversible Covalent M^pro^ Inhibitor

It has been reported that the reaction of nirmatrelvir (**1**) with the nucleophilic thiolate of SARS-CoV-2 M^pro^ Cys145 is reversible,^1^ in accord with the reversible reaction of other small-molecule nitrile inhibitors with nucleophilic cysteine or serine residues^32,44,77^ and a mechanism in which the negatively charged thioimide electron pair occupies the M^pro^ oxyanion hole (Figure 1). By contrast, it appears feasible that the initially formed vinyl anion (by the reaction of the nucleophilic thiolate of M^pro^ with the alkyne group of **13**) is protonated as it is oriented away from the oxyanion hole in the M^pro^:**13** complex structure (Figure 4), suggesting that the reaction of **13** with M^pro^ may be irreversible. To investigate this, we initially analyzed the covalent reaction of M^pro^ with synthetic alkyne and nitrile inhibitors (i.e., **1**, **13**–**15**, **18**–**21**, **23**, **27a**, **27b**, and **28**) at a fixed concentration using protein-observed SPE-MS. The results revealed that all of the synthesized nitrile and alkyne inhibitors react covalently and once with M^pro^, likely with the nucleophilic active site Cys145, as supported by crystallographic analysis (Figure 4). Their apparent reaction rates with M^pro^ differed, with nirmatrelvir (**1**) and its alkyne derivative **13** appearing to react fastest with M^pro^ (Supporting Figures S10–S21).

**Figure 5:**
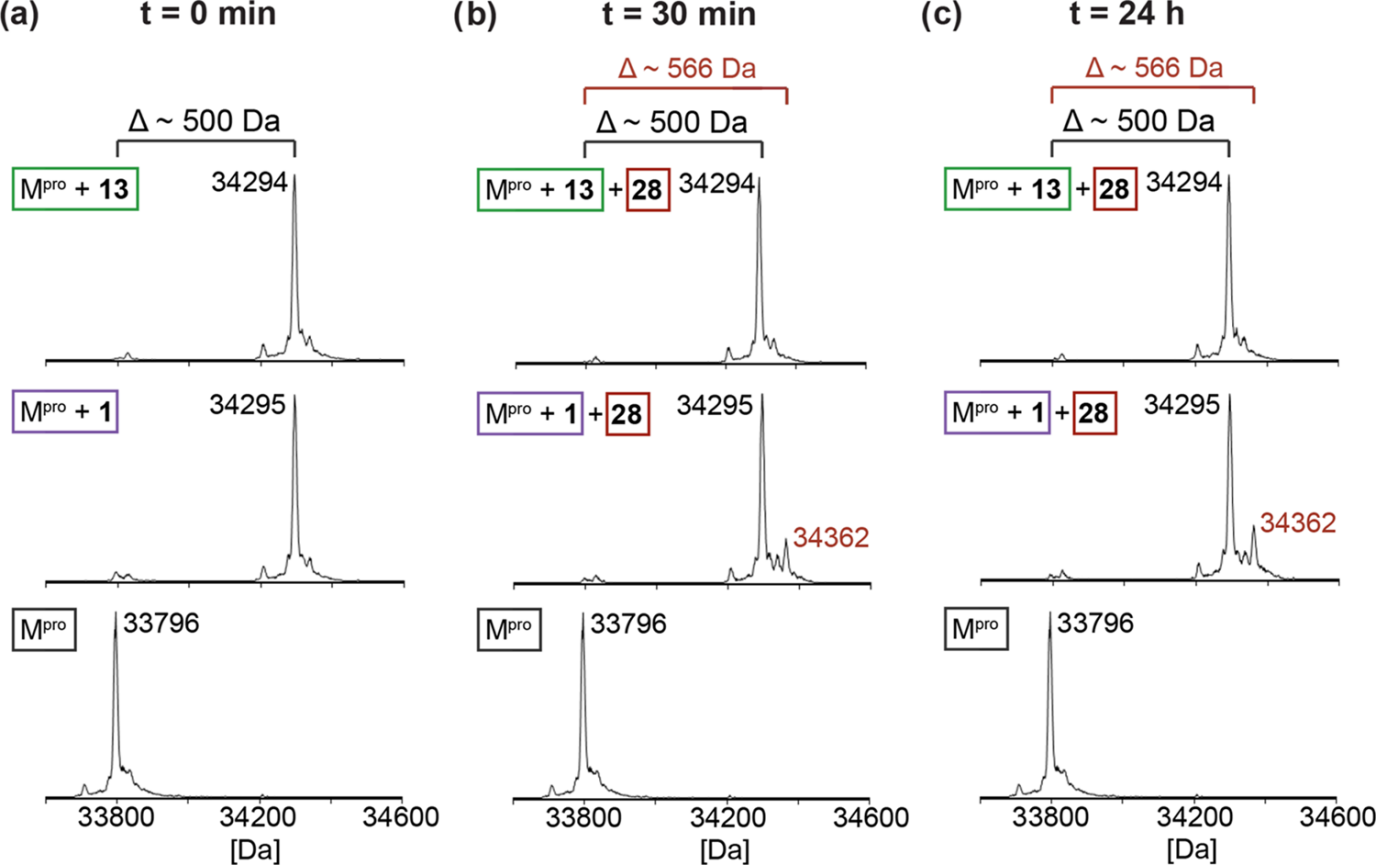
Nirmatrelvir alkyne derivatives inhibit SARS-CoV-2 M^pro^ via covalent reaction with Cys145. (a) Reaction of M^pro^ (bottom) with nirmatrelvir (**1**, center) and its alkyne derivative **13** (top); SPE-MS analysis indicates near-quantitative reaction of **1** or **13** with M^pro^ (∼500 Da mass shifts). (b) Addition of a 10-fold excess of alkyne **28** to the covalent complexes of M^pro^ with **1** or **13**, followed by 30 min incubation; SPE-MS analysis of the mixtures reveals formation of a covalent complexes of M^pro^ with **28** only for the M^pro^ complex with **1** (∼66 Da mass shift, center), but not with **13** (top), as compared with unreacted M^pro^ (bottom). (c) SPE-MS analysis of the mixtures of the covalent complexes of M^pro^ with **1** or **13** with **28** indicates that **28** reacts slowly with the M^pro^ complex with **1** (∼66 Da mass shift, center), but not with **13** (top), as compared with unreacted M^pro^ (bottom). M^pro^ assays were performed using SPE-MS as described in the Experimental Section employing SARS-CoV-2 M^pro^ (2.0 μM) in buffer (20 mM HEPES, pH 7.5).^43,62^

To assess the reversibility of the covalent reaction of M^pro^ with nirmatrelvir (**1**) and alkyne **13**, we then separately incubated M^pro^ with an excess of **1** or **13**, until apparent complete formation of the corresponding covalent M^pro^ adducts was observed by SPE-MS (Figure 5a). Unreacted **1** and **13** were removed from the mixtures, and a 10-fold excess of the CF_3_-bearing alkyne **28** was added to the covalent complexes of M^pro^ with **1** and **13**; competition for binding to M^pro^ was then monitored by SPE-MS as a function of time (Figure 5). Alkyne **28** was used because it is a potent M^pro^ inhibitor, which covalently reacts with Cys145 (Table 3 and Figure 5a) and because its mass differs substantially from the masses of **1** and **13** (by ∼67 Da), thus enabling the differentiation of the M^pro^ complex with **28** from those with **1** or **13**, by SPE-MS. The results revealed that, while the covalent M^pro^ adduct with nirmatrelvir (**1**) slowly exchanges with **28** over time reaching ∼20% exchange 4 h post incubation (Figure 5b,c), the covalent M^pro^ adduct with **13** did not exchange with **28**, even after prolonged incubation (>48 h). Thus, the covalent reaction of M^pro^ with nitriles such as **1** is reversible, whereas the covalent reaction of M^pro^ with alkynes such as **13** is irreversible or at least substantially less reversible than the reaction with nitriles.

## Discussion

**Figure 6:**
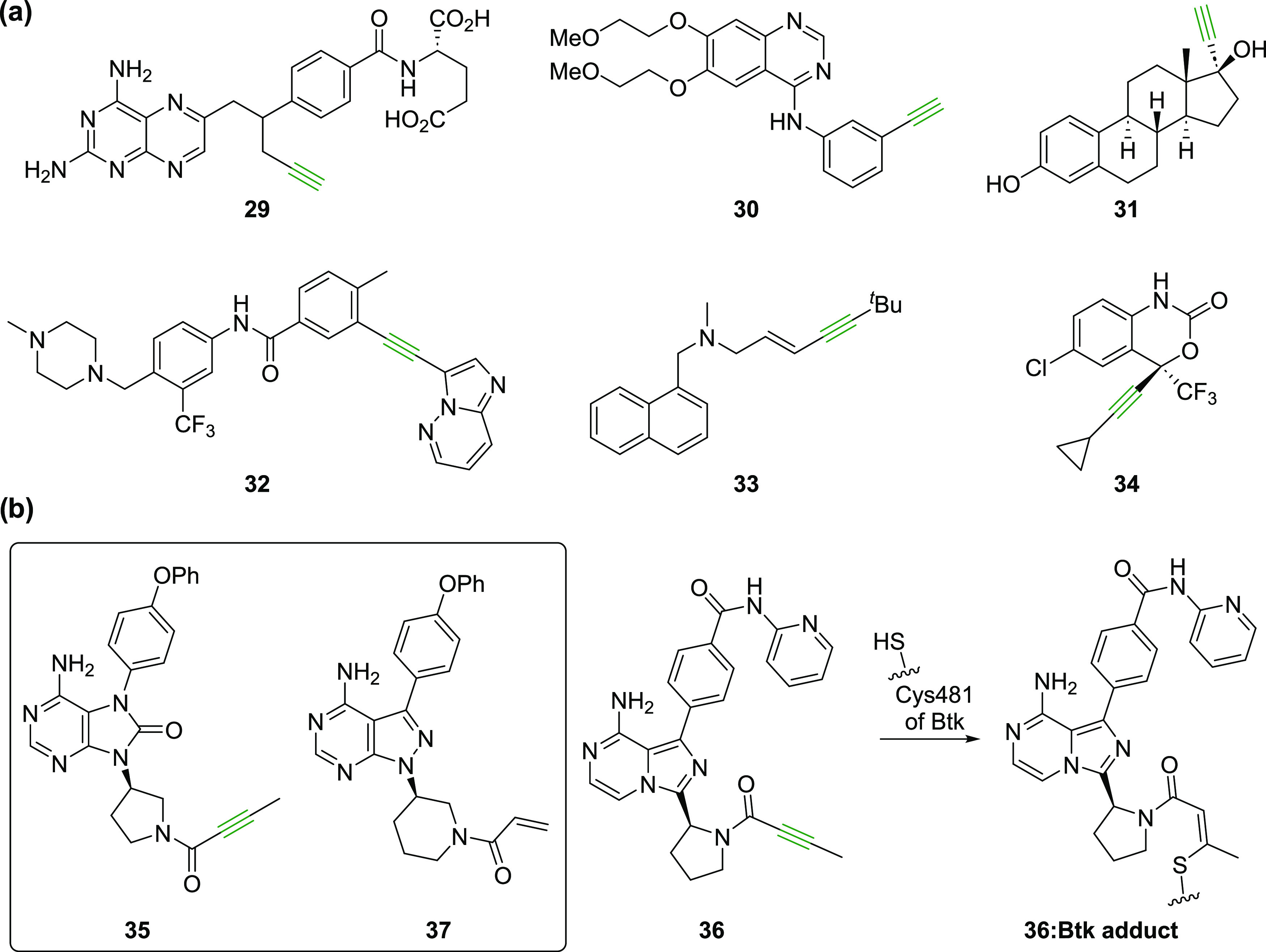
Alkynes are important functional groups in human therapeutics. (a) Representative human therapeutics that contain alkynes (in green); (b) covalent reaction of acalabrutinib (**36**)^87^ with Btk Cys481.

Alkynes are established functional groups in active pharmaceutical ingredients (APIs) of approved human therapeutics (Figure 6a) and molecules under clinical investigation, though they can induce cytotoxicity and they can be metabolized via reaction with CYP450 monooxygenases.^63^ Nonactivated terminal alkynes are present in APIs, e.g., in the dihydrofolate reductase inhibitor pralatrexate (**29**),^78^ in the monoamine oxidase-B inhibitors erlotinib (**30**), selegiline, and rasagiline which are used to alleviate symptoms of Parkinson’s disease,^79^ and in the steroidal drugs danazol, gestrinone, and 17α-ethynylestradiol (**31**), the latter of which is widely used as a contraceptive.^63^ C-Terminal derivatized alkynes occur in APIs of drugs, e.g., in the DPP4 inhibitor linagliptin,^80^ ponatinib (**32**, used to treat acute myeloid leukemia),^81^ the antifungal therapeutic terbinafine (**33**),^82,83^ the HIV reverse transcriptase inhibitor efavirenz (**34**),^84^ the HIV therapeutic lenacapavir,^85^ the steroid mifepristone used for medical abortions,^63^ and in the antibody-drug conjugates gemtuzumab ozogamicin and inotuzumab ozogamicin (used to treat leukemia).^86^

Bruton’s tyrosine kinase (Btk) inhibitors tirabrutinib (**35**) and acalabrutinib (**36**)^87^ (used to treat inter alia chronic lymphocytic leukemia and lymphoma, respectively) contain an yneamide group, the electrophilic alkyne of which covalently reacts with the nucleophilic thiolate of Btk Cys481 resulting in efficient inhibition (Figure 6b),^87,88^ further highlighting the use of alkynes as clinically useful warheads. These two Btk inhibitors were designed based on the structure of the clinically used compound ibrutinib (**37**),^89,90^ which, however, employs an acrylamide group rather than an yneamide group for covalent reactions with Btk Cys481 (Figure 6b).^88^ It has been proposed that off-target Michael reactions of ibrutinib and related acrylamide-bearing Btk inhibitors with other nucleophilic cysteine residues are in part responsible for side effects of ibrutinib and that the corresponding yneamides are less reactive electrophiles with better pharmacokinetic properties.^87,91,92^

Propargyl amides have also been used as (latent) electrophilic warheads for activity-based probes of deubiquitinases, including of SARS-CoV-2 PL^pro^, which covalently reacts via its active site cysteine Cys111 with terminal alkynes.^51,54^ However, nonactivated terminal alkynes have, to our knowledge, not been used as electrophilic warheads in SARS-CoV-2 M^pro^ inhibitors. M^pro^ inhibitors containing terminal alkynes, which are not covalently modified,^93^ and M^pro^ inhibitors with activated alkynes, i.e., yneamides^20^ and bromo alkynes,^53^ have been reported; however, their inhibition mechanisms have not been investigated in detail. We chose to investigate whether nirmatrelvir derivatives with an alkyne electrophilic warhead inhibit SARS-CoV-2 M^pro^ by reaction with Cys145 because (i) nirmatrelvir (**1**) is optimized for tight binding to the M^pro^ active site; hence, its scaffold should favor the reaction with terminal alkynes which are normally less reactive with nucleophiles than nitriles (template effect);^1^ (ii) nirmatrelvir alkyne derivatives may show a different reactivity profile with potential future nirmatrelvir-resistant M^pro^ variants;^94^ (iii) nirmatrelvir alkyne derivatives may show an altered selectivity profile with regard to inhibiting human cysteine proteases; and (iv) nirmatrelvir alkyne derivatives may manifest improved cellular properties, including with respect to penetration and efflux, in the latter case potentially avoiding the need for coadministration with an efflux inhibitor, as done in cellular studies with **1**. Note that unlike GC376 (**3**), which also efficiently inhibits human cathepsins in vitro,^32,95^**1** is reported to be a selective M^pro^ inhibitor, which does not inhibit human proteases;^21^ however, this observation may be refined as human cathepsins may catalyze hydrolysis with similar substrate recognition sites as M^pro^.^96^

Considering that an efficient reaction of alkynes with thiols is proposed to be largely proximity-driven (template effect),^48,50^ it is remarkable that alkyne derivatives of structurally simple scaffolds like the one of GC376 (**3**) undergo relatively efficient reaction with M^pro^ Cys145, implying a broader utility of appropriately complexed nonactivated alkynes to covalently react with nucleophilic cysteine residues and potentially proteins with other nucleophilic residues. Note that a cysteine to serine Btk variant has been reported to be resistant towards treatment with common covalent Btk inhibitors such as ibrutinib, implying that different preorganized binding modes are required for the reaction of alkynes with different types of nucleophiles.^97^ This proposal is consistent with studies showing that the substitution of nucleophilic Cys to Ser, or vice versa, in nucleophilic enzymes normally leads to loss of activity, including in the case of M^pro^.^98,99^

Our studies reveal that both activated and non-activated alkynes are suitable irreversibly reacting warheads to covalently inhibit isolated M^pro^ and SARS-CoV-2 progression in infected cells (Tables 1–3). Considering that alkynes are versatile functional groups which can easily be modified, including at a late stage using mild protocols which include, but are not limited to, trifluoromethylation and Sonogashira couplings as described here (Scheme 3), the studies suggest that there is potential to further optimize nirmatrelvir and related M^pro^ inhibitors, e.g., by introducing substituents on the alkyne, which bind tightly in the M^pro^ S′ sites and/or modulate pharmacodynamic and/or pharmacokinetic properties. The irreversible nature of the reaction of the alkynes compared to the analogous nitriles means that the alkynes should be useful to inform on the selectivity of the analogous nitriles in terms of reactivity in cells, using activity-based profiling-type approaches.^100^

Importantly, our results show that the CF_3_-capped alkyne **28** inhibits SARS-CoV-2 progression in infected VeroE6 cells ∼5-fold more efficiently than the terminal alkyne **13** (EC_50_ ∼ 5.1 μM, Table 3, entry iv), highlighting the potential of appropriately capped alkynes for potent inhibition of isolated SARS-CoV-2 M^pro^ and SARS-CoV-2 progression in cells (and by analogy other nucleophilic cysteine enzymes). Likely, alkynes **13** and **28** can be optimized to achieve similar or better inhibitory activity in cells than nirmatrelvir (**1**) and GC376 (**3**).

Crystallographic analysis reveals that the terminal methylene group of the vinyl thioether resulting from the reaction of **13** with Cys145 is not located in the oxyanion hole, which is formed by the NH groups of Gly143, Ser144, and Cys145, contrasting with the thioimidate nitrogen in the complex formed by **1**, which is located in the oxyanion hole (Figure 4). This observation is of interest given that Ser144Ala M^pro^ is implicated in nirmatrelvir resistance and because of the differences in relative potencies observed for some of the nitriles and alkynes versus WT and Ser144Ala M^pro^. Although further work is required, it may be that optimized derivatives of irreversibly binding alkyne-based inhibitors of WT M^pro^ and/or M^pro^ active site variants can be developed that are more efficient than those bearing reversibly binding nitrile or other electrophilic warheads.

Importantly, weobserved that the covalent reaction of M^pro^ with nirmatrelvir alkyne **13** is irreversible or at least substantially less reversible than its covalent reaction with nirmatrelvir (**1**) (Figure 5). This result contrasts with the reported reversibility of the covalent reaction of M^pro^ with **1** (and other nitrile-bearing small-molecule inhibitors with nucleophilic serine or cysteine proteins^32,44,77^) and with the observed conformation of its thioimidate in the M^pro^:**1** complex, which suggests that the negative charge at the thioimidate N-atom is stabilized by interactions with the oxyanion hole, potentially hampering the protonation of the N-atom.^1,24^

The covalent reaction of the Cys145 thiolate with the terminal alkyne of **13** likely results in the initial formation of a vinyl anion (Figure 4a); vinyl anions are typically more basic than thioimidate anions, likely resulting in protonation of the vinyl anion by an NH in the oxyanion hole, by a nearby water, or by an M^pro^ residue with an acidic proton. This process is likely irreversible as the basicity of an oxyanion hole may be insufficient to deprotonate a non-activated alkene which are per se not very acidic, in accord with our MS data (Figure 5) and the observation that the vinyl group in the M^pro^:**13** complex is oriented away from the oxyanion hole (Figure 4). Note that the M^pro^:**13** complex structure was obtained using data from multiple (12) microcrystals at the recently constructed VMXm microfocus/nanofocus beamline at the Diamond Light Source synchrotron, a method that should have general utility.^75,76^

The inhibition results show that the electrophilic nitrile group is preferred over the isoelectronic alkyne group for inhibition of isolated M^pro^ in a manner independent of the inhibitor scaffold, at least with the evaluated scaffolds under the tested conditions (Tables 1–3). Importantly, however, they imply that the increased potency for the nitrile over the alkyne may be substantially less in the cellular context (Table 1). It should be possible to increase the potency of the alkyne M^pro^ inhibitors by optimizing the P1-4 substrate-equivalent groups as well as the terminal alkyne substituent. Further SAR studies are required to validate our observations, but the results highlight the importance of optimizing potency in cells as well as against isolated M^pro^. The relative difference between the inhibition results with isolated M^pro^ and in cells may in part reflect the irreversible reaction of alkyne inhibitors with M^pro^.

## Conclusions

The results reveal the therapeutic potential for the covalent inhibition of M^pro^ and other nucleophilic cysteine proteases by alkynes, which, in contrast to more electrophilic nitriles such as in nirmatrelvir (**1**), react irreversibly and can be functionalized at the alkyne terminal position, properties that can be used to optimize inhibition, including with respect to drug-resistant M^pro^ variants, selectivity/safety, and pharmacokinetics.

## Experimental Section

The syntheses and characterizations of the SARS-CoV-2 M^pro^ inhibitors used in this work are disclosed in the associated Supporting Information. All compounds are ≥95% pure by NMR and HPLC analysis unless stated otherwise; NMR spectra and HPLC traces are shown for all lead compounds in the associated Supporting Information.

### SARS-CoV-2 M^pro^ Inhibition Assays

Solid-phase extraction coupled to mass spectrometry (SPE-MS) SARS-CoV-2 M^pro^ inhibition assays were performed in buffer (20 mM HEPES, pH 7.5, 50 mM NaCl) at 20 °C as reported, using a freshly thawed aliquot of recombinant SARS-CoV-2 M^pro^ (multiple freeze–thaw cycles were avoided). The M^pro^ sequence was based on the Wuhan-Hu-1 genome^101^ (National Center for Biotechnology Information (NCBI) reference sequence: NC_045512.2). Mpro M^pro^ was prepared according to established procedures,^43^ and a 37-mer oligopeptide (ALNDFSNSGSDVLYQPPQTSITSAVLQ/SGFRKMAFPS-NH_2_), which was based on the on the sequence of the N-terminal SARS-CoV-2 M^pro^ self-cleavage site and which was synthesized as a C-terminal amide and purified by GL Biochem (Shanghai) Ltd. (Shanghai, China), was used as a substrate (2.0 μM).^62^ Note that SPE-MS assays were performed using a lower protein concentration than that reported (0.05 μM rather than 0.15 μM) and in the presence of the N-terminally acetylated C-terminal product peptide (Ac-SGFRKMAFPS-NH_2_) as an internal standard to account for inhibitor-induced suppression of ionization of the product peptide.

In detail, solutions of the inhibitors (100% DMSO) were dry-dispensed across 384-well polypropylene assay plates (Greiner) in an approximately threefold and 11-point dilution series (100 μM top concentration) using an ECHO 550 acoustic dispenser (Labcyte). DMSO and formic acid were used as negative and positive inhibition controls, respectively. The final DMSO concentration was kept constant at 0.5%_v/v_ throughout all experiments (using the DMSO backfill option of the acoustic dispenser). Each reaction was performed in technical duplicates in adjacent wells of the assay plates, and assays were performed in at least two independent duplicates.

An enzyme mixture (25 μL/well), containing freshly thawed SARS-CoV-2 M^pro^ (0.1 μM) in buffer (20 mM HEPES, pH 7.5, 50 mM NaCl), was dispensed across the inhibitor-containing 384-well assay plates with a multidrop dispenser (Thermo Fisher Scientific) at 20 °C under an ambient atmosphere. The plates were subsequently centrifuged (1000 rpm, 5 s) and incubated for 15 min at 20 °C. A substrate mixture (25 μL/well), containing ALNDFSNSGSDVLYQPPQTSITSAVLQ/SGFRKMAFPS-NH_2_ (4.0 μM) and Ac-SGFRKMAFPS-NH_2_ (0.8 μM) in buffer (20 mM HEPES, pH 7.5, 50 mM NaCl), was added using the multidrop dispenser. The plates were centrifuged (1000 rpm, 5 s), and after incubating for 30 min, the reaction was stopped by addition of 10%_v/v_ aqueous formic acid (5 μL/well). The plates were then centrifuged (1000 rpm, 30 s) and analyzed by MS.

Note that Ser144Ala M^pro^ inhibition assays were performed similarly to those for WT M^pro^, however, using double the concentration of Ser144Ala M^pro^ (0.1 μM final assay concentration) compared to WT M^pro^ (0.05 μM final assay concentration). The Ser144Ala mutation was introduced into the plasmid DNA encoding for WT SARS-CoV-2 M^pro^ using standard protocols, and recombinant Ser144Ala M^pro^ was produced and purified as described for WT M^pro^.^43^

MS analyses were performed using a RapidFire RF 365 high-throughput sampling robot (Agilent) attached to an iFunnel Agilent 6550 accurate mass quadrupole time-of-flight (Q-TOF) mass spectrometer operated in the positive ionization mode as previously reported.^43,62^ For data analysis, the m/z +1 charge states of the C-terminal product peptide (SGFRKMAFPS-NH_2_) and the N-terminally acetylated C-terminal product peptide (Ac-SGFRKMAFPS-NH_2_) were used to extract and integrate ion chromatogram data using RapidFire Integrator software (Agilent). Data were exported into Microsoft Excel and used to calculate the product concentration using the following equation: product concentration = 0.4 μM × (integral C-terminal product peptide)/(integral N-terminally acetylated C-terminal product peptide). Normalized dose–response curves (using the formic acid and DMSO controls) were obtained from the raw data by nonlinear regression (GraphPad Prism 5) and used to determine IC_50_ values.

### Protein-Observed M^pro^ Assays

Assays were performed as described using SPE-MS.^62^ Solutions of the inhibitors (100% DMSO) were dry-dispensed across 384-well polypropylene assay plates (Greiner) using an ECHO 550 acoustic dispenser (Labcyte). DMSO was used as a negative control. An enzyme mixture (50 μL/well), containing M^pro^ (2.0 μM) in buffer (20 mM HEPES, pH 7.5), was dispensed across the inhibitor-containing 384-well assay plates with a multidrop dispenser (Thermo Fisher Scientific). The reaction mixture was incubated for the indicated time at 20 °C under an ambient atmosphere prior to analysis by SPE-MS. MS analyses were performed using a RapidFire RF 365 high-throughput sampling robot (Agilent) attached to an iFunnel Agilent 6550 accurate mass Q-TOF mass spectrometer using a C4 cartridge and the same parameters as described.^43,62^

### Cell Viability Assays

Inhibitor toxicity was assayed using the 3-(4,5-dimethylthiazol-2-yl)-2,5-diphenyltetrazolium bromide (MTT) cell proliferation assay kit (Abcam – Ab211091) as per the manufacturers’ recommendation. Compounds were dispensed into 96-well plates using an ECHO 550 acoustic dispenser (Labcyte) to give a final concentration range of 100–0.11 μM. All assays were carried out in technical duplicates. VeroE6 cells (4.5 × 10^4^) were added to each well, and the 96-well plate was then incubated for 24 h at 37 °C in a 5% CO_2_ atmosphere. Following incubation, the media were removed, the MTT reagent was added, and the cells were incubated for 3 h at 37 °C. Subsequently, all media were removed from the cells, the MTT solvent was added to each well, and the plate was incubated on a shaker at rt for 15 min protected from light. The absorbance was measured at 600 nm. OD_600_ values of the different concentrations of compound were divided by the OD_600_ value of the negative control to calculate the percentage cytotoxicity; the cytotoxicity curves were plotted using GraphPad Prism.

### Cell-Based Antiviral Assays

Compounds were dispensed into 96-well plates using an ECHO 550 acoustic dispenser (Labcyte) to give a final concentration range of 100–0.11 μM. Compounds were preincubated with 50 μL of SARS-CoV-2 virus (Victoria strain–100 FFU) and 50 μL of VeroE6 cells (9 × 10^5^/mL) in separate 96-well plates. Following incubation for 1 h at 37 °C in a 5% CO_2_ atmosphere, the virus was added to the well containing compound-treated cells. The virus was allowed to infect the cells for 2 h at 37 °C in a 5% CO_2_ atmosphere, followed by the addition of 100 μL of compound-adulterated carboxymethyl cellulose (1.5%) to each well. Subsequently, the plates were incubated for a further 20 h at 37 °C in a 5% CO_2_ atmosphere. All assays were carried out in technical duplicates.

Cells were washed with 200 μL of DPBS and then fixed with paraformaldehyde 4%_v/v_ (100 μL/well) for 30 min at rt. Cells were permeabilized with TritonX100 (1% in PBS) and then stained for SARS-CoV-2 nucleoprotein using a human monoclonal antibody (FB9B^102^). Bound antibodies were detected following incubation with a goat anti-human IgG HRP conjugate (Sigma, U.K.) and following TrueBlue Peroxidase substrate (Insight Biotechnology, U.K.) addition imaged using an ELISPOT reader. The half-maximal effective concentration (EC_50_) was defined as the concentration of the compound that reduced the Foci forming unit (FFU) by 50% compared to the control wells.

### Crystallization

A frozen SARS-CoV-2 M^pro^ solution was thawed and diluted to 6 mg/mL (using 20 mM HEPES, pH 7.5, 50 mM NaCl). Alkyne **13** was added to the protein solution to a final concentration of 10 mM; the mixture was incubated for 6 h at ambient temperature prior to dispensing plates. The drop composition was 0.15 μL of protein ligand solution, 0.3 μL of 11%_v/v_ PEG 4000, 0.1 M MES, pH 6.5, and 0.05 μL of M^pro^ crystal seed stock. The M^pro^ crystal seed stock was prepared by crushing M^pro^ crystals with a pipette tip, suspending them in 30% PEG 4000, 5%_v/v_ DMSO, 0.1 M MES, pH 6.5, and vortexing for 30 s with approximately 10 glass beads (1.0 mm diameter, BioSpec products). The reservoir solution contained: 11%_v/v_ PEG 4K, 5%_v/v_ DMSO, 0.1 M MES, pH 6.5. Note that DMSO was added to the reservoir solution; but in the crystallization drop, DMSO is provided by the solution of the ligand incubated with the protein. Crystals were grown using the sitting drop vapor diffusion method at 20 °C and appeared within 24 h. Crystal morphology was needle-like with a diameter of approximately 1 μm.

### Data Collection and Structure Determination

Crystals were applied to an electron microscopy grid as described.^75^ In brief, standard holey carbon grids (Quantifoil, Cu 200 mesh, R2/2) were glow-discharged for 30 s at 15 mA. Crystals in a drop were resuspended in 10 μL of reservoir solution, and 3 μL of the resulting suspension was applied to the grid surface in a Leica EM GP2 plunge-freezing device at >80% humidity. Then, 2 μL of reservoir solution was dispensed onto the rear face of the grid. The grid was blotted from the back for 10 s prior to plunging into liquified ethane.

Diffraction data were collected at 100 K and a wavelength of 0.5813 Å at the VMXm beamline at Diamond Light Source from 24 crystals. 40° sweeps were collected from each crystal using 0.1° oscillations. The data were collected on an Eiger 9M CdTe detector. Data were processed using Dials^103^ via xia2.multiplex;^76,104^ data from 12 crystals were present in the final merged dataset. The datasets were phased using Molrep^105^ and the M^pro^ apo structure (PDB ID: 6YB7). Ligand restraints were generated using AceDRG;^106^ 96.0% of the residues are in the favored regions of the Ramachandran plot, 3.3% in the allowed region, and 0.7% in high-energy conformations (two residues). Crystal structures were manually rebuilt in Coot and refined using Refmac^107^ and PDB_Redo (Supporting Table S4).^108^

The crystal structure data for the SARS-CoV-2 M^pro^:**13** complex structure have been deposited in the protein data bank (PDB) with accession code 8B2T. Additionally, data of the following reported crystal structure have been used: 7TE0.^22^

## Supporting Information Available

Mass spectrometry assays; assay standards; inhibition assays; small-molecule synthesis; NMR and HPLC analyses, crystallographic analysis; general synthetic procedures; experimental procedures (PDF)
Molecular formula strings (CSV)

## Abbreviations Used

API: active pharmaceutical ingredient
COVID-19: coronavirus disease 2019
Btk: Bruton’s tyrosine kinase
DUB: deubiquitinase
HCV: hepatitis C virus
HIV: human immunodeficiency virus
M^pro^: main protease
PL^pro^: papain-like protease
SARS-CoV-2: severe acute respiratory syndrome coronavirus 2
