# Supplementary material for: Alkyne Derivatives of SARS-CoV-2 Main Protease Inhibitors Including Nirmatrelvir Inhibit by Reacting Covalently with the Nucleophilic Cysteine
Source: J Med Chem. 2023 Feb 9;66(4):2663–80. doi: 10.1021/acs.jmedchem.2c01627 (PMC9924091; doi:10.1021/acs.jmedchem.2c01627)
Supplement: Supplementary file 1 — jm2c01627_si_001.pdf [file jm2c01627_si_001.pdf]

Supporting Information

Alkyne derivatives of SARS-CoV-2 main protease inhibitors including nirmatrelvir inhibit by reacting covalently with the nucleophilic cysteine

Lennart Brewitz[1,#,*], Leo Dumjahn[1,#], Yilin Zhao[1,#], C. David Owen[2,3], Stephen M. Laidlaw[4], Tika R. Malla[1], Dung Nguyen[4], Petra Lukacik[2,3], Eidarus Salah[1], Adam D. Crawshaw[2], Anna J. Warren[2], Jose Trincao[2], Claire Strain-Damerell[2,3], Miles W. Carroll[4], Martin A. Walsh[2,3], and Christopher J. Schofield[1,*]

[1]Chemistry Research Laboratory, Department of Chemistry and the Ineos Oxford Institute for Antimicrobial Research, University of Oxford, 12 Mansfield Road, OX1 3TA, Oxford, United Kingdom.

[2]Diamond Light Source Ltd., Harwell Science and Innovation Campus, OX11 0DE, Didcot, United Kingdom.

[3]Research Complex at Harwell, Harwell Science and Innovation Campus, OX11 0FA, Didcot, United Kingdom.

[4]Wellcome Centre for Human Genetics, Nuffield Department of Medicine, University of Oxford, OX3 7BN, Oxford, United Kingdom.

[#]These authors contributed equally to this work.

*Email: christopher.schofield@chem.ox.ac.uk or lennart.brewitz@chem.ox.ac.uk

Table of contents

1. Supporting Figures S2-S24
2. Supporting Tables S25-S30
3. General synthesis information S31
4. General synthetic procedures S32-S33
5. Experimental procedures and compound characterizations S34-S43
6. References S44-S45
7. ^{1}H and ^{13}C NMR spectra of novel alkyne-containing SARS-CoV-2 M^{pro} inhibitors prepared for this study S46-S53
8. HPLC traces of selected SARS-CoV-2 M^{pro} inhibitors prepared for this study S54-S58

1. Supporting Figures

Supporting Figure S1. Synthesis and purification of the N-terminally acetylated 37mer substrate-derived C-terminal product peptide. (**a**) Sequence and purification characteristics of the N-terminally acetylated C-terminal product peptide derived from the 37mer M^{pro} substrate (ALNDFSNSGSDVLYQPPQTSITSAVLQ/SGFRKMAFPS-NH_2; '/' indicates the cleavage site)[1-2], that is: Ac-SGFRKMAFPS-NH_2. The Ac-SGFRKMAFPS-NH_2 peptide was synthesized as a C-terminal amide using microwave-assisted solid phase peptide synthesis (SPPS) (from the C- to N-terminus). After complete assembly of the protected peptide sequence, the Fmoc group of the N-terminal amino acid residue was cleaved using 20%$_{v/v}$ piperidine in DMF. The resultant free N-terminal amine was capped with an acetyl group using commercially-sourced *N*-acetoxysuccinimide (Tokyo Chemical Industry UK Ltd.) in DMF according to a reported procedure.[3] The N-terminally acetylated peptide was cleaved from the resin and simultaneously deprotected, then purified using a semi-preparative HPLC machine (Shimadzu UK Ltd.) equipped with a reverse phase column (Gemini 00G-4454-U0-AX; phase: NX-C18). The purity (>90%) and mass of the Ac-SGFRKMAFPS-NH_2 peptide was determined using HPLC and solid phase extraction coupled to mass spectrometry (SPE-MS), respectively; (**b**) mass spectrum (SPE-MS) of the Ac-SGFRKMAFPS-NH_2 peptide. m/z = 1068.55 and 1190.53 correspond to the +1 charge state of the Ac-SGFRKMAFPS-NH_2 peptide, the latter observed as the Na^+ ion; the enlarged region shows the m/z +1 peaks. m/z = 584.78 corresponds to the +2 charge state of the Ac-SGFRKMAFPS-NH_2 peptide. Note, m/z values refer to the most abundant isotope.

(a) *N*-Acetylated C-terminal hydrolysis product of the 37mer SARS-CoV-2 M^{pro} substrate peptide

Ac-S-G-F-R-K-M-A-F-P-S-NH_2

HPLC gradient: 2%$_{v/v}$ to 45%$_{v/v}$ MeCN/H_2O (+0.1%$_{v/v}$ TFA) over 37 min
t_R: 20.4 min
SPE-MS: m/z calculated for $C_{53}H_{82}N_{15}O_{13}S$ $[M+H]^+$: 1168.5932, found: 1168.5492

(b)

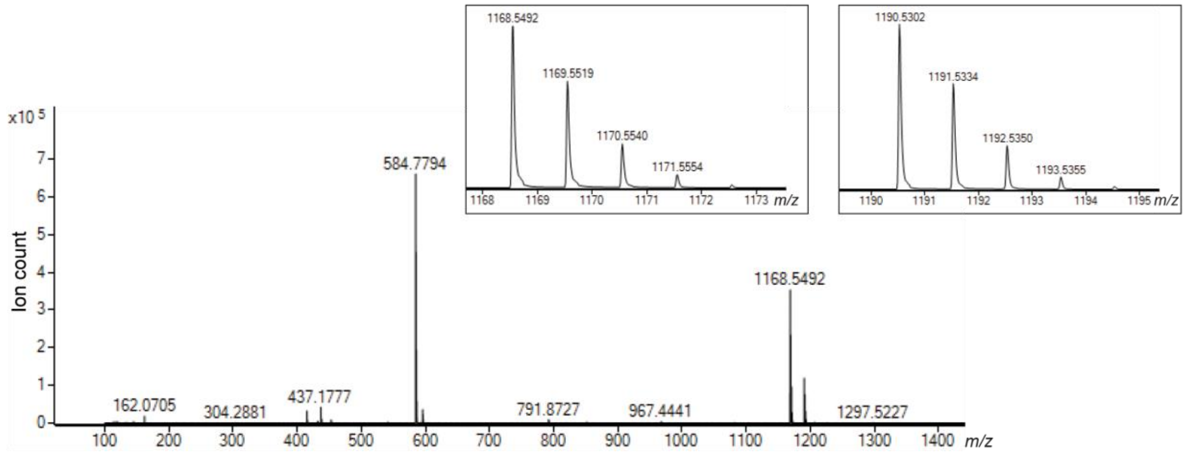

Supporting Figure S2. The use of the N-terminally acetylated 37mer substrate-derived C-terminal product peptide as an internal standard does not affect SARS-CoV-2 M^{pro} catalysis. (**a**) The N-terminally acetylated C-terminal product peptide (*i.e.* Ac-SGFRKMAFPS-NH_2; black circles) derived from the used 37mer SARS-CoV-2 M^{pro} substrate ALNDFSNSGSDVLYQPPQTSITSAVLQSGFRKMAFPS-NH_2[1-2] is not a substrate for isolated recombinant SARS-CoV-2 M^{pro}, as the corresponding deacylation product (orange diamonds) was not observed by SPE-MS under the assay conditions (conditions: 0.1 µM M^{pro} and 2.0 µM Ac-SGFRKMAFPS-NH_2 in 20 mM HEPES, pH 7.5, 50 mM NaCl at ambient temperature). Results are a mean of independent triplicates (n = 3; mean ± standard deviation, SD). Note that the corresponding 37mer-derived N-terminally acetylated N-terminal product peptide is not suitable as an internal standard due to its comparatively poor ionization under the assay conditions; (**b**) The presence of the N-terminally acetylated C-terminal product peptide Ac-SGFRKMAFPS-NH_2 from the used 37mer SARS-CoV-2 M^{pro} substrate (ALNDFSNSGSDVLYQPPQTSITSAVLQSGFRKMAFPS-NH_2[1-2]) in the assay mixture does not affect M^{pro} catalysis. SPE-MS analysis reveals that M^{pro} catalyzes substrate depletion with similar rates in the absence (blue circles) and presence (pink diamonds) of the Ac-SGFRKMAFPS-NH_2 peptide (conditions: 0.05 µM M^{pro}, 2.0 µM ALNDFSNSGSDVLYQPPQTSITSAVLQSGFRKMAFPS-NH_2[1-2], with or without 0.2 µM Ac-SGFRKMAFPS-NH_2 in 20 mM HEPES, pH 7.5, 50 mM NaCl at ambient temperature). Results are a mean of independent triplicates (n = 3; mean ± SD).

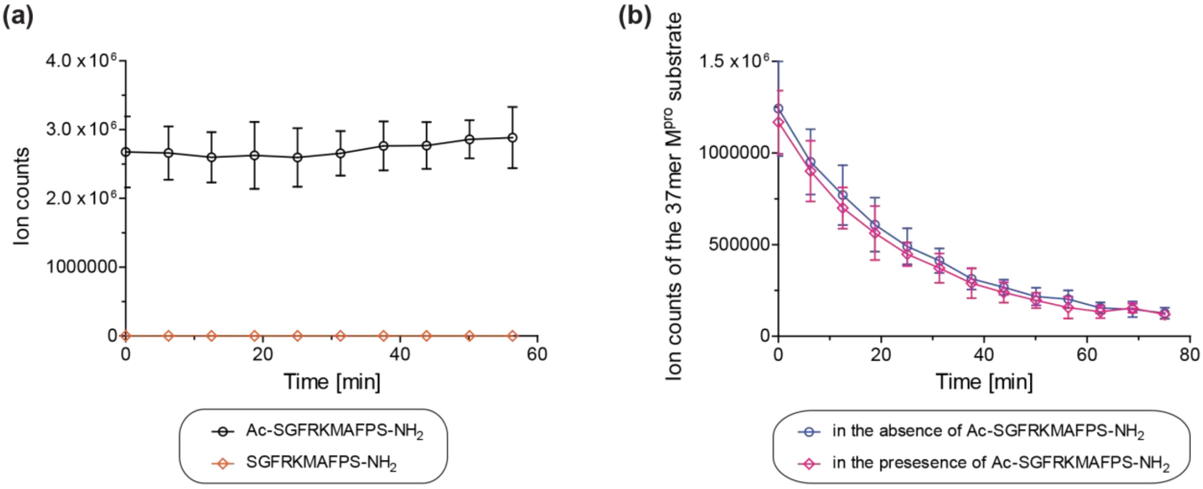

Supporting Figure S3. Dose-responses observed for nitrile- and alkyne-bearing SARS-CoV-2 M^pro^ inhibitors from cell-based experiments used to determine EC_{50} values. Cell-based antiviral assays were performed as described in the Experimental Section. Results are a mean of independent triplicates (n = 3; mean ± SD). Note that dose-response curves are only shown for those inhibitors which were active over the tested concentration range (100 µM top concentration). Dose-response curves of M^{pro} inhibitors shown in (**a**) Table 1, (**b**) Table 2, and (**c**) Table 3.

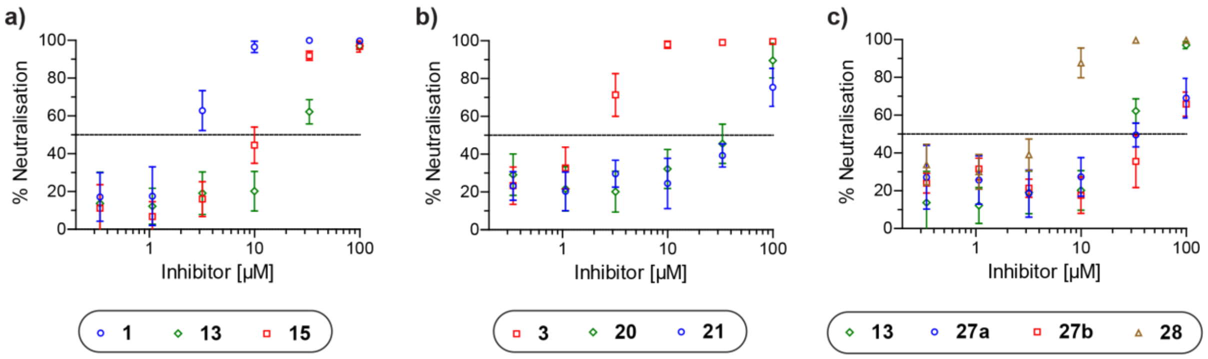

Supporting Figure S4. Synthesis of alkyne and nitrile derivatives of nirmatrelvir with a methionine at the P1 equivalent position. Alkyne **14** was synthesized from commercially-sourced Boc-*L*-methionine (**38**) in five steps using the reported methionine alkyne **40**[4] as an intermediate. Thus, Boc-*L*-methionine (**38**) was converted to the corresponding Weinreb amide[5], which was reduced to aldehyde **39** using $LiAlH_4$. Aldehyde **39** was converted to the corresponding alkyne using the Ohira-Bestmann reagent **10**;[6-7] the product was deprotected with HCl, and coupled to the reported acid **12**[8] using COMU[9] to afford **14** in 17% yield, following HPLC purification. Nirmatrelvir methionine nitrile **15** was obtained in two steps from commercially-sourced *L*-methionine amide (**41**), by adapting Pfizer's synthesis of nirmatrelvir (**1**).[8] The commercially-sourced *L*-methionine amide was coupled with the reported acid **12**[8] using COMU,[9] and the intermediate amide was dehydrated using the Burgess reagent (**42**)[10-11] to afford nitrile **15** in 13% yield, following HPLC purification.

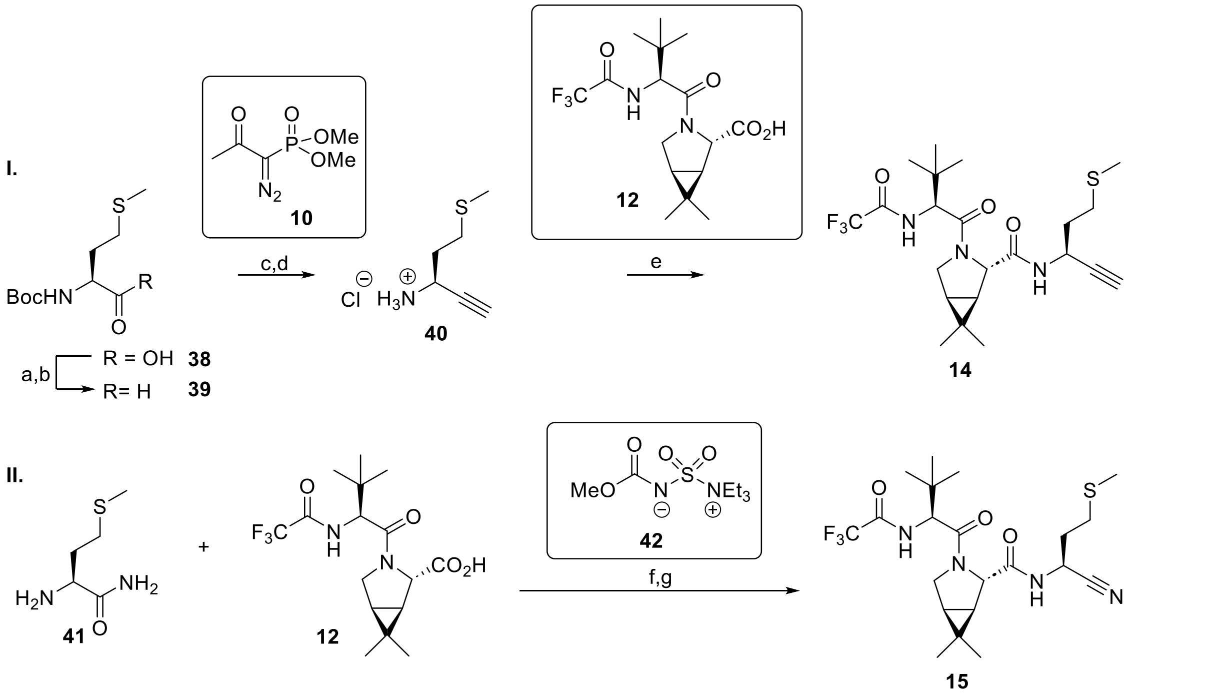

Reagents and conditions: a) Me(OMe)NH•HCl, 1,1'-carbonyldiimidazole (CDI), $^{i}PrNEt_2$, CH_2Cl_2, rt, 99%; b) $LiAlH_4$, THF/Et_2O, 0 °C, 94%; c) **10**[6-7], K_2CO_3, MeOH, rt, 47%; d) HCl (4 M in dioxane), CH_2Cl_2, rt, app. quant.; e) **12**[8], COMU[9], *N*-methylmorpholine (NMM), DMF/CH_2Cl_2, 0 °C to rt, 17%; f) **12**[8], COMU[9], NMM, DMF/CH_2Cl_2, 0 °C to rt, 36%; g) **42**[10-11], CH_2Cl_2, rt, 13%.

Supporting Figure S5. Robustness of the SARS-CoV-2 M^pro SPE-MS inhibition assays. (**a**) Z'-factors[12] (circles) and (**b**) signal-to-noise ratios (S/N, boxes) for all SARS-CoV-2 M^pro inhibition assay plates analyzed to determine IC_{50}-values. The Z'-factors >0.5 (grey line) indicate a stable and robust assay of high quality.[12] Z'-factors and S/N-ratios were determined according to the literature using Microsoft Excel.[12]

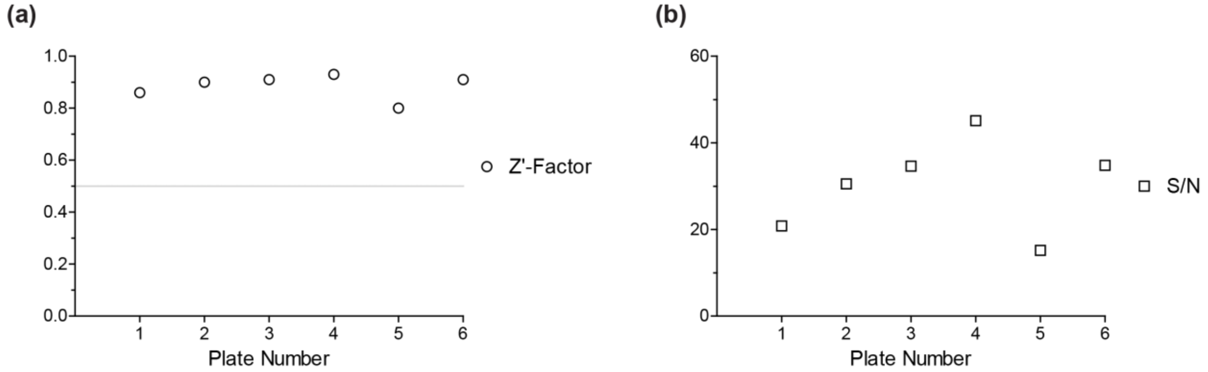

Supporting Figure S6. Synthesis of the MI-09 derived nitriles 20 and 21. The nitrile-bearing MI-09 (**4**)[13] derivatives **20** and **21** were synthesized according to procedures for the synthesis of nirmatrelvir (**1**).[8] The reported acids **16**[13] or **17** were coupled with the reported amide **43**[8] using COMU.[9] The intermediate amides were dehydrated using the Burgess reagent (**42**)[10-11] to afford nitriles **20** and **21** in 53% and 58% yield, respectively, following HPLC purification.

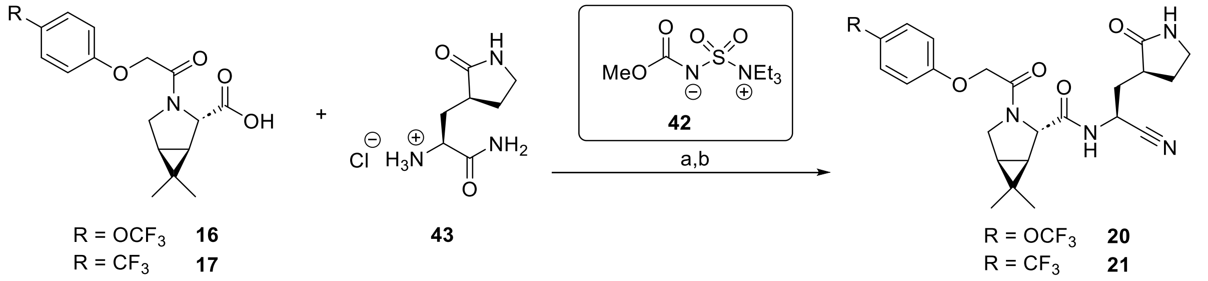

Reagents and conditions: a) **16**[13] or **17**, COMU[9], *N*-methylmorpholine (NMM), DMF/CH_2Cl_2, 0 °C to rt, 17% and 41%, respectively; b) **42**[10-11], CH_2Cl_2, rt, 53% and 58%, respectively.

Supporting Figure S7. Crystallographic analysis of the M^{pro}:13 complex. Color code: SARS-CoV-2 M^{pro}: grey; carbon-backbone of **13** in complex with M^{pro} is in orange; oxygen: red; nitrogen: blue; sulfur: yellow; fluorine: light blue. (**a**) Overview of the SARS-CoV-2 M^{pro}:**13** complex structure; (**b**) superimposition of a view from the M^{pro}:**13** complex structure with one from the reported M^{pro}:**1** complex structure (pale yellow: M^{pro}; green: carbon-backbone of nirmatrelvir (**1**) in complex with M^{pro}; PDB ID: 7TE0[14]) reveals similar M^{pro} conformations (RMSD = 0.313 Å).

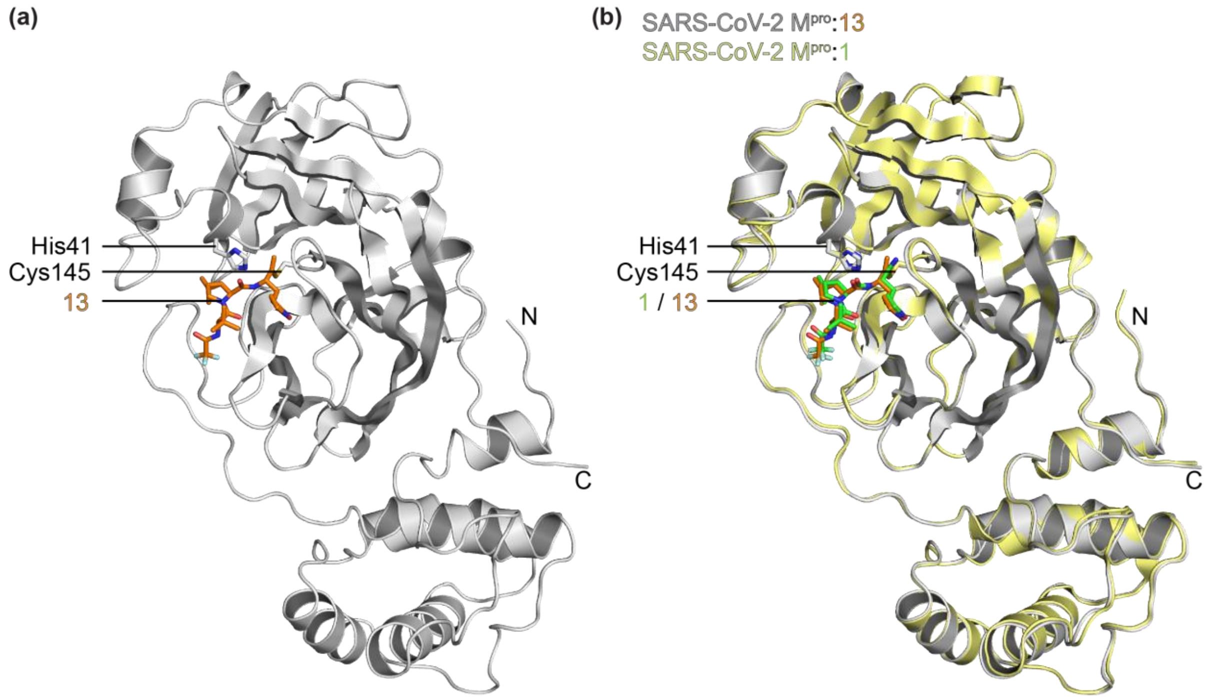

Supporting Figure S8. Nirmatrelvir (1) and nirmatrelvir-derived alkyne 13 bind with SARS-CoV-2 M^{pro} in a similar overall manner (continues on the following page). Color code: SARS-CoV-2 M^{pro}: grey; carbon-backbone of **13** in complex with M^{pro} is in orange; oxygen: red; nitrogen: blue; sulfur: yellow; fluorine: light blue. (**a** and **b**) View of the surface from the M^{pro}:**13** complex structure active site (**a**) revealing that the groups at the P1-4 substrate-equivalent positions of **13** occupy the same M^{pro} substrate binding pockets as reported for an M^{pro}:**1** complex structure (**b**, pale yellow: M^{pro}; green: carbon-backbone of nirmatrelvir (**1**) in complex with M^{pro}; PDB ID: 7TE0[14]), *i.e.* the cycloglutamine groups occupy the S1 pocket, the bicyclic leucine isosteres occupy the hydrophobic S2 pocket, the *tert*-butyl groups are solvent exposed, and the trifluoroacetamide groups occupy the S4 pocket. (**c**) Alkyne **13** in complex with M^{pro} is covalently bound to Cys145 as a thioether (distance of the Cys145 S-atom to the internal C-atom of the vinyl group: 1.8 Å). The trifluoroacetamide NH of **13** is positioned to interact with the main chain carbonyl O-atom of Glu166 (2.8 Å), the carbonyl O-atom of the *tert*-butylglycine unit of **13** is positioned to interact with the main chain amide NH of Glu166 (2.9 Å), the amide NH linking the bicyclic leucine isostere and the cycloglutamine unit of **13** is positioned to interact with the main chain amide carbonyl O-atom of His164 (3.0 Å), and the carbonyl O-atom of the cycloglutamine side chain of **13** is positioned to interact with an imidazole NH of the His163 side chain (2.7 Å). (**d**) Nirmatrelvir (**1**) in complex with M^{pro} (pale yellow: M^{pro}; green: carbon-backbone of **1** in complex with M^{pro}; PDB ID: 7TE0[14]) is covalently bound to Cys145 via a thioimidate (distance of the Cys145 S-atom to the thioimidate C-atom: 1.8 Å). The trifluoroacetamide NH of **1** is positioned to interact with the main chain carbonyl O-atom of Glu166 (2.8 Å), the carbonyl O-atom of the *tert*-butylglycine unit of **1** is positioned to interact with the main chain amide NH of Glu166 (2.8 Å), the amide NH linking the bicyclic leucine isostere and the cycloglutamine unit of **1** is positioned to interact with the main chain amide carbonyl O-atom of His164 (2.9 Å), and the carbonyl O-atom of the cycloglutamine side chain of **1** is positioned to interact with an imidazole NH of the His163 side chain (2.6 Å). The thioimidate N-atom of **1** is positioned to interact with the main chain NHs of Cys145 and Gly143 which form the oxyanion hole (distances of the thioimidate N-atom to the Cys145 and Gly143 main chain NHs: 3.1 and 3.3 Å, respectively).

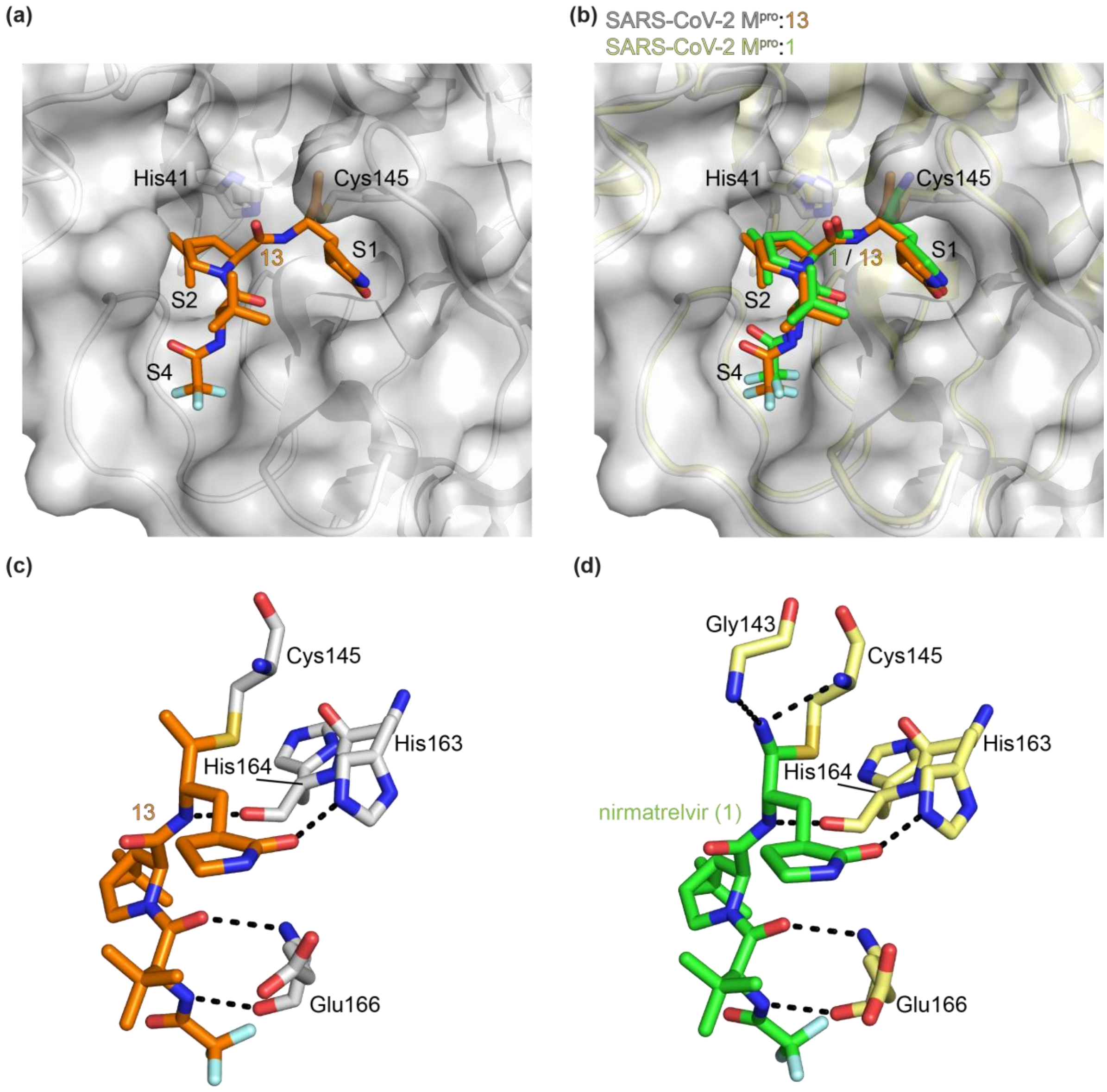
(a)
His41
Cys145
13
S1
S2
S4
(b)
SARS-CoV-2 Mpro:13
SARS-CoV-2 Mpro:1
His41
Cys145
1 / 13
S1
S2
S4
(c)
Cys145
His163
His164
13
Glu166
(d)
Gly143
Cys145
His163
His164
nirmatrelvir (1)
Glu166

Supporting Figure S9. Crystallographic analysis reveals that vinyl groups of vinyl thioethers formed with M^{pro} Cys145 can occupy different conformations in/adjacent to the oxyanion hole (continues on the following page). Color code: SARS-CoV-2 M^{pro}: grey; carbon-backbone of **13** in complex with M^{pro} is in orange; oxygen: red; nitrogen: blue; sulfur: yellow; fluorine: light blue. (**a**) Superimposition of a view from the M^{pro}:**13** complex structure with one from the reported M^{pro}:**44** complex structure (pale green: M^{pro}; slate blue: carbon-backbone of **44** in complex with M^{pro}; PDB ID: 5RG2[15]) reveals similar M^{pro} conformations (RMSD = 0.503 Å). Note that the mechanism by which the corresponding bromoalkyne of **44** reacts with Cys145 has not been investigated; however, **44** may be a relatively non-specific inhibitor. (**b**) Superimposition of a view from the M^{pro}:**13** complex structure with one from the reported M^{pro}:**45** complex structure (pale pink: M^{pro}; yellow: carbon-backbone of **45** in complex with M^{pro}; PDB ID: 5RG3[15]) reveals overall similar M^{pro} conformations (RMSD = 0.503 Å). Note that the mechanism by which the corresponding bromoalkyne of **45** reacts with Cys145 has not been investigated; however, **45** may be a relatively non-specific inhibitor. (**c**) The conformation of **44** in the M^{pro} active site (PDB ID: 5RG2[15]) differs from that of **13** in complex with M^{pro}. **44** is covalently bound to Cys145 as a thioether, its vinyl group faces into the oxyanion hole (distances of the terminal olefin C-atom to the Cys145 and Gly143 main chain NHs: 2.7 and 3.1 Å, respectively); by contrast, the vinyl group of **13** in complex with M^{pro} projects out of the oxyanion hole. This observation may reflect the different binding modes of **13** and **44** with M^{pro}, *i.e.* **44** is positioned to interact with the main chain of Thr26. (**d**) The conformation of **45** in the M^{pro} active site (PDB ID: 5RG3[15]) differs from that of **13** in complex with M^{pro}. **45** is covalently bound to Cys145 as a thioether, its vinyl group faces into the oxyanion hole (distances of the terminal olefin C-atom to the Cys145 and Gly143 main chain NHs: 2.9 and 2.9 Å, respectively); by contrast, the vinyl group of **13** in complex with M^{pro} projects out of the oxyanion hole. This observation may reflect the different binding modes of **13** and **45** with M^{pro}, *i.e.* **45** is positioned to interact with the main chain of Thr26.

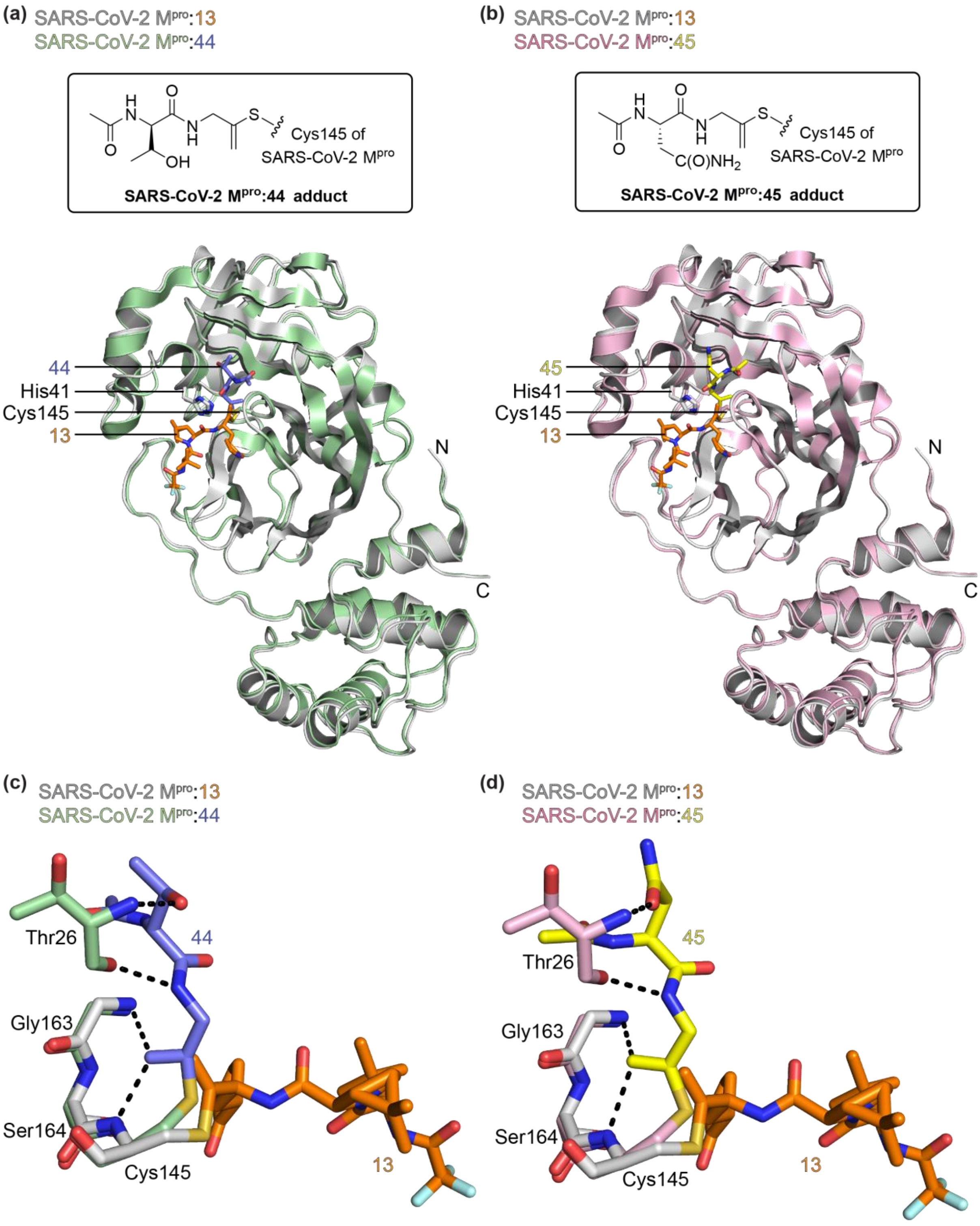
(a)
SARS-CoV-2 Mpro:13
SARS-CoV-2 Mpro:44
Cys145 of SARS-CoV-2 Mpro
SARS-CoV-2 Mpro:44 adduct
44
His41
Cys145
13
N
C
(b)
SARS-CoV-2 Mpro:13
SARS-CoV-2 Mpro:45
C(O)NH2
Cys145 of SARS-CoV-2 Mpro
SARS-CoV-2 Mpro:45 adduct
45
His41
Cys145
13
N
C
(c)
SARS-CoV-2 Mpro:13
SARS-CoV-2 Mpro:44
Thr26
44
Gly163
Ser164
Cys145
13
(d)
SARS-CoV-2 Mpro:13
SARS-CoV-2 Mpro:45
Thr26
45
Gly163
Ser164
Cys145
13

Supporting Figure S10. Mass spectrometric evidence that nirmatrelvir reacts covalently with the nucleophilic Cys145 of SARS-CoV-2 M^{pro}. Protein-observed MS studies were performed as reported using SPE-MS.[1-2] Conditions: SARS-CoV-2 M^{pro} (2.0 μM) was incubated with nirmatrelvir[8] (**1**; 10 μM) in buffer (20 mM HEPES, pH 7.5; 50 μL total reaction volume) for 90 min at ambient temperature, before stopping the reaction by addition of $1\%_{v/v}$ aqueous formic acid (5 μL) and analysis by SPE-MS using reported parameters.[1-2]

Nirmatrelvir (**1**) reacts covalently with SARS-CoV-2 M^{pro} as revealed by the anticipated ~500 Da mass shift with respect to unmodified M^{pro} (33796 Da), an observation which is in accord with crystallographic analysis showing selective reaction with the nucleophilic Cys145 (Figure 1). Note that the minor peak corresponding to a second M^{pro} modification (*i.e.* 34796 Da) may be an artefact due to non-active site binding.

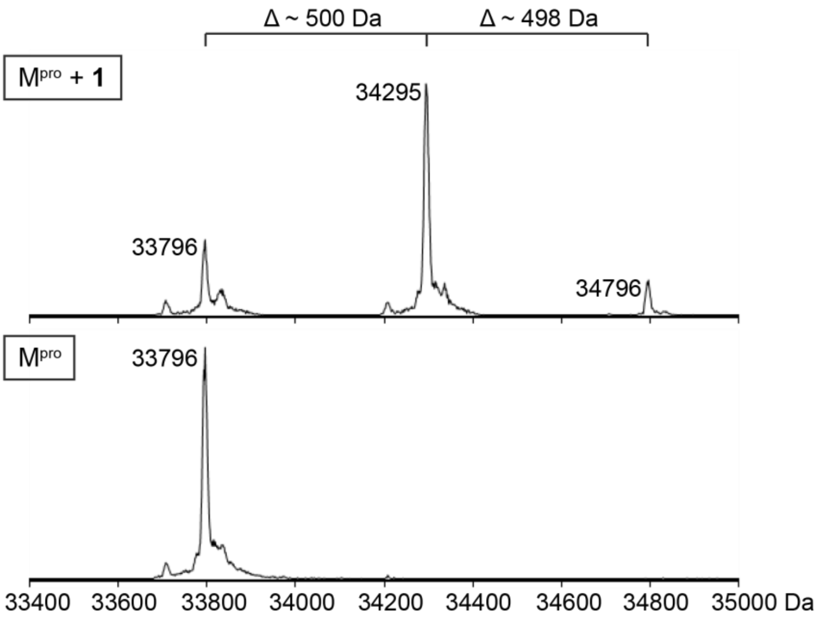

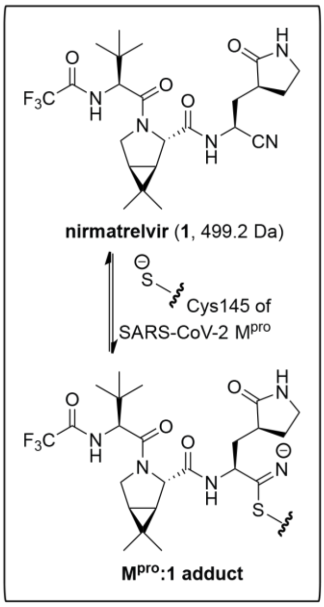

Supporting Figure S11. Mass spectrometric evidence that alkyne 13 reacts covalently with the nucleophilic Cys145 of SARS-CoV-2 Mpro. Protein-observed MS studies were performed as reported using SPE-MS.[1-2] Conditions: SARS-CoV-2 Mpro (2.0 µM) was incubated with nirmatrelvir derivative **13** (10 µM) in buffer (20 mM HEPES, pH 7.5; 50 µL total reaction volume) for 90 min at ambient temperature, before stopping the reaction by addition of 1%$_{v/v}$ aqueous formic acid (5 µL) and analysis by SPE-MS using reported parameters.[1-2]
The alkyne **13** reacts covalently with SARS-CoV-2 Mpro as revealed by the anticipated ~502 Da mass shift with respect to unmodified Mpro (33796 Da), an observation which is in accord with crystallographic analysis showing selective reaction with the nucleophilic Cys145 (Figure 4). Note that the minor peak corresponding to a second Mpro modification (*i.e.* 34793 Da) may be an artefact due to non-active site binding. The results indicate that **13** reacts with Mpro to completion within 90 min.

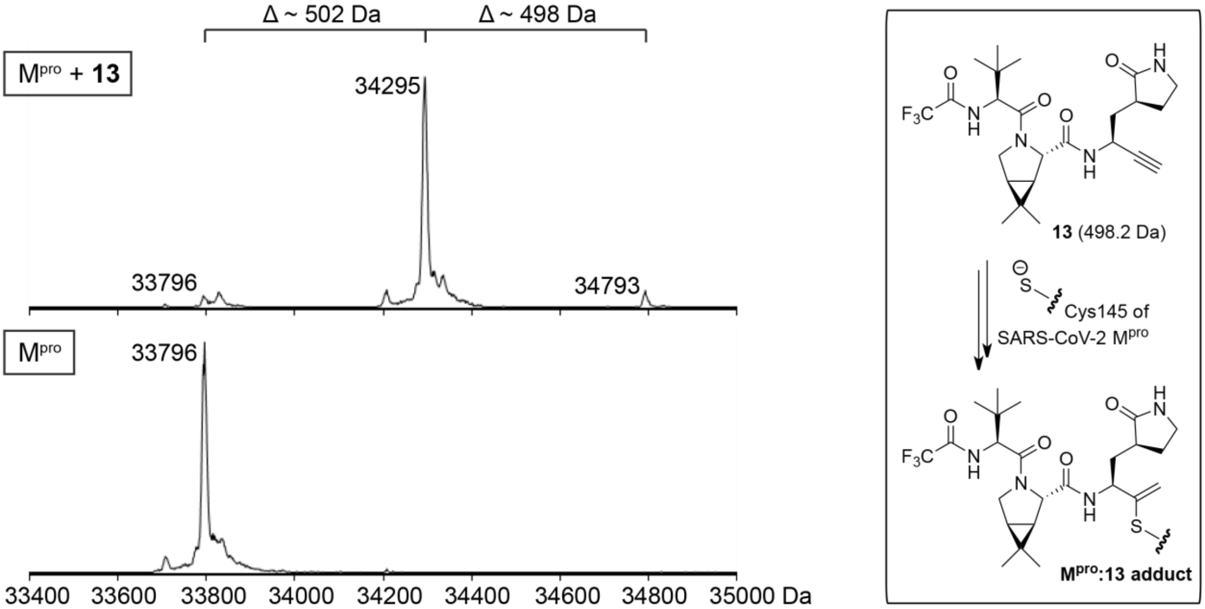

Supporting Figure S12. Mass spectrometric evidence that methionine alkyne 14 reacts covalently with the nucleophilic Cys145 of SARS-CoV-2 M^{pro}. Protein-observed MS studies were performed as reported using SPE-MS.[1-2] Conditions: SARS-CoV-2 M^{pro} (2.0 μM) was incubated with nirmatrelvir derivative **14** (10 μM) in buffer (20 mM HEPES, pH 7.5; 50 μL total reaction volume) for 90 min at ambient temperature, before stopping the reaction by addition of 1%$_{v/v}$ aqueous formic acid (5 μL) and analysis by SPE-MS using reported parameters.[1-2] The methionine alkyne derivative **14** reacts covalently with SARS-CoV-2 M^{pro} as revealed by the anticipated ~475 Da mass shift with respect to unmodified M^{pro} (33796 Da), likely with the nucleophilic Cys145 as crystallographically observed by reaction of alkyne **13** with M^{pro} (Figure 4). Alkyne **14** appears to react less efficiently with M^{pro} than alkyne **13**, in accord with its ~85-fold higher IC$_{50}$ value (Table 1).

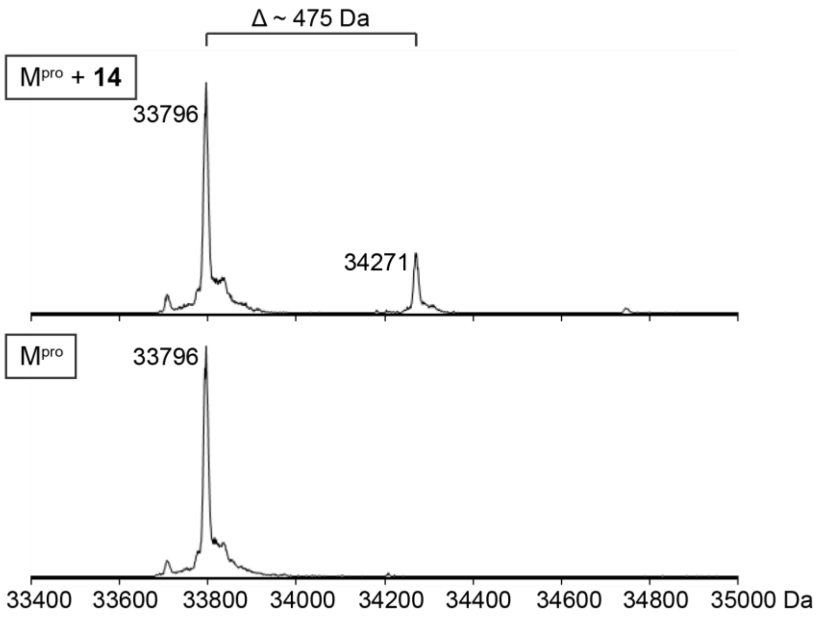

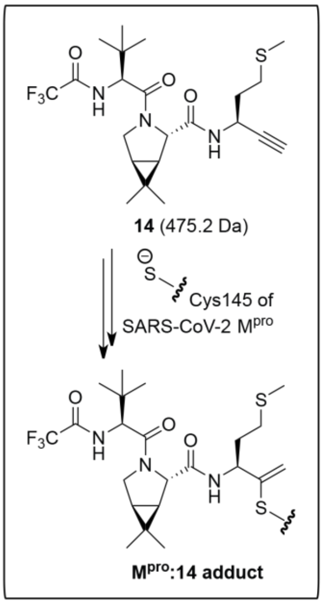

Supporting Figure S13. Mass spectrometric evidence that methionine nitrile 15 reacts covalently with the nucleophilic Cys145 of SARS-CoV-2 M^{pro}. Protein-observed MS studies were performed as reported using SPE-MS.[1-2] Conditions: SARS-CoV-2 M^{pro} (2.0 μM) was incubated with nirmatrelvir derivative **15** (10 μM) in buffer (20 mM HEPES, pH 7.5; 50 μL total reaction volume) for 90 min at ambient temperature, before stopping the reaction by addition of 1%$_{v/v}$ aqueous formic acid (5 μL) and analysis by SPE-MS using reported parameters.[1-2] The methionine nitrile **15** reacts covalently with SARS-CoV-2 M^{pro} as revealed by the anticipated ~476 Da mass shift with respect to unmodified M^{pro} (33796 Da), likely with the nucleophilic Cys145 as crystallographically observed by reaction of **1** with M^{pro} (Figure 1). Nitrile **15** appears to react less efficiently with M^{pro} than nitrile **1**, in accord with its ~35-fold higher IC_{50} value (Table 1). Note that the minor peak corresponding to a second M^{pro} modification (*i.e.* 34796 Da) may be an artefact due to non-active site binding.

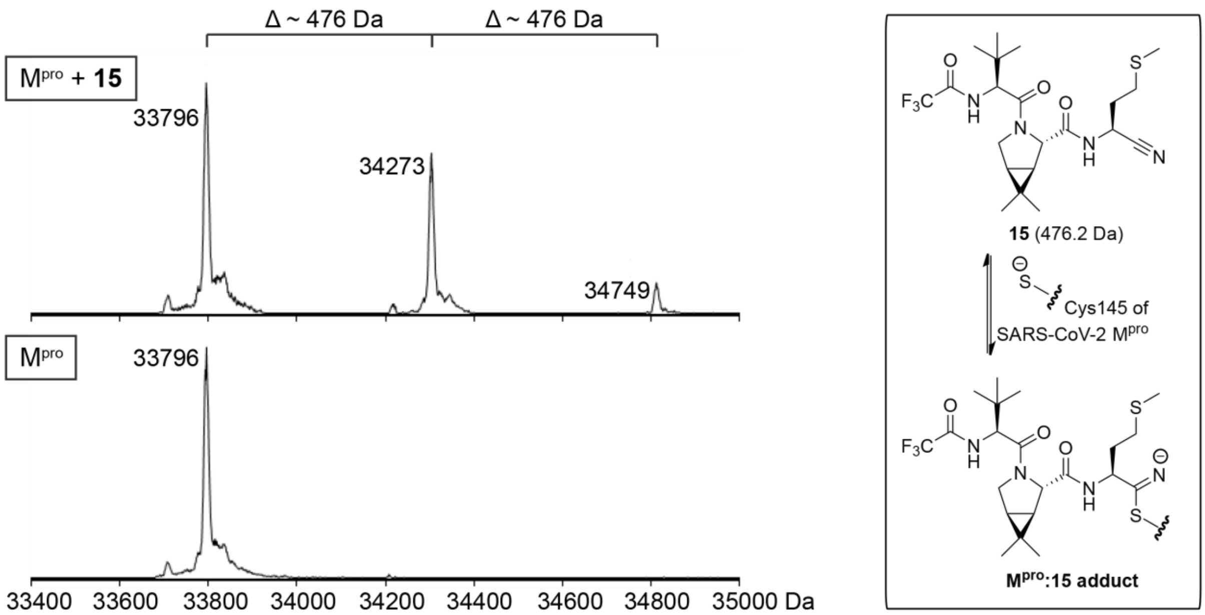

Supporting Figure S14. Mass spectrometric evidence that the MI-09 alkyne derivative 18 reacts covalently with the nucleophilic Cys145 of SARS-CoV-2 M^{pro}. Protein-observed MS studies were performed as reported using SPE-MS.[1-2] Conditions: SARS-CoV-2 M^{pro} (2.0 µM) was incubated with MI-09[13] derivative **18** (10 µM) in buffer (20 mM HEPES, pH 7.5; 50 µL total reaction volume) for 90 min at ambient temperature, before stopping the reaction by addition of 1%$_{v/v}$ aqueous formic acid (5 µL) and analysis by SPE-MS using reported parameters.[1-2]

The MI-09[13] alkyne derivative **18** reacts covalently with SARS-CoV-2 M^{pro} as revealed by the anticipated ~508 Da mass shift with respect to unmodified M^{pro} (33796 Da), likely with the nucleophilic Cys145 as crystallographically observed by reaction of alkyne **13** with M^{pro} (Figure 4). Alkyne **18** appears to react less efficiently with M^{pro} than alkyne **13**, in accord with its ~20-fold higher IC_{50} value (Table 2). Note that the minor peak corresponding to a second M^{pro} modification (*i.e.* 34810 Da) may be an artefact due to non-active site binding.

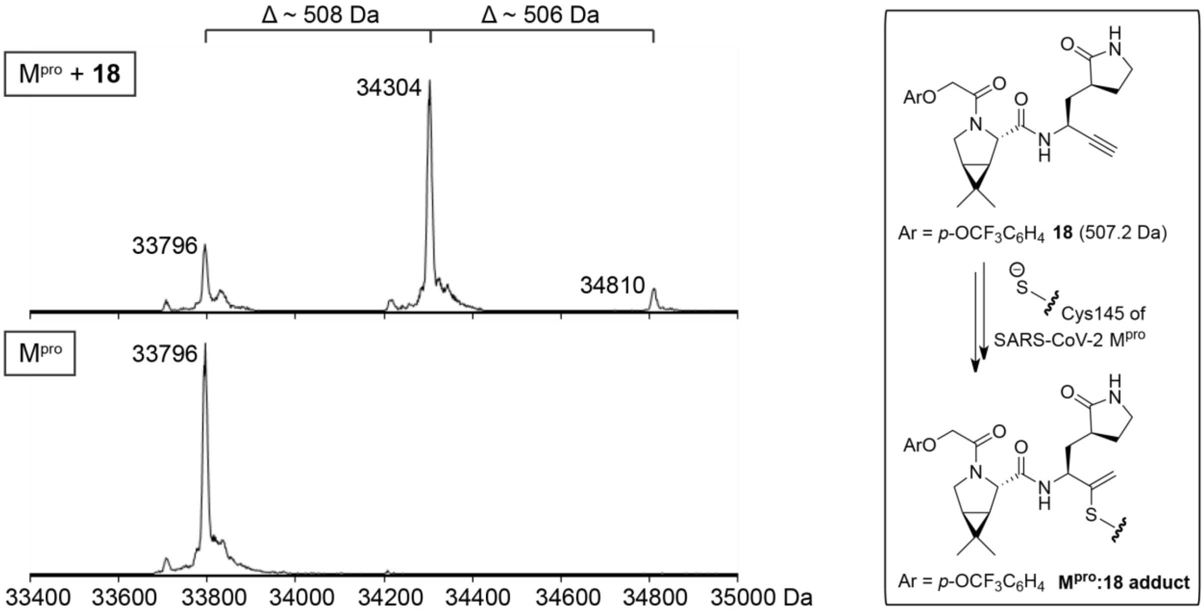

Supporting Figure S15. Mass spectrometric evidence that the MI-09 alkyne derivative 19 reacts covalently with the nucleophilic Cys145 of SARS-CoV-2 M^{pro}. Protein-observed MS studies were performed as reported using SPE-MS.[1-2] Conditions: SARS-CoV-2 M^{pro} (2.0 μM) was incubated with MI-09[13] derivative **19** (10 μM) in buffer (20 mM HEPES, pH 7.5; 50 μL total reaction volume) for 90 min at ambient temperature, before stopping the reaction by addition of $1\%_{v/v}$ aqueous formic acid (5 μL) and analysis by SPE-MS using reported parameters.[1-2]

The MI-09[13] alkyne derivative **19** reacts covalently with SARS-CoV-2 M^{pro} as revealed by the anticipated ~491 Da mass shift with respect to unmodified M^{pro} (33796 Da), likely with the nucleophilic Cys145 as crystallographically observed by reaction of alkyne **13** with M^{pro} (Figure 4). Alkyne **19** appears to react less efficiently with M^{pro} than alkyne **13**, in accord with its ~20-fold higher IC_{50} value (Table 2). Note that the minor peak corresponding to a second M^{pro} modification (*i.e.* 34779 Da) may be an artefact due to non-active site binding.

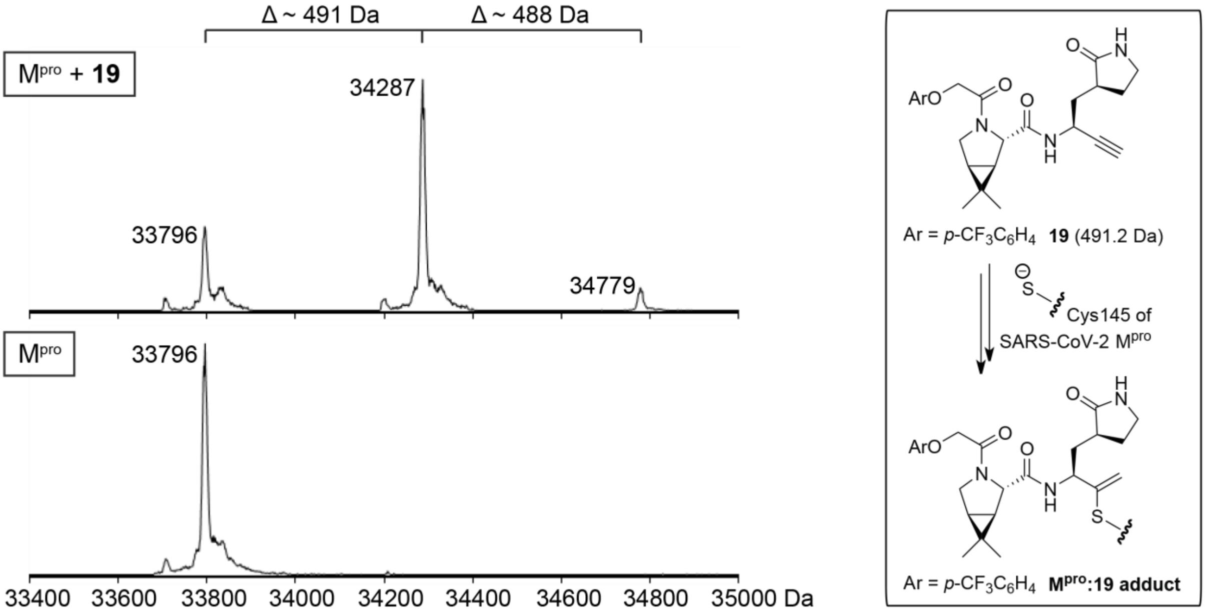

Supporting Figure S16. Mass spectrometric evidence that the MI-09 nitrile derivative 20 reacts covalently with the nucleophilic Cys145 of SARS-CoV-2 M^{pro}. Protein-observed MS studies were performed as reported using SPE-MS.[1-2] Conditions: SARS-CoV-2 M^{pro} (2.0 µM) was incubated with MI-09[13] derivative **20** (10 µM) in buffer (20 mM HEPES, pH 7.5; 50 µL total reaction volume) for 90 min at ambient temperature, before stopping the reaction by addition of $1\%_{v/v}$ aqueous formic acid (5 µL) and analysis by SPE-MS using reported parameters.[1-2]

The MI-09[13] nitrile derivative **20** reacts covalently with SARS-CoV-2 M^{pro} as revealed by the anticipated ~509 Da mass shift with respect to unmodified M^{pro} (33796 Da), likely with the nucleophilic Cys145 as crystallographically observed by reaction of **1** with M^{pro} (Figure 1). Nitrile **15** appears to react less efficiently with M^{pro} than nitrile **1**, in accord with its ~5-fold higher IC_{50} value (Tables 1 and 2). Note that the minor peak corresponding to a second M^{pro} modification (*i.e.* 34812 Da) may be an artefact due to non-active site binding.

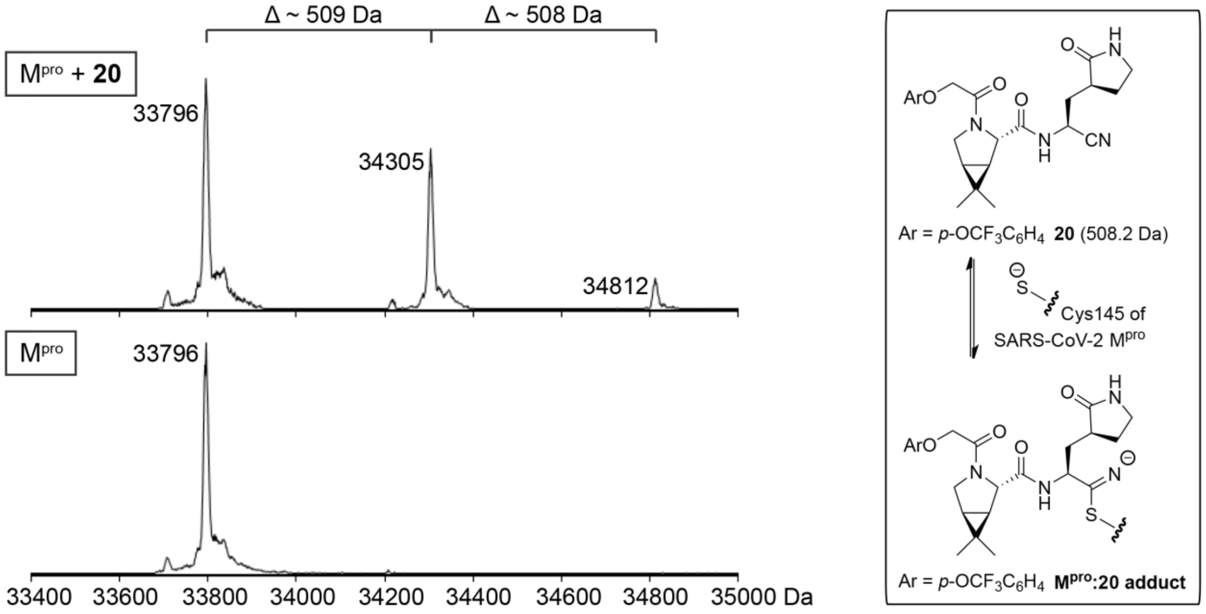

Supporting Figure S17. Mass spectrometric evidence that the MI-09 nitrile derivative 21 reacts covalently with the nucleophilic Cys145 of SARS-CoV-2 M^{pro}. Protein-observed MS studies were performed as reported using SPE-MS.[1-2] Conditions: SARS-CoV-2 M^{pro} (2.0 μM) was incubated with MI-09[13] derivative **21** (10 μM) in buffer (20 mM HEPES, pH 7.5; 50 μL total reaction volume) for 90 min at ambient temperature, before stopping the reaction by addition of 1%$_{v/v}$ aqueous formic acid (5 μL) and analysis by SPE-MS using reported parameters.[1-2]

The MI-09[13] nitrile derivative **21** reacts covalently with SARS-CoV-2 M^{pro} as revealed by the anticipated ~491 Da mass shift with respect to unmodified M^{pro} (33796 Da), likely with the nucleophilic Cys145 as crystallographically observed by reaction of **1** with M^{pro} (Figure 1). Nitrile **15** appears to react less efficiently with M^{pro} than nitrile **1**, in accord with its ~5-fold higher IC_{50} value (Tables 1 and 2), but with similar efficiency as nitrile **20**. Note that the minor peak corresponding to a second M^{pro} modification (*i.e.* 34779 Da) may be an artefact due to non-active site binding.

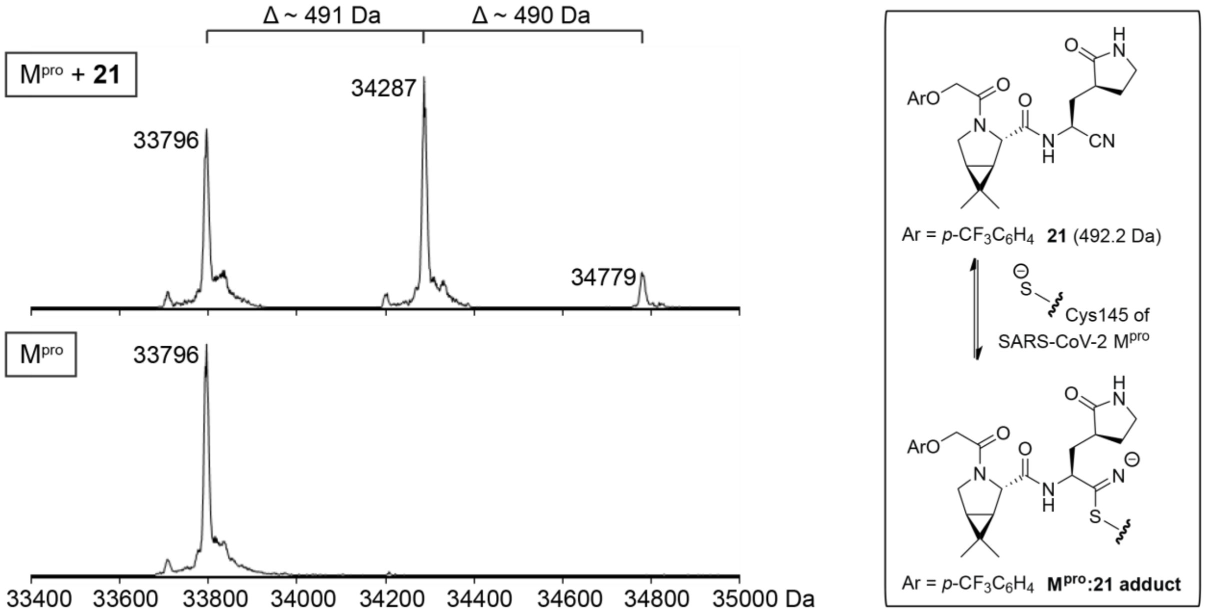

Supporting Figure S18. Mass spectrometric evidence that the GC376 alkyne derivative 23 reacts covalently with the nucleophilic Cys145 of SARS-CoV-2 M^{pro}. Protein-observed MS studies were performed as reported using SPE-MS.[1-2] Conditions: SARS-CoV-2 M^{pro} (2.0 µM) was incubated with GC376[16] derivative **23** (10 µM) in buffer (20 mM HEPES, pH 7.5; 50 µL total reaction volume) for 90 min at ambient temperature, before stopping the reaction by addition of 1%$_{v/v}$ aqueous formic acid (5 µL) and analysis by SPE-MS using reported parameters.[1-2]

The GC376[16] alkyne derivative **23** reacts covalently with SARS-CoV-2 M^{pro} as revealed by the anticipated ~399 Da mass shift with respect to unmodified M^{pro} (33796 Da), likely with the nucleophilic Cys145 as crystallographically observed by reaction of alkyne **13** with M^{pro} (Figure 4). Alkyne **23** appears to react less efficiently with M^{pro} than alkyne **13**, in accord with its ~30-fold higher IC_{50} value (Tables 1 and 2).

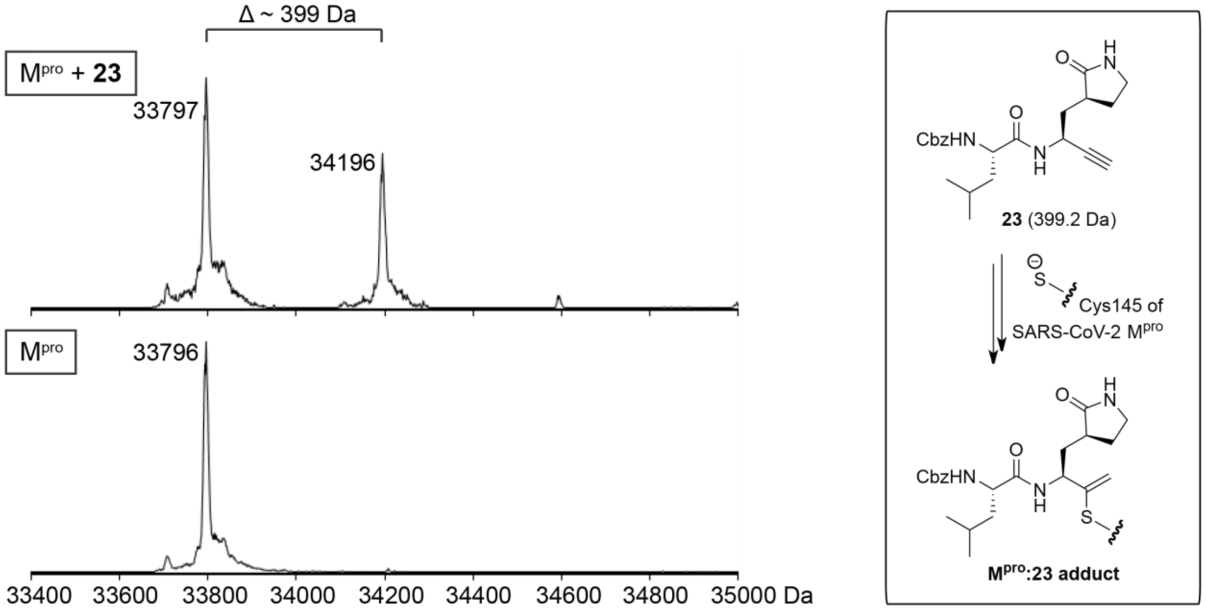

Supporting Figure S19. Mass spectrometric evidence that the alkyne 27a reacts covalently with the nucleophilic Cys145 of SARS-CoV-2 M^{pro}. Protein-observed MS studies were performed as reported using SPE-MS.[1-2] Conditions: SARS-CoV-2 M^{pro} (2.0 µM) was incubated with nirmatrelvir derivative **27a** (10 µM) in buffer (20 mM HEPES, pH 7.5; 50 µL total reaction volume) for 90 min at ambient temperature, before stopping the reaction by addition of $1\%_{v/v}$ aqueous formic acid (5 µL) and analysis by SPE-MS using reported parameters.[1-2] The alkyne derivative **27a** reacts covalently with SARS-CoV-2 M^{pro} as revealed by the anticipated ~572 Da mass shift with respect to unmodified M^{pro} (33796 Da), likely with the nucleophilic Cys145 as crystallographically observed by reaction of alkyne **13** with M^{pro} (Figure 4). Alkyne **27a** appears to react substantially less efficiently with M^{pro} than alkyne **13**, in accord with its ~80-fold higher IC_{50} value (Tables 1 and 3).

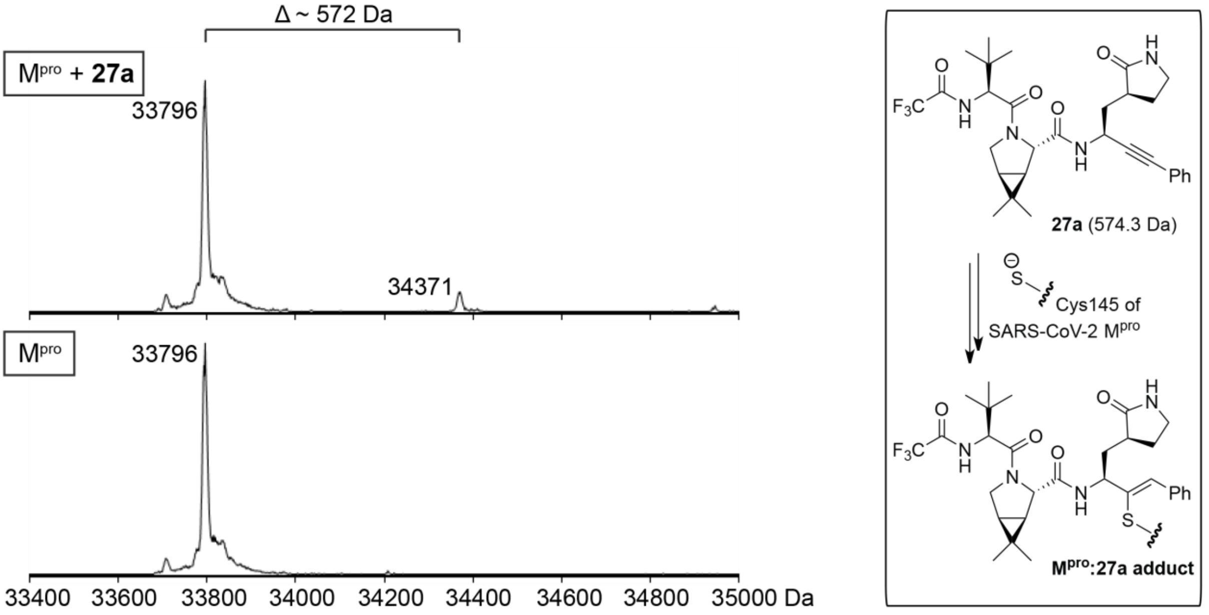

Supporting Figure S20. Mass spectrometric evidence that the alkyne 27b reacts covalently with the nucleophilic Cys145 of SARS-CoV-2 M^{pro}. Protein-observed MS studies were performed as reported using SPE-MS.[1-2] Conditions: SARS-CoV-2 M^{pro} (2.0 µM) was incubated with nirmatrelvir derivative **27b** (10 µM) in buffer (20 mM HEPES, pH 7.5; 50 µL total reaction volume) for 90 min at ambient temperature, before stopping the reaction by addition of 1%$_{v/v}$ aqueous formic acid (5 µL) and analysis by SPE-MS using reported parameters.[1-2] The alkyne derivative **27b** reacts covalently with SARS-CoV-2 M^{pro} as revealed by the anticipated ~619 Da mass shift with respect to unmodified M^{pro} (33796 Da), likely with the nucleophilic Cys145 as crystallographically observed by reaction of alkyne **13** with M^{pro} (Figure 4). Alkyne **27b** appears to react substantially less efficiently with M^{pro} than alkyne **13**, in accord with its ~50-fold higher IC$_{50}$ value, but apparently slightly more efficient than alkyne **27a** (Tables 1 and 3).

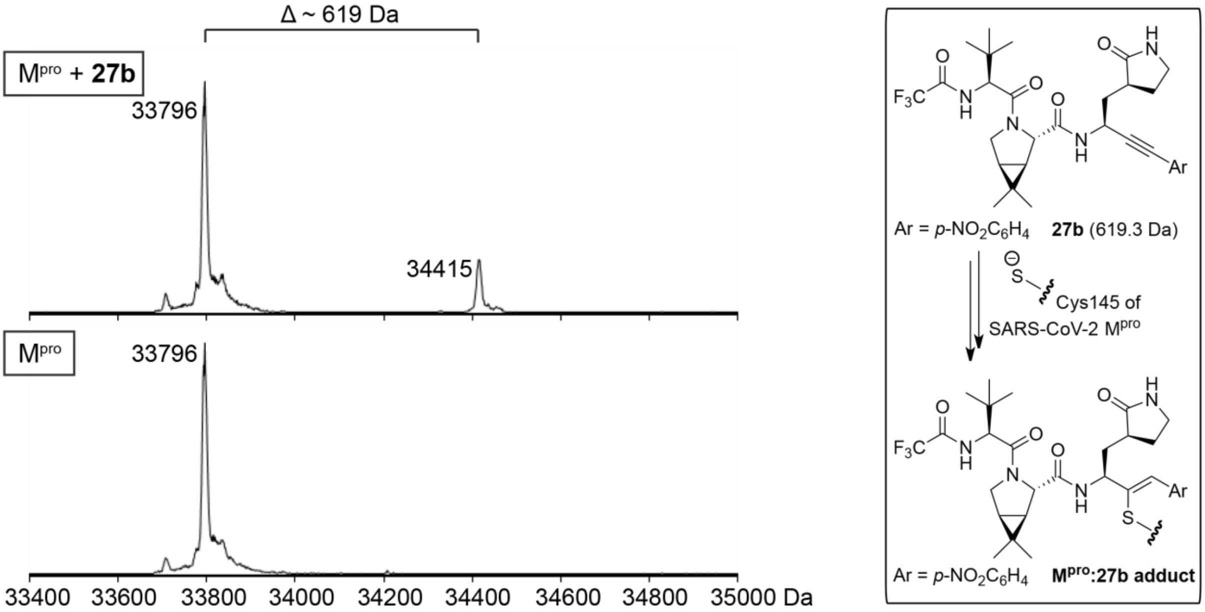

Supporting Figure S21. Mass spectrometric evidence that the alkyne 28 reacts covalently with the nucleophilic Cys145 of SARS-CoV-2 M^{pro}. Protein-observed MS studies were performed as reported using SPE-MS.[1-2] Conditions: SARS-CoV-2 M^{pro} (2.0 µM) was incubated with nirmatrelvir derivative **28** (10 µM) in buffer (20 mM HEPES, pH 7.5; 50 µL total reaction volume) for 90 min at ambient temperature, before stopping the reaction by addition of 1%$_{v/v}$ aqueous formic acid (5 µL) and analysis by SPE-MS using reported parameters.[1-2] The alkyne derivative **28** reacts covalently with SARS-CoV-2 M^{pro} as revealed by the anticipated ~572 Da mass shift with respect to unmodified M^{pro} (33796 Da), likely with the nucleophilic Cys145 as crystallographically observed by reaction of alkyne **13** with M^{pro} (Figure 4). Alkyne **28** appears to react substantially less efficiently with M^{pro} than alkyne **13**, despite their similar IC$_{50}$ values (Tables 1 and 3). Note, however, that time course experiments have not yet been performed to validate this observation.

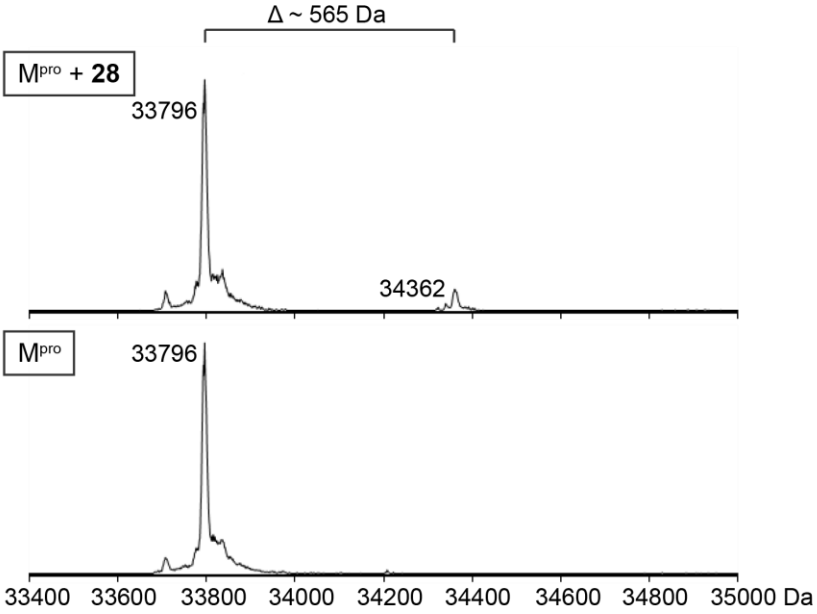

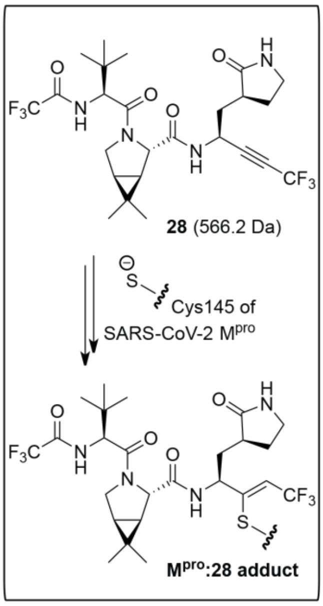

2. Supporting Tables

Supporting Table S1. Cell viability assays indicate that nirmatrelvir and selected derivatives are not cytotoxic over the tested concentration range.

	**Nirmatrelvir derivative**	[a]**Cytotoxicity** [μM]
i	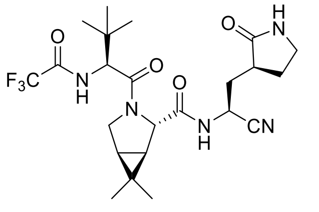 **nirmatrelvir (1)**	>100
ii	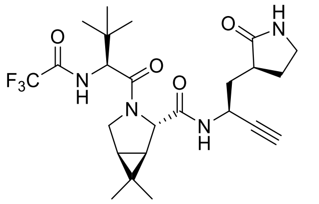 **13**	>100
iii	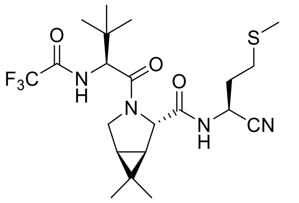 **15**	>100

a) Inhibitor toxicity was assayed using the MTT (3-(4,5-dimethylthiazol-2-yl)-2,5-diphenyltetrazolium bromide) Cell Proliferation assay kit (Abcam – Ab211091) as per the manufacturers' recommendation (for details, see Experimental Section). Results are a mean of independent triplicates (n = 3; mean ± standard deviation, SD).

Supporting Table S2. Alkyne- and nitrile bearing SARS-CoV-2 M^pro^ inhibitors retain activity against the S144A SARS-CoV-2 M^pro^ variant (continues on the following page). SPE-MS SARS-CoV-2 M^pro^ inhibition assays were performed as described (Experimental Section). Note that the S144A M^pro^ inhibition assays differed from those of wildtype (WT) M^pro^ by use of twice the M^pro^ concentration (0.1 µM instead of 0.05 µM used for WT M^pro^).

Consistent with the literature,[17-18] the SPE-MS results reveal that nirmatrelvir (**1**) inhibits S144A M^pro^ less efficiently than WT M^pro^ (~4-fold; Entry i). Similarly, the inhibition efficiency of the alkyne derivatives **13**, **14**, and **28** for inhibiting S144A M^pro^ decreased ~4- to ~6-fold compared to WT M^pro^ (Entries, ii, iii, and xii), whereas the inhibition efficiency of the phenyl-capped derivatives **27a** and **27b** appeared to decrease less (Entries x and xi). Note, however, that the standard deviation of the inhibition results obtained for S144A M^pro^ appeared to be higher than those obtained for WT M^pro^, which, at least in part, reflects the reduced robustness of the S144A M^pro^ inhibition assay as indicated by typically lower Z'-factors[12] than observed for the WT M^pro^ inhibition assays (Supporting Figure S5); however, Z'-factors[12] of the S144A M^pro^ inhibition assays were >0.5.

Interestingly, MI-09[13] derived nitrile **20** appeared to inhibit S144A M^pro^ ~20-fold less efficiently than WT M^pro^, whereas the corresponding alkyne **18** appeared to inhibit S144A M^pro^ only ~3-fold less efficiently than WT M^pro^ (Entries iv and v). This observation indicates that the effect of M^pro^ active site variations on the inhibition efficiency of alkyne- and nitrile-based inhibitors may differ. This observation also highlights the possibility that alkyne-based inhibitors of WT M^pro^ and M^pro^ active site variants can be developed that are more efficient than nirmatrelvir or other covalent inhibitors with functional groups other than nitriles, provided that the P2-P4 equivalent positions are carefully optimized. By contrast, GC376 (**3**)[16] and its alkyne derivative **23** both inhibited S144A M^pro^ ~3-fold less efficiently than WT M^pro^ (Entries viii and ix).

	SARS-CoV-2 M^pro^ inhibitor	[a)] IC50 WT M^pro^ [µM]	[b)] IC50 S144A M^pro^ [µM]		SARS-CoV-2 M^pro^ inhibitor	[a)] IC50 WT M^pro^ [µM]	[b)] IC50 S144A M^pro^ [µM]
i	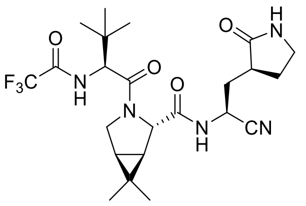 **nirmatrelvir (1)**	≤0.025	0.09 ± 0.01	iv	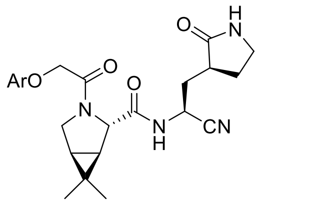 Ar = *p*-$OCF_3C_6H_4$ **20**	0.16 ± 0.03	3.2 ± 0.8
ii	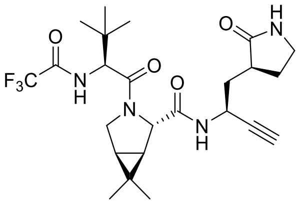 **13**	0.14 ± 0.03	0.58 ± 0.14	v	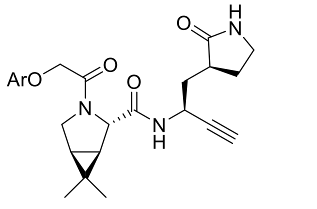 Ar = *p*-$OCF_3C_6H_4$ **18**	2.8 ± 0.8	7.5 ± 3.1
iii	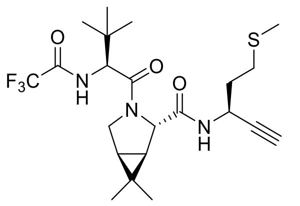 **14**	11.9 ± 1.2	49.8 ± 17.4	vi	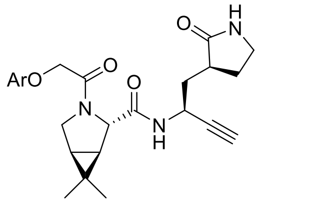 Ar = *p*-$CF_3C_6H_4$ **19**	3.0 ± 0.4	14.5 ± 11.9

	SARS-CoV-2 M^{pro} inhibitor	[a]IC_{50} WT M^{pro} [μM]	[b]IC_{50} S144A M^{pro} [μM]		SARS-CoV-2 M^{pro} inhibitor	[a]IC_{50} WT M^{pro} [μM]	[b]IC_{50} S144A M^{pro} [μM]
vii	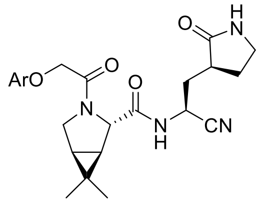 Ar = *p*-$CF_3C_6H_4$ **21**	0.16 ± 0.05	4.4 ± 1.4	x	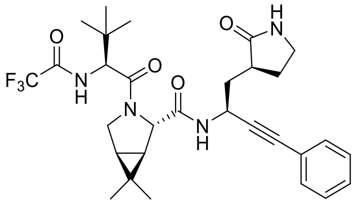 **27a**	11.0 ± 3.5	15.8 ± 10.3
viii	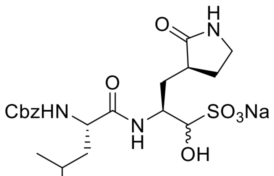 **GC376 (3)**	≤0.025	0.08 ± 0.01	xi	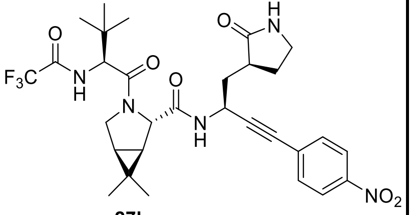 **27b**	7.1 ± 2.6	20.1 ± 12.2
ix	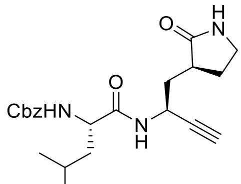 **23**	4.7 ± 1.8	14.0 ± 8.1	xii	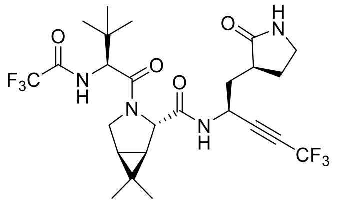 **28**	0.23 ± 0.02	1.3 ± 0.2

a) Inhibition assays were performed using SPE-MS as described employing SARS-CoV-2 M^{pro} (0.05 μM) and ALNDFSNSGSDVLYQPPQTSITSAVLQ/SGFRKMAFPS-NH_2 as a substrate (2.0 μM) in the presence of an internal standard (Supporting Figures S1 and S2);[1-2] b) S144A SARS-CoV-2 M^{pro} inhibition assays were performed using SPE-MS employing SARS-CoV-2 S144A M^{pro} (0.1 μM) and ALNDFSNSGSDVLYQPPQTSITSAVLQ/SGFRKMAFPS-NH_2 as a substrate (2.0 μM) in the presence of an internal standard as described for WT M^{pro}; note that the concentration of S144A M^{pro} was double that of WT M^{pro} to account for its reduced catalytic activity. Data are presented as a mean of independent triplicates, each composed of technical duplicates (n = 3; mean ± SD).

Supporting Table S3. Alkyne- and nitrile bearing SARS-CoV-2 M^pro^ inhibitors do not inhibit SARS-CoV-2 PL^pro^ (continues on the following page). SPE-MS SARS-CoV-2 PL^pro^ inhibition assays were performed as reported using isolated recombinant PL^pro^.[3] Ebselen (Entry i), which is a reported efficient M^pro^ and PL^pro^ inhibitor, was used as a positive inhibition control and inhibits PL^pro^ in the expected range (IC_{50} ~ 0.1 μM, Entry i; reported IC_{50} ~ 0.3 μM[3]).

Nirmatrelvir (**1**) and the other trialed nitrile-bearing SARS-CoV-2 M^pro^ inhibitors **15**, **20**, and **21** did not inhibit PL^pro^ over the tested concentration range (Entries ii-v), in accord with the reported lack of PL^pro^ inhibition by **1**.[3] Although covalent alkyne SARS-CoV-2 PL^pro^ inhibitors have been reported,[19-20] alkyne derivatives **13**, **14**, **27a**, **27b**, and **28** did not inhibit PL^pro^ over the tested concentration range (Entries vi-x). In addition, neither the MI-09[13] derived alkynes **18** and **19**, nor GC376 (**3**)[16] and its alkyne derivative **23** inhibited isolated recombinant SARS-CoV-2 PL^pro^ at substantial levels (Entries xi-xiv).

	**SARS-CoV-2 M^pro^ inhibitor**	^a)^**IC_{50}** [μM]		**SARS-CoV-2 M^pro^ inhibitor**	^a)^**IC_{50}** [μM]
i	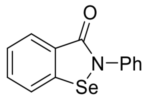 **ebselen**	0.10 ± 0.01	vi	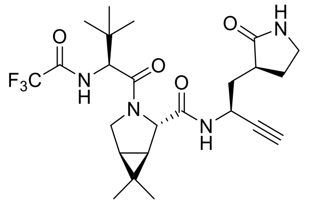 **13**	>100
ii	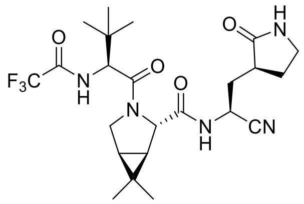 **nirmatrelvir (1)**	>100	vii	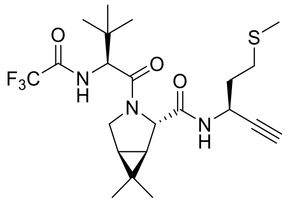 **14**	>100
iii	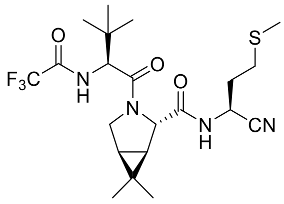 **15**	>100	viii	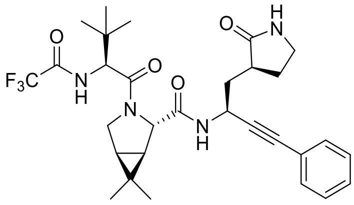 **27a**	>100
iv	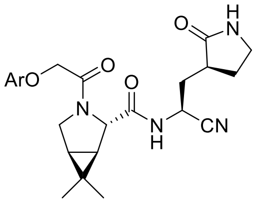 Ar = *p*-$OCF_3C_6H_4$ **20**	>100	ix	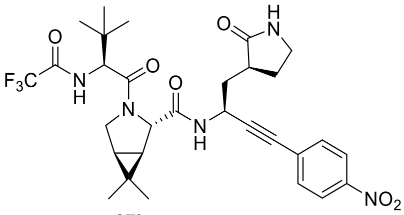 **27b**	>100
v	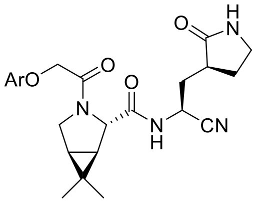 Ar = *p*-$CF_3C_6H_4$ **21**	>100	x	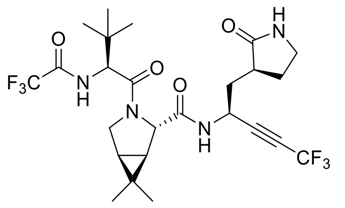 **28**	>100

	SARS-CoV-2 M^pro inhibitor	a)IC50 [μM]		SARS-CoV-2 M^pro inhibitor	a)IC50 [μM]
xi	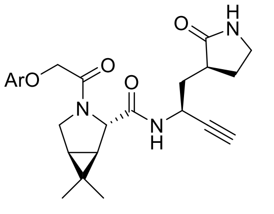 Ar = *p*-$OCF_3C_6H_4$ **18**	>100	xiii	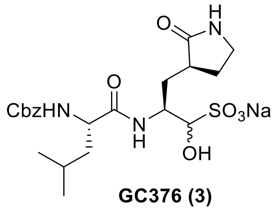 **GC376 (3)**	>100
xii	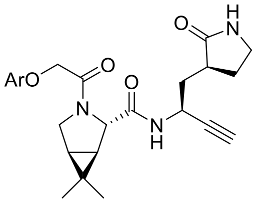 Ar = *p*-$CF_3C_6H_4$ **19**	>100	xiv	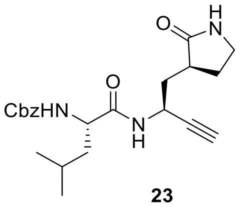 **23**	>100

a) SARS-CoV-2 PL^{pro} inhibition assays were performed using SPE-MS as reported[3]. Data are presented as a mean of independent duplicates, each composed of technical duplicates (n = 2; mean ± SD).

Supporting Table S4. Data collection and refinement statistics for the SARS-CoV-2 M^{pro}:13 complex.

	SARS-CoV-2 M^{pro}:**13** complex
**PDB ID**	8B2T
**Data collection**	
Space group	$P2_12_12$
Symmetry	orthorhombic
Cell dimensions	
a, *b*, *c* (Å)	46.20, 64.65, 101.81
α, β, γ (°)	90.0, 90.0, 90.0
Resolution (Å)	11.53-1.89 (1.94-1.89)
R_{merge}	0.543 (7.22)
$I / \sigma I$	7.6 (1.5)
CC (1/2)	0.98 (0.48)
Completeness (%)	79.1 (51.8)
Redundancy	16.4 (22.4)
**Refinement**	
Resolution (Å)	22.54-1.90
No. reflections	23137
R_{work} / R_{free}	0.182 / 0.2223
No. atoms	
Protein	2370
Ligand/ion	35
Water	83
B-factors	
Protein	32.9
Ligand/ion	26.9
Water	33.3
R.m.s. deviations	
Bond lengths (Å)	0.006
Bond angles (°)	1.42

*Values in parentheses are for highest-resolution shell.

3. General synthesis information

All reagents were from commercial sources (Sigma-Aldrich, Inc.; Fluorochem Ltd; Ambeed, Inc.) and used as received. Anhydrous solvents (Sigma-Aldrich, Inc.) were kept under an atmosphere of nitrogen. Nirmatrelvir (**1**) was synthesized according to Pfizer's published synthesis[8] and, in addition, commercially-sourced (AstaTech, Inc.). (1*R*,2*S*,5*S*)-3-((*S*)-3,3-Dimethyl-2-(2,2,2-trifluoroacetamido)butanoyl)-6,6-dimethyl-3-azabicyclo[3.1.0]hexane-2-carboxylic acid (**12**) was synthesized from commercially-sourced materials as reported,[8] with the exception that COMU[9] was used instead of HATU as amide coupling reagent. Note that final compounds were purified by reverse phase high performance liquid chromatography (HPLC) rather than crystallization/tituration due to the smaller synthesis scale compared to Pfizer's synthesis.[8]

Purifications were performed using a Biotage Isolera One or a Biotage Selekt purification machine (wavelength monitored: 254 and 280 nm) equipped with pre-packed Biotage® SFär flash chromatography cartridges. The cartridge type and size as well as solvent gradients (in column volumes, CV) used, are specified in the individual experimental procedures. HPLC grade solvents (Sigma-Aldrich Inc.) were used for purifications, reaction work-ups, and extractions. Reversed-phase HPLC purifications were performed using a semi-preparative HPLC machine (Shimadzu UK Ltd.) equipped with a reverse phase column (ACE 10 AQ, dimensions: 250 mm length, 21.2 mm inner diameter, 10.0 μm particle size). A linear gradient of acetonitrile in water (each containing 0.1%$_{v/v}$ formic acid) was used as eluent (flow rate: 20 mL/min; wavelength monitored: 220 nm). Fractions were lyophilized and analyzed using ^{1}H NMR.

Thin layer chromatography (TLC) was carried out using Merck silica gel 60 F_{254} TLC plates and visualized using UV light or ninhydrin stain. Infrared (IR) spectroscopy was performed using a Bruker Tensor-27 Fourier transform infrared (FT-IR) spectrometer. High-resolution mass spectrometry (HRMS) was performed using electrospray ionization (ESI) mass spectrometry (MS) in the positive or negative ionization mode employing a Thermo Scientific Exactive mass spectrometer (ThermoFisher Scientific); data are presented as a mass-to-charge ratio (*m/z*).

Nuclear magnetic resonance (NMR) spectroscopy was performed using a Bruker AVANCE AVIIIHD 600 machine equipped with a 5 mm BB-F/1H Prodigy N_2 cryoprobe or a Bruker AVIIIHD 400 nanobay machine. Chemical shifts for protons are reported in parts per million (ppm) downfield from tetramethylsilane and are referenced to residual protium in the NMR solvent (*i.e.* DMSO-d_6: δ = 2.49 ppm; CD_3OD: δ = 3.31 ppm; D_2O: δ = 4.79 ppm; $CDCl_3$: δ = 7.28 ppm). For ^{13}C NMR, chemical shifts are reported in the scale relative to the NMR solvent (*i.e.* DMSO-d_6: δ = 39.52 ppm; $CDCl_3$: δ = 77.0 ppm). For ^{19}F NMR, chemical shifts are reported in the scale relative to $CFCl_3$. NMR data are reported as follows: chemical shift, multiplicity (s: singlet, d: doublet, dd: doublet of doublets, t: triplet, q: quartet, m: multiplet, br: broad signal), coupling constant (*J*, Hz; accurate to 0.1 Hz), and integration. The number of C-atoms in brackets indicates overlapping signals in ^{13}C NMR; chemical shift numbers in brackets indicate close signals that can be differentiated by considering the second decimal numbers. All compounds are >95% pure by NMR and HPLC analysis unless stated otherwise, NMR spectra are shown in Section 7 of the Supporting Information.

In agreement with the reported NMR spectra of nirmatrelvir (**1**) and derivatives,[8] minor amounts of additional species were present in NMR spectra, which are likely rotamers as their ratio changes depending on the solvent used.

4. General synthetic procedures

General Procedure A. To a solution of a methyl ester (1.0 equiv.) in anhydrous tetrahydrofuran (THF; 0.3 M) was added an aqueous LiOH solution (2 M; 3.0 equiv.) under an ambient atmosphere at 0 °C. The reaction mixture was stirred overnight whilst slowly warming to ambient temperature, before THF was evaporated. The pH of the aqueous phase was adjusted to pH 2 using aqueous HCl (1 M), followed by extraction with ethyl acetate (three times). The combined organic extracts were washed with brine, dried over anhydrous Na_2SO_4, filtered, and evaporated to afford the corresponding carboxylic acid, which was used in the next step without further purification.

General Procedure B. To a solution of a Boc-protected amino acid (1.0 equiv.) in anhydrous CH_2Cl_2 (0.25 M) was added 1,1'-carbonyldiimidazole (1.2 equiv.) at ambient temperature under a nitrogen atmosphere. The reaction mixture was stirred for until CO_2 evolution ceased (at least 30 min), before being cooled to 0 °C followed by the subsequent addition of *N*,*N*-diisopropylethylamine (1.5 equiv.) and *N*,*O*-dimethyl hydroxylamine hydrochloride (1.5 equiv.). The reaction mixture was stirred overnight (~14 h) under nitrogen atmosphere whilst slowly warming up to room temperature, before being diluted with deionized water at 0 °C. The mixture was extracted with CH_2Cl_2 (three times); the combined organic extracts were washed with brine, dried over anhydrous Na_2SO_4, filtered, evaporated, and purified by column chromatography to afford the corresponding Weinreb amide.

General Procedure C. To a solution of the Ohira-Bestmann reagent **10**[6-7] (1.2 equiv.) in anhydrous methanol (0.6 M) was added K_2CO_3 (2.0 equiv.) and the resultant suspension was stirred for 10 min under nitrogen atmosphere at ambient temperature. Then, a solution of an aldehyde (1.0 equiv.) in anhydrous methanol (1 M) was added to the suspension and the reaction mixture was stirred at room temperature overnight (~14 h), before being diluted with deionized water. The mixture was extracted with diethyl ether (three times); the combined organic extracts were washed with brine, dried over anhydrous Na_2SO_4, filtered, evaporated, and purified by column chromatography to afford the corresponding terminal alkyne.

General Procedure D. To a solution of a *N*-Boc-protected amine (1.0 equiv.) in anhydrous CH_2Cl_2 (1 M) was added a HCl solution (4 N in 1,4 dioxane; 5.0 equiv.). The reaction mixture was stirred overnight (~14 h) at ambient temperature under an ambient atmosphere, before being evaporated to afford the corresponding amine hydrochloride salt, which was used in the next step without further purification.

General Procedure E. To a solution of an amine hydrochloride salt (1.0 equiv.) and a carboxylic acid (1.2 equiv.) in a 1:1 (v/v) mixture of anhydrous DMF (0.46 M) and anhydrous CH_2Cl_2 (0.46 M) were sequentially added *N*-methyl morpholine (NMM; 3.0 equiv.) and (1-cyano-2-ethoxy-2-oxoethylidenaminooxy)dimethylamino-morpholino-carbenium hexafluorophosphate (COMU)[9] (1.2 equiv.) at 0 °C. The reaction mixture was stirred overnight (~14 h) under nitrogen atmosphere whilst slowly warming up to room temperature, before being diluted with aqueous HCl solution (1 M). The mixture was extracted with ethyl acetate (three times); the combined organic extracts were sequentially washed with a saturated aqueous $NaHCO_3$ solution and brine, dried over anhydrous Na_2SO_4, filtered, evaporated, and purified using HPLC. Fractions containing the corresponding pure amide coupling product were lyophilized.

General Procedure F. To a solution of an amide (1.0 equiv.) in anhydrous CH_2Cl_2 (0.22 M) was added the Burgess reagent (**42**)[10-11] (2.48 equiv.). The reaction mixture was stirred at ambient temperature under nitrogen atmosphere for 1 h, before being diluted with a 2:1 (v/v) mixture of a saturated aqueous $NaHCO_3$ solution and brine. The organic layer was separated, dried over anhydrous Na_2SO_4, filtered, evaporated, and purified using HPLC.

Fractions containing the corresponding pure nitrile coupling product were lyophilized.

General Procedure G. A solution of alkyne **24** (1.0 equiv.) and an aryl iodide (1.3 equiv.) in a 1:1 (v/v) mixture of anhydrous THF and triethylamine (0.2 M) was degassed with nitrogen for 5 min in a reaction tube. Bis(triphenylphosphine)palladium(II) dichloride (2.5 mol%) and CuI (5 mol%) were added to the mixture and the resultant suspension was degassed for another 5 min with nitrogen. The reaction tube was then sealed and the mixture was stirred at 80 °C for 3 h, before being cooled to room temperature. The mixture was diluted with ethyl acetate and sequentially washed with a saturated aqueous NH_4Cl solution, water, and brine; the organic layer was dried over anhydrous Na_2SO_4, filtered, evaporated, and purified by column chromatography to afford the corresponding aryl substituted alkyne.

5. Experimental procedures and compound characterizations

(*S*)-2-((*tert*-Butoxycarbonyl)amino)-3-((*S*)-2-oxopyrrolidin-3-yl)propanoic acid (46). According to General Procedure A, carboxylic acid **46** (2.78 g, 83%) was obtained from commercially-sourced methyl (*S*)-2-((*tert*-butoxycarbonyl)amino)-3-((*S*)-2-oxopyrrolidin-3-yl)propanoate (**8**; 3.5 g, 12.2 mmol). **46** was used in the next step without further purification. White amorphous solid; ^{1}H NMR (600 MHz, 300 K, DMSO-d_6): δ = 12.50 (s, 1H), 7.61 (s, 1H), 7.22 (d, J = 8.2 Hz, 1H), 3.93−3.89 (m, 1H), 3.16−3.10 (m, 2H), 2.27−2.21 (m, 1H), 2.16−2.11 (m, 1H), 1.98−1.93 (m, 1H), 1.65−1.58 (m, 1H), 1.57−1.50 (m, 1H), 1.37 ppm (s, 9H); ^{13}C NMR (150 MHz, 300 K, DMSO-d_6): δ = 178.2, 174.0, 155.6, 78.0, 51.8, 37.8, 32.4, 30.7, 28.2, 27.3 ppm; IR (film): ṽ = 3319, 2977, 1688, 1524, 1444, 1393, 1367, 1249, 1164, 1051 cm^{-1}; HRMS (ESI): *m/z* calculated for $C_{12}H_{21}O_5N_2$ [M+H]$^+$: 273.1445, found: 273.1442.

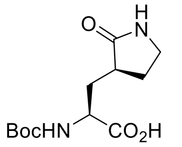

***tert*-Butyl ((*S*)-1-(methoxy(methyl)amino)-1-oxo-3-((*S*)-2-oxopyrrolidin-3-yl)propan-2-yl)carbamate (47).** According to General Procedure B, amide **47** (1857 mg, 58%) was obtained from carboxylic acid **46** (2750 mg, 10.1 mmol), following column chromatography (50 g Sfär cartridge; 120 mL/min; initially, 100%$_{v/v}$ cyclohexane (2 CV), followed by a linear gradient (15 CV): 0%$_{v/v}$ → 100%$_{v/v}$ acetone in cyclohexane). White amorphous solid; ^{1}H NMR (600 MHz, 300 K, DMSO-d_6): δ = 7.60 (s, 1H), 7.16 (brd, J = 8.3 Hz, 1H), 4.42−4.40 (m, 1H), 3.71 (s, 3H), 3.14−3.12 (m, 2H), 3.09 (s, 3H), 2.27−2.25 (m, 1H), 2.17−2.13 (m, 1H), 1.91−1.87 (m, 1H), 1.64−1.59 (m, 1H), 1.40−1.38 (m, 1H), 1.36 ppm (s, 9H); ^{13}C NMR (150 MHz, 300 K, DMSO-d_6): δ = 178.1, 172.7 (br), 155.6, 78.0, 61.1, 49.1, 37.7, 32.3, 32.0, 28.2, 28.1, 27.2 ppm; IR (film): ṽ = 3304, 2980, 1689, 1524, 1442, 1392, 1367, 1253, 1168, 1049, 1018 cm^{-1}; HRMS (ESI): *m/z* calculated for $C_{14}H_{26}O_5N_3$ [M+H]$^+$: 316.1867, found: 316.1861.

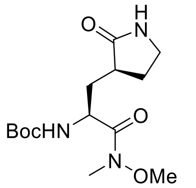

***tert*-Butyl ((*S*)-1-((*S*)-2-oxopyrrolidin-3-yl)but-3-yn-2-yl)carbamate (24).** To a solution of amide **47** (500 mg, 1.59 mmol, 1.0 equiv.) in anhydrous tetrahydrofuran (0.25 M) was added a $LiAlH_4$ solution (4.0 M in diethyl ether; 1.67 mmol, 417 µL, 1.2 equiv.) dropwise at -78 °C under nitrogen atmosphere. The resultant mixture was stirred for 1 h at -78 °C under nitrogen atmosphere. Then, the reaction was quenched at -78 °C by addition of a saturated aqueous NH_4Cl solution and warmed to 0 °C. The mixture was extracted with ethyl acetate (three times) and the combined organic extracts were dried over anhydrous Na_2SO_4, filtered, and evaporated to afford the crude aldehyde **9** (380 mg, 93%), which was used in the next step without further purification. Characteristic analytical data of aldehyde (**9**): ^{1}H NMR (400 MHz, 300 K, $CDCl_3$): δ = 9.58 (s, 1H), 6.18−6.14 (m, 2H), 4.23−4.20 (m, 1H), 3.39−3.35 (m, 3H), 2.53−2.42 (m, 2H), 2.02−1.94 (m, 1H), 1.89−1.82 (m, 1H), 1.47 ppm (s, 9H); ^{13}C NMR (100 MHz, 300 K, $CDCl_3$): δ = 200.3, 179.8, 156.1, 80.0, 58.7, 40.4, 37.8, 30.4, 28.6, 28.3 ppm; HRMS (ESI): *m/z* calculated for $C_{12}H_{21}O_4N_2$ [M+H]$^+$: 257.1496, found: 257.1495.

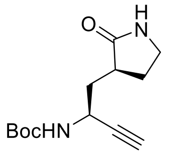

According to General Procedure C, alkyne **24** (124 mg, 43%) was obtained from aldehyde **9** (293 mg, 1.14 mmol), following column chromatography (10 g Sfär cartridge; 40 mL/min; initially, 100%$_{v/v}$ cyclohexane (2 CV), followed by a linear gradient (15 CV): 0%$_{v/v}$ → 60%$_{v/v}$ acetone in cyclohexane). ^{1}H NMR analysis indicated the that low levels of epimerization occurred α to the aldehyde; the diastereomer was not separated. Clear colorless oil; ^{1}H NMR (600 MHz, 300 K, DMSO-d_6): δ = 7.58 (s, 1H), 7.32 (brd, J = 8.6 Hz, 1H), 4.38 (dd, J = 14.9, 7.1 Hz, 1H), 3.16−3.09 (m, 3H), 2.24−2.15 (m, 2H), 1.96−1.92 (m, 1H), 1.67−1.61 (m, 1H), 1.51−1.47 (m, 1H), 1.37 ppm (s, 9H); ^{13}C NMR (150 MHz, 300 K, DMSO-d_6): δ = 178.1, 154.9, 84.7, 78.1, 72.9, 40.4, 39.2 (assigned using HSQC), 37.6, 36.8, 28.2, 27.7 ppm; IR (film): ṽ = 3287, 2980, 2110, 1690, 1522, 1459, 1367, 1252, 1167, 1031 cm^{-1}; HRMS (ESI): *m/z* calculated for $C_{13}H_{21}O_3N_2$ [M+H]$^+$: 253.1547, found: 253.1544.

(*S*)-3-((*S*)-2-Aminobut-3-yn-1-yl)pyrrolidin-2-one hydrochloride (11). According to General Procedure D, alkyne **11** (93 mg, app. quant.) was obtained from alkyne **24** (123 mg, 0.49 mmol) as a hydrochloride salt, which

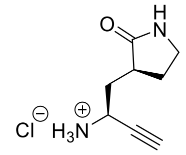

was used in the next step without further purification. Pale brown solid; ^{1}H NMR (600 MHz, 300 K, D_2O): δ = 4.43−4.40 (m, 1H), 3.44−3.37 (m, 2H), 3.06 (dd, J = 2.3, 0.9 Hz, 1H), 2.83−2.78 (m, 1H), 2.49−2.44 (m, 1H), 2.25−2.21 (m, 1H), 2.04 (ddd, J = 14.3, 8.0, 6.4 Hz, 1H), 1.98−1.93 ppm (m, 1H); ^{13}C NMR (150 MHz, 300 K, D_2O): δ = 181.3, 77.5, 77.2, 41.7, 40.8, 38.9, 34.2, 27.3 ppm; IR (film): ṽ = 3659, 3217, 2981, 2887, 2119, 1666, 1613, 1494, 1459, 1384, 1302, 1276, 1262, 1167, 1087, 1053 cm^{-1}; HRMS (ESI): *m/z* calculated for $C_8H_{13}ON_2$ [M–Cl]$^+$: 153.1022, found: 153.1020.

(1*R*,2*S*,5*S*)-3-((*S*)-3,3-Dimethyl-2-(2,2,2-trifluoroacetamido)butanoyl)-6,6-dimethyl-*N*-((*S*)-1-((*S*)-2-oxopyrrolidin-3-yl)but-3-yn-2-yl)-3-azabicyclo[3.1.0]hexane-2-carboxamide (13). According to General

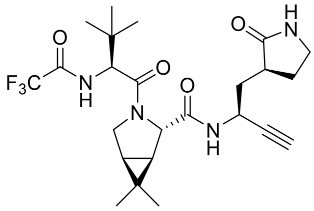

Procedure E, nirmatrelvir alkyne derivative **13** (69 mg, 17%) was obtained from alkyne **11** (150 mg, 0.80 mmol) and carboxylic acid **12**[8] (348 mg, 0.95 mmol), following HPLC purification (ACE 10 AQ 250 × 21.2 mm column; 20 mL/min; using a linear gradient of 2%$_{v/v}$ → 67%$_{v/v}$ acetonitrile (+0.1%$_{v/v}$ formic acid) in water (+0.1%$_{v/v}$ formic acid) over 30 min; t_R = 24.5 min). White amorphous solid; ^{1}H NMR (600 MHz, 300 K, DMSO-*d$_6$*): δ = 9.38 (d, J = 8.4 Hz, 1H), 8.56 (d, J = 9.0 Hz, 1H), 7.53 (s, 1H), 4.68−4.64 (m, 1H), 4.40 (d, J = 8.3 Hz, 1H), 4.17 (s, 1H), 3.89 (dd, J = 10.3, 5.5 Hz, 1H), 3.67 (d, J = 10.4 Hz, 1H), 3.20 (d, J = 2.3 Hz, 1H), 3.11−3.08 (m, 1H), 3.01−2.97 (m, 1H), 2.40−2.34 (m, 1H), 2.14−2.10 (m, 1H), 2.06−2.02 (m, 1H), 1.64−1.57 (m, 1H), 1.52 (dd, J = 7.5, 5.6 Hz, 1H), 1.49−1.44 (m, 1H), 1.24 (d, J = 7.7 Hz, 1H), 1.01 (s, 3H), 0.97 (s, 9H), 0.83 ppm (s, 3H); ^{13}C NMR (150 MHz, 300 K, DMSO-*d$_6$*): δ = 178.2, 170.0, 167.3, 156.9 (q, J = 37.1 Hz), 115.8 (q, J = 287.2 Hz), 84.2, 72.9, 60.1, 58.2, 47.7, 39.4 (assigned using HSQC), 37.8, 37.5, 37.0, 34.6, 30.5, 27.4, 27.2, 26.3, 25.8, 18.7, 12.4 ppm; ^{19}F NMR (565 MHz, 300 K, DMSO-*d$_6$*): δ = –72.9 ppm (s, 3F); IR (film): ṽ = 3274, 3056, 2930, 2874, 1670, 1541, 1507, 1457, 1438, 1353, 1265, 1230, 1197, 1162, 1112, 1084, 1060, 1012 cm^{-1}; HRMS (ESI): *m/z* calculated for $C_{24}H_{34}O_4N_4F_3$ [M+H]$^+$: 499.2527, found: 499.2511.

***tert*-Butyl (*S*)-(1-(methoxy(methyl)amino)-4-(methylthio)-1-oxobutan-2-yl)carbamate (48).** According to General Procedure B, amide **48** (594 mg, 99%) was obtained from commercially-sourced *N*-(*tert*-

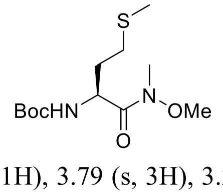

butoxycarbonyl)-*L*-methionine (**38**) (500 mg, 2.01 mmol), following column chromatography (25 g Sfär cartridge; 80 mL/min; initially, 100%$_{v/v}$ cyclohexane (2 CV), followed by a linear gradient (15 CV): 0%$_{v/v}$ → 40%$_{v/v}$ acetone in cyclohexane). Clear colorless oil; ^{1}H NMR (400 MHz, 300 K, $CDCl_3$): δ = 5.24 (brd, J = 8.0 Hz, 1H), 4.80 (brs, 1H), 3.79 (s, 3H), 3.22 (s, 3H), 2.59−2.53 (m, 2H), 2.11−2.10 (m, 3H), 2.05−1.98 (m, 1H), 1.85−1.78 (m, 1H), 1.44 ppm (s, 9H); ^{13}C NMR (100 MHz, 300 K, $CDCl_3$): δ = 172.5, 155.5, 79.6, 61.6, 49.8, 32.4, 32.1, 30.1, 28.3, 15.4 ppm; IR (film): ṽ = 3660, 3321, 2980, 1711, 1664, 1512, 1442, 1391, 1367, 1250, 1169, 1047, 1025 cm^{-1}; HRMS (ESI): *m/z* calculated for $C_{12}H_{25}O_4N_2S$ [M+H]$^+$: 293.1530, found: 293.1524.

***tert*-Butyl (*S*)-(5-(methylthio)pent-1-yn-3-yl)carbamate (49).** To a solution of amide **48** (300 mg, 1.03 mmol, 1.0 equiv.) in anhydrous tetrahydrofuran (0.25 M) was added a $LiAlH_4$ solution (4.0 M in diethyl ether; 1.28 mmol,

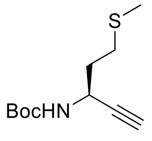

321 μL, 1.25 equiv.) dropwise at 0 °C under nitrogen atmosphere. The resultant mixture was stirred for 1 h at 0 °C under nitrogen atmosphere. Then, the reaction was quenched at 0 °C by addition of a 5%$_{m/v}$ aqueous $NaHSO_4$ solution; the mixture was diluted with ethyl acetate, and sequentially washed with 55%$_{m/v}$ aqueous $NaHSO_4$ solution (twice), 5%$_{m/v}$ aqueous $NaHCO_3$ solution (twice), and brine. The organic phase was dried over anhydrous Na_2SO_4, filtered, and evaporated to afford

the crude aldehyde **39** (226 mg, 94%), which was used in the next step without further purification. Characteristic analytical data of aldehyde (**39**): ^{1}H NMR (600 MHz, 300 K, $CDCl_3$): δ = 9.66 (s, 1H), 5.22 (brs, 1H), 4.35−4.32 (m, 1H), 2.62−2.54 (m, 2H), 2.27−2.24 (m, 1H), 2.10 (s, 3H), 1.98−1.93 (m, 1H), 1.47 ppm (s, 9H); ^{13}C NMR (150 MHz, 300 K, $CDCl_3$): δ = 199.0, 155.5, 80.3, 59.0, 29.8, 28.7, 28.2, 15.4 ppm; HRMS (ESI): *m/z* calculated for $C_{10}H_{20}O_3NS$ $[M+H]^+$: 234.1158, found: 234.1154.

According to General Procedure C, alkyne **49** (105 mg, 47%) was obtained from aldehyde **39** (224 mg, 0.96 mmol), following column chromatography (10 g Sfär cartridge; 40 mL/min; initially, 100%$_{v/v}$ cyclohexane (2 CV), followed by a linear gradient (15 CV): 0%$_{v/v}$ → 20%$_{v/v}$ acetone in cyclohexane). Clear colorless oil; ^{1}H NMR (400 MHz, 300 K, $CDCl_3$): δ = 4.82 (brs, 1H), 4.58 (brd, *J* = 4.8 Hz, 1H), 2.69−2.58 (m, 2H), 2.32 (d, *J* = 2.3 Hz, 1H), 2.13 (s, 3H), 2.03−1.90 (m, 2H), 1.47 ppm (s, 9H); ^{13}C NMR (100 MHz, 300 K, $CDCl_3$): δ = 154.7, 83.8, 82.7, 71.7, 42.1, 35.3, 30.0, 28.3, 15.5 ppm; IR (film): ṽ = 3659, 3240, 2981, 2890, 1687, 1490, 1460, 1444, 1379, 1308, 1282, 1250, 1166, 1071, 1028 cm^{-1}; HRMS (ESI): *m/z* calculated for $C_{11}H_{20}O_2NS$ $[M+H]^+$: 230.1209, found: 230.1207.

(*S*)-5-(Methylthio)pent-1-yn-3-amine hydrochloride (40). According to General Procedure D, alkyne **40** (64 mg, app. quant.) was obtained from alkyne **49** (88 mg, 0.38 mmol) as a hydrochloride salt, which was used in the next step without further purification. Brown solid; ^{1}H NMR (600 MHz, 300 K, DMSO-d_6): δ = 8.56 (brs, 3H), 4.15 (brs, 1H), 3.76 (d, *J* = 2.2 Hz, 1H), 2.64 (ddd, *J* = 13.5, 8.5, 5.0 Hz, 1H), 2.57−2.52 (m, 1H), 2.07−1.99 (m, 1H), 2.06 (s, 3H), 1.96−1.87 ppm (m, 1H); ^{13}C NMR (150 MHz, 300 K, DMSO-d_6): δ = 79.0, 78.7, 40.9, 32.1, 28.7, 14.4 ppm; IR (film): ṽ = 3659, 3401, 3268, 2981, 2888, 2125, 1515, 1407, 1372, 1159 cm^{-1}; HRMS (ESI): *m/z* calculated for $C_6H_{12}NS$ $[M–Cl]^+$: 130.0685, found: 130.0683.

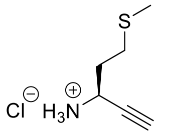

(1*R*,2*S*,5*S*)-3-((*S*)-3,3-Dimethyl-2-(2,2,2-trifluoroacetamido)butanoyl)-6,6-dimethyl-*N*-((*S*)-5-(methylthio)pent-1-yn-3-yl)-3-azabicyclo[3.1.0]hexane-2-carboxamide (14). According to General Procedure E, nirmatrelvir alkyne derivative **14** (19 mg, 17%) was obtained from alkyne **40** (40 mg, 0.24 mmol) and carboxylic acid **12**[8] (120 mg, 0.29 mmol), following HPLC purification (ACE 10 AQ 250 × 21.2 mm column; 20 mL/min; using a linear gradient of 2%$_{v/v}$ → 64%$_{v/v}$ acetonitrile (+0.1%$_{v/v}$ formic acid) in water (+0.1%$_{v/v}$ formic acid) over 55 min; t_R = 47.7 min). ^{1}H and ^{13}C NMR analysis indicated the presence of two rotamers, their relative ratio depends on the solvent used for NMR analysis; NMR data are given for the major rotamer. The occurrence of rotamers during NMR analysis of nirmatrelvir and derivatives has been reported[8]. White amorphous solid; ^{1}H NMR (600 MHz, 300 K, DMSO-d_6): δ = 9.32 (d, *J* = 9.4 Hz, 1H), 8.52 (d, *J* = 8.5 Hz, 1H), 4.68−4.64 (m, 1H), 4.37 (d, *J* = 7.9 Hz, 1H), 4.18 (s, 1H), 3.87 (dd, *J* = 10.3, 5.5 Hz, 1H), 3.68 (d, *J* = 10.4 Hz, 1H), 3.22 (d, *J* = 2.3 Hz, 1H), 2.55−2.51 (m, 1H), 2.47−2.43 (m, 1H), 2.01 (s, 3H), 1.86−1.76 (m, 2H), 1.52 (dd, *J* = 7.5, 5.6 Hz, 1H), 1.24 (d, *J* = 7.6 Hz, 1H), 1.01 (s, 3H), 0.97 (s, 9H), 0.83 ppm (s, 3H); ^{13}C NMR (150 MHz, 300 K, DMSO-d_6): δ = 170.0, 167.2, 156.9 (q, *J* = 36.7 Hz), 115.8 (q, *J* = 287.6 Hz), 83.5, 73.3, 60.0, 58.3, 47.6, 39.4 (assigned using HSQC), 35.1, 34.6, 30.5, 29.1, 27.2, 26.3, 25.8, 18.7, 14.6, 12.4 ppm; ^{19}F NMR (565 MHz, 300 K, DMSO-d_6): δ = –72.9 ppm (s, 3F); IR (film): ṽ = 3658, 3304, 2980, 2886, 1721, 1679, 1632, 1531, 1441, 1373, 1357, 1301, 1213, 1175, 1086 cm^{-1}; HRMS (ESI): *m/z* calculated for $C_{22}H_{33}O_3N_3F_3S$ $[M+H]^+$: 476.2189, found: 476.2177.

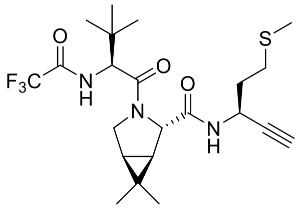

(1*R*,2*S*,5*S*)-*N*-((*S*)-1-Amino-4-(methylthio)-1-oxobutan-2-yl)-3-((*S*)-3,3-dimethyl-2-(2,2,2-trifluoroacetamido)butanoyl)-6,6-dimethyl-3-azabicyclo[3.1.0]hexane-2-carboxamide (50). According to

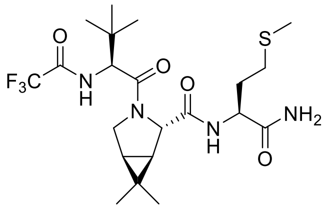

General Procedure E, amide **50** (43 mg, 36%) was obtained from commercially-sourced *L*-methionine amide (**41**; 131 mg, 0.88 mmol) and carboxylic acid **12**[8] (387 mg, 1.06 mmol), following HPLC purification (ACE 10 AQ 250 × 21.2 mm column; 20 mL/min; using a linear gradient of 2%$_{v/v}$ → 98%$_{v/v}$ acetonitrile (+0.1%$_{v/v}$ formic acid) in water (+0.1%$_{v/v}$ formic acid) over 32 min; t_R = 19.4 min). ^{1}H and ^{13}C NMR analysis indicated the presence of two rotamers, their relative ratio depends on the solvent used for NMR analysis; NMR data are given for the major rotamer. The occurrence of rotamers during NMR analysis of nirmatrelvir and derivatives has been reported[8]. White amorphous solid; ^{1}H NMR (600 MHz, 300 K, DMSO-*d$_6$*): δ = 9.33 (d, *J* = 8.0 Hz, 1H), 8.18 (d, *J* = 8.2 Hz, 1H), 7.30 (s, 1H), 7.02 (s, 1H), 4.38 (d, *J* = 8.0 Hz, 1H), 4.29−4.25 (m, 1H), 4.28 (s, 1H), 3.85 (dd, *J* = 10.3, 5.4 Hz, 1H), 3.68 (d, *J* = 10.4 Hz, 1H), 2.54−2.50 (m, 1H), 2.41−2.36 (m, 1H), 2.01 (s, 3H), 1.95−1.89 (m, 1H), 1.76−1.70 (m, 1H), 1.47 (dd, *J* = 7.5, 5.5 Hz, 1H), 1.40 (d, *J* = 7.7 Hz, 1H), 1.00 (s, 3H), 0.97 (s, 9H), 0.83 ppm (s, 3H); ^{13}C NMR (150 MHz, 300 K, DMSO-*d$_6$*): δ = 173.1, 170.7, 167.2, 156.8 (q, *J* = 37.0 Hz), 115.8 (q, *J* = 287.7 Hz), 60.1, 58.3, 51.7, 47.7, 34.6, 32.1, 30.5, 29.6, 27.0, 26.3, 25.9, 18.6, 14.7, 12.4 ppm; HRMS (ESI): *m/z* calculated for $C_{21}H_{34}O_4N_4F_3S$ [M+H]$^+$: 495.2245, found: 495.2247.

(1*R*,2*S*,5*S*)-*N*-((*S*)-1-Cyano-3-(methylthio)propyl)-3-((*S*)-3,3-dimethyl-2-(2,2,2-trifluoroacetamido)butanoyl)-6,6-dimethyl-3-azabicyclo[3.1.0]hexane-2-carboxamide (15). According to

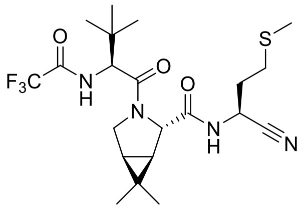

General Procedure F, nitrile **15** (41 mg, 13%) was obtained from amide **50** (336 mg, 0.68 mmol), following HPLC purification (ACE 10 AQ 250 × 21.2 mm column; 20 mL/min; using a linear gradient of 2%$_{v/v}$ → 98%$_{v/v}$ acetonitrile (+0.1%$_{v/v}$ formic acid) in water (+0.1%$_{v/v}$ formic acid) over 32 min; t_R = 22.8 min). ^{1}H and ^{13}C NMR analysis indicated the presence of two rotamers, their relative ratio depends on the solvent used for NMR analysis; NMR data are given for the major rotamer. The occurrence of rotamers during NMR analysis of nirmatrelvir and derivatives has been reported[8]. White amorphous solid; ^{1}H NMR (600 MHz, 300 K, DMSO-*d$_6$*): δ = 9.34 (d, *J* = 8.1 Hz, 1H), 8.93 (d, *J* = 8.0 Hz, 1H), 4.91 (td, *J* = 8.4, 5.7 Hz, 1H), 4.39 (d, *J* = 8.1 Hz, 1H), 4.17 (s, 1H), 3.88 (dd, *J* = 10.4, 5.5 Hz, 1H), 3.69 (d, *J* = 10.4 Hz, 1H), 2.58−2.54 (m, 1H), 2.47−2.45 (m, 1H), 2.05−2.02 (m, 1H), 2.03 (s, 3H), 2.00−1.94 (m, 1H), 1.56 (dd, *J* = 7.4, 5.6 Hz, 1H), 1.30 (d, *J* = 7.6 Hz, 1H), 1.02 (s, 3H), 0.97 (s, 9H), 0.84 ppm (s, 3H); ^{13}C NMR (150 MHz, 300 K, DMSO-*d$_6$*): δ = 170.8, 167.4, 156.9 (q, *J* = 36.9 Hz), 119.2, 115.8 (q, *J* = 287.8 Hz), 60.0, 58.3, 47.6, 38.7, 34.6, 31.8, 30.3, 28.5, 27.3, 26.3, 25.8, 18.8, 14.5, 12.3 ppm; ^{19}F NMR (565 MHz, 300 K, DMSO-*d$_6$*): δ = –72.9 ppm (s, 3F); HRMS (ESI): *m/z* calculated for $C_{21}H_{32}O_3N_4F_3S$ [M+H]$^+$: 477.2142, found: 477.2139.

(1*R*,2*S*,5*S*)-6,6-Dimethyl-*N*-((*S*)-1-((*S*)-2-oxopyrrolidin-3-yl)but-3-yn-2-yl)-3-(2-(4-(trifluoromethoxy)phenoxy)acetyl)-3-azabicyclo[3.1.0]hexane-2-carboxamide (18). According to General

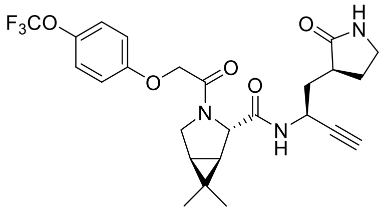

Procedure E, MI-09[13] alkyne derivative **18** (19 mg, 14%) was obtained from alkyne **11** (50 mg, 0.27 mmol) and carboxylic acid **16**[13] (119 mg, 0.32 mmol), following HPLC purification (ACE 10 AQ 250 × 21.2 mm column; 20 mL/min; using a linear gradient of 2%$_{v/v}$ → 65%$_{v/v}$ acetonitrile (+0.1%$_{v/v}$ formic acid) in water (+0.1%$_{v/v}$ formic acid) over 33 min; t_R = 28.8 min). ^{1}H and ^{13}C NMR analysis indicated the presence of two rotamers, their relative ratio depends on the solvent used for NMR analysis; NMR data are given for the major rotamer. The occurrence of rotamers during NMR analysis of nirmatrelvir and derivatives has been reported.[8] White amorphous solid; ^{1}H NMR (600 MHz, 300 K, DMSO-*d$_6$*): δ = 8.46 (d, *J* = 8.7 Hz, 1H), 7.50 (s, 1H),

7.23−7.22 (m, 2H), 6.93−6.92 (m, 2H), 4.84 (d, J = 15.7 Hz, 1H), 4.69 (d, J = 15.6 Hz, 1H), 4.67−4.64 (m, 1H), 4.12 (s, 1H), 3.83 (dd, J = 10.4, 5.4 Hz, 1H), 3.51 (d, J = 10.3 Hz, 1H), 3.21 (d, J = 2.3 Hz, 1H), 2.97−2.94 (m, 1H), 2.80−2.76 (m, 1H), 2.30−2.25 (m, 1H), 2.04−1.97 (m, 2H), 1.57−1.51 (m, 2H), 1.47 (ddd, J = 13.7, 10.5, 5.4 Hz, 1H), 1.25 (d, J = 7.6 Hz, 1H), 1.02 (s, 3H), 0.89 ppm (s, 3H); ^{13}C NMR (150 MHz, 300 K, DMSO-d_6): δ = 178.1, 169.9, 165.4, 156.9, 141.9, 122.2, 120.2 (q, J = 255.0 Hz), 115.8, 84.2, 73.0, 66.0, 60.2, 45.5, 39.4 (assigned using HSQC), 38.2, 37.3, 36.9, 30.5, 27.6, 27.1, 25.9, 18.7, 12.7 ppm; ^{19}F NMR (565 MHz, 300 K, DMSO-d_6): δ = –57.3 ppm (s, 3F); IR (film): ṽ = 3275, 2930, 1663, 1541, 1507, 1458, 1437, 1376, 1351, 1264, 1232, 1196, 1162, 1111, 1083, 1011 cm^{-1}; HRMS (ESI): m/z calculated for $C_{25}H_{29}O_5N_3F_3$ [M+H]$^+$: 508.2054, found: 508.2040.

(1*R*,2*S*,5*S*)-6,6-Dimethyl-*N*-((*S*)-1-((*S*)-2-oxopyrrolidin-3-yl)but-3-yn-2-yl)-3-(2-(4-(trifluoromethyl)phenoxy)acetyl)-3-azabicyclo[3.1.0]hexane-2-carboxamide (19). According to General Procedure E, MI-09[13] alkyne derivative **19** (14 mg, 13%) was obtained from alkyne **11** (35 mg, 0.19 mmol) and carboxylic acid **17** (80 mg, 0.22 mmol), following HPLC purification (ACE 10 AQ 250 × 21.2 mm column; 20 mL/min; using a linear gradient of 2%$_{v/v}$ → 65%$_{v/v}$ acetonitrile (+0.1%$_{v/v}$ formic acid) in water (+0.1%$_{v/v}$ formic acid) over 30 min; t_R = 16.3 min). ^{1}H and ^{13}C NMR analysis indicated the presence of two rotamers, their relative ratio depends on the solvent used for NMR analysis; NMR data are given for the major rotamer. The occurrence of rotamers during NMR analysis of nirmatrelvir and derivatives has been reported[8]. White amorphous solid; ^{1}H NMR (600 MHz, 300 K, CD_3OD): δ = 7.57−7.55 (m, 2H), 7.06−7.04 (m, 2H), 4.82 (d, J = 5.1 Hz, 1H), 4.81−4.78 (m, 1H), 4.60 (s, 2H), 4.29 (s, 1H) 3.97 (d, J = 10.4, 5.5 Hz, 1H), 3.62 (d, J = 10.4 Hz, 1H), 3.51 (d, J = 10.3 Hz, 1H), 3.11−3.06 (m, 1H), 2.92−2.86 (m, 1H), 2.73 (d, J = 2.3 Hz, 1H), 2.58−2.50 (m, 1H), 2.24−2.10 (m, 2H), 1.69−1.62 (m, 3H), 1.42 (d, J = 7.6 Hz, 1H), 1.11 (s, 3H), 1.00 ppm (s, 3H); ^{13}C NMR (150 MHz, 300 K, DMSO-d_6): δ = 178.1, 169.9, 165.2, 160.9, 126.6 (q, J = 3.4 Hz), 124.5 (q, J = 271.1 Hz), 121.3 (q, J = 31.9 Hz), 115.1, 84.2, 72.9, 65.6, 60.2, 45.5, 39.4 (assigned using HSQC), 38.1, 37.2, 36.9, 30.5, 27.5, 27.1, 25.9, 18.8, 12.7 ppm; ^{19}F NMR (565 MHz, 300 K, DMSO-d_6): δ = –59.8 ppm (s, 3F); IR (film): ṽ = 3275, 3055, 2944, 1663, 1616, 1593, 1541, 1520, 1457, 1435, 1329, 1314, 1258, 1237, 1184, 1162, 1113, 1064, 1042, 1011 cm^{-1}; HRMS (ESI): m/z calculated for $C_{25}H_{29}O_4N_3F_3$ [M+H]$^+$: 492.2105, found: 492.2091.

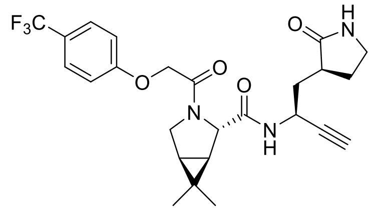

(1*R*,2*S*,5*S*)-*N*-((*S*)-1-Amino-1-oxo-3-((*S*)-2-oxopyrrolidin-3-yl)propan-2-yl)-6,6-dimethyl-3-(2-(4-(trifluoromethoxy)phenoxy)acetyl)-3-azabicyclo[3.1.0]hexane-2-carboxamide (51). According to General Procedure E, MI-09[13]-derived amide **51** (48 mg, 17%) was obtained from hydrochloride **43**[8] (133 mg, 0.64 mmol) and carboxylic acid **16**[13] (200 mg, 0.54 mmol) (Supporting Figure S4), following HPLC purification (ACE 10 AQ 250 × 21.2 mm column; 20 mL/min; using a linear gradient of 2%$_{v/v}$ → 98%$_{v/v}$ acetonitrile (+0.1%$_{v/v}$ formic acid) in water (+0.1%$_{v/v}$ formic acid) over 32 min; t_R = 18.8 min). ^{1}H and ^{13}C NMR analysis indicated the presence of two rotamers, their relative ratio depends on the solvent used for NMR analysis; NMR data are given for the major rotamer. The occurrence of rotamers during NMR analysis of nirmatrelvir and derivatives has been reported[8]. White amorphous solid; ^{1}H NMR (600 MHz, 300 K, DMSO-d_6): δ = 8.27 (d, J = 8.4 Hz, 1H), 7.55 (s, 1H), 7.24−7.22 (m, 2H), 7.15 (s, 1H), 7.02 (s, 1H), 6.94−6.93 (m, 2H), 4.86 (d, J = 15.7 Hz, 1H), 4.69 (d, J = 15.6 Hz, 1H), 4.24−4.20 (m, 1H), 4.19 (s, 1H), 3.84 (dd, J = 10.4, 5.4 Hz, 1H), 3.52 (d, J = 10.4 Hz, 1H), 3.00−2.97 (m, 1H), 2.85−2.82 (m, 1H), 2.30−2.25 (m, 1H), 2.03−1.99 (m, 1H), 1.93−1.88 (m, 1H), 1.56−1.49 (m, 3H), 1.38 (d, J = 7.6 Hz, 1H), 1.02 (s, 3H), 0.89 ppm (s, 3H); ^{13}C NMR (150 MHz, 300 K, DMSO-d_6): δ = 178.7, 173.5, 170.6, 165.7, 156.9, 141.9, 122.2, 120.2 (q, J = 255.2 Hz), 115.8, 66.1, 60.5, 50.7, 45.6, 39.0 (assigned using HSQC), 37.6, 33.4, 30.4, 27.5, 27.0, 25.9, 18.7, 12.7 ppm; ^{19}F NMR (565 MHz, 300 K, DMSO-

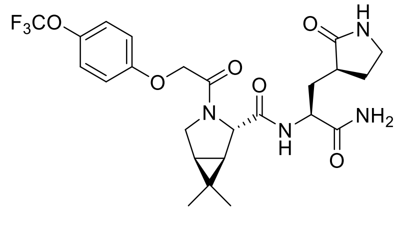

d_6): δ = –57.3 ppm (s, 3F); IR (film): ṽ = 3276, 3080, 2927, 2856, 1669, 1652, 1559, 1519, 1456, 1436, 1379, 1330, 1314, 1264, 1235, 1197, 1165, 1114, 1085, 1064, 1042, 1011 cm^{-1}; HRMS (ESI): *m/z* calculated for $C_{24}H_{30}O_6N_4F_3$ $[M+H]^+$: 527.2112, found: 527.2099.

(1*R*,2*S*,5*S*)-*N*-((*S*)-1-Cyano-2-((*S*)-2-oxopyrrolidin-3-yl)ethyl)-6,6-dimethyl-3-(2-(4-(trifluoromethoxy)phenoxy)acetyl)-3-azabicyclo[3.1.0]hexane-2-carboxamide (20). According to General Procedure F, MI-09[13] nitrile **20** (20 mg, 53%) was obtained from amide **51** (38 mg, 0.07 mmol), following HPLC purification (ACE 10 AQ 250 × 21.2 mm column; 20 mL/min; using a linear gradient of 2%$_{v/v}$ → 65%$_{v/v}$ acetonitrile (+0.1%$_{v/v}$ formic acid) in water (+0.1%$_{v/v}$ formic acid) over 30 min; t_R = 26.3 min). ^{1}H and ^{13}C NMR analysis indicated the presence of two rotamers, their relative ratio depends on the solvent used for NMR analysis; NMR data are given for the major rotamer. The occurrence of rotamers during NMR analysis of nirmatrelvir and derivatives has been reported[8]. White amorphous solid; ^{1}H NMR (600 MHz, 300 K, DMSO-d_6): δ = 8.87 (d, *J* = 8.1 Hz, 1H), 7.65 (s, 1H), 7.24−7.23 (m, 2H), 6.95−6.92 (m, 2H), 4.94 (ddd, *J* = 9.5, 8.1, 6.6 Hz, 1H), 4.89 (d, *J* = 15.7 Hz, 1H), 4.70 (d, *J* = 15.7 Hz, 1H), 4.11 (s, 1H), 3.85 (dd, *J* = 10.4, 5.5 Hz, 1H), 3.53 (d, *J* = 10.4 Hz, 1H), 3.04−3.01 (m, 1H), 2.91−2.87 (m, 1H), 2.34−2.27 (m, 1H), 2.13−2.00 (m, 2H), 1.75 (ddd, *J* = 13.6, 9.5, 6.4 Hz, 1H), 1.64−1.60 (m, 1H), 1.57 (dd, *J* = 7.4, 5.6 Hz, 1H), 1.31 (d, *J* = 7.5 Hz, 1H), 1.03 (s, 3H), 0.89 ppm (s, 3H); ^{13}C NMR (150 MHz, 300 K, DMSO-d_6): δ = 177.6, 170.7, 165.7, 156.8, 141.9, 122.2, 119.5, 118.5 (q, *J* = 254.3 Hz), 115.8, 65.9, 60.2, 45.5, 39.3 (assigned using HSQC), 38.1, 37.0, 33.6, 30.2, 27.2, 27.0, 25.8, 18.9, 12.6 ppm; ^{19}F NMR (565 MHz, 300 K, DMSO-d_6): δ = –57.3 ppm (s, 3F); IR (film): ṽ = 3275, 2980, 1671, 1558, 1541, 1507, 1458, 1436, 1376, 1351, 1264, 1232, 1196, 1162, 1110, 1083, 1059, 1012 cm^{-1}; HRMS (ESI): *m/z* calculated for $C_{24}H_{28}O_5N_4F_3$ $[M+H]^+$: 509.2006, found: 509.1990.

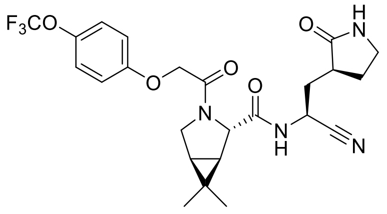

(1*R*,2*S*,5*S*)-*N*-((*S*)-1-Amino-1-oxo-3-((*S*)-2-oxopyrrolidin-3-yl)propan-2-yl)-6,6-dimethyl-3-(2-(4-(trifluoromethyl)phenoxy)acetyl)-3-azabicyclo[3.1.0]hexane-2-carboxamide (52). According to General Procedure E, MI-09[13]-derived amide **52** (121 mg, 41%) was obtained from hydrochloride **43**[8] (143 mg, 0.69 mmol) and carboxylic acid **17** (205 mg, 0.57 mmol) (Supporting Figure S4), following HPLC purification (ACE 10 AQ 250 × 21.2 mm column; 20 mL/min; using a linear gradient of 2%$_{v/v}$ → 98%$_{v/v}$ acetonitrile (+0.1%$_{v/v}$ formic acid) in water (+0.1%$_{v/v}$ formic acid) over 32 min; t_R = 18.3 min). ^{1}H and ^{13}C NMR analysis indicated the presence of two rotamers, their relative ratio depends on the solvent used for NMR analysis; NMR data are given for the major rotamer. The occurrence of rotamers during NMR analysis of nirmatrelvir and derivatives has been reported[8]. White amorphous solid; ^{1}H NMR (600 MHz, 300 K, DMSO-d_6): δ = 8.26 (d, *J* = 8.5 Hz, 1H), 7.59−7.57 (m, 2H), 7.52 (s, 1H), 7.15 (s, 1H), 7.03−7.02 (m, 3H), 4.94 (d, *J* = 15.8 Hz, 1H), 4.79 (d, *J* = 15.6 Hz, 1H), 4.24−4.21 (m, 1H), 4.20 (s, 1H), 3.86 (dd, *J* = 10.3, 5.5 Hz, 1H), 3.53 (d, *J* = 10.3 Hz, 1H), 2.95−2.92 (m, 1H), 2.76−2.71 (m, 1H), 2.28−2.22 (m, 1H), 1.99−1.96 (m, 1H), 1.92−1.88 (m, 1H), 1.53−1.51 (m, 3H), 1.39 (d, *J* = 7.5 Hz, 1H), 1.03 (s, 3H), 0.91 ppm (s, 3H); ^{13}C NMR (150 MHz, 300 K, DMSO-d_6): δ = 178.7, 173.5, 170.6, 165.4, 160.9, 126.6 (q, *J* = 3.6 Hz), 124.4 (q, *J* = 284.1 Hz), 121.2 (q, *J* = 32.0 Hz), 115.1, 65.7, 60.5, 50.6, 45.6, 39.3 (assigned using HSQC), 37.6, 33.4, 30.5, 27.5, 27.0, 25.9, 18.7, 12.8 ppm; ^{19}F NMR (565 MHz, 300 K, DMSO-d_6): δ = –59.8 ppm (s, 3F); IR (film): ṽ = 3276, 2979, 2883, 1651, 1617, 1594, 1542, 1519, 1456, 1436, 1379, 1329, 1314, 1266, 1235, 1186, 1164, 1114, 1064, 1042, 1011 cm^{-1}; HRMS (ESI): *m/z* calculated for $C_{24}H_{30}O_5N_4F_3$ $[M+H]^+$: 511.2163, found: 511.2152.

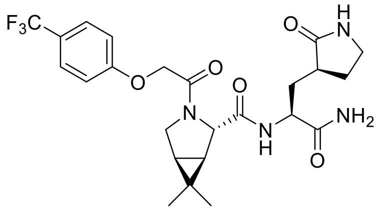

(1*R*,2*S*,5*S*)-*N*-((*S*)-1-Cyano-2-((*S*)-2-oxopyrrolidin-3-yl)ethyl)-6,6-dimethyl-3-(2-(4-(trifluoromethyl)phenoxy)acetyl)-3-azabicyclo[3.1.0]hexane-2-carboxamide (21). According to General Procedure F, nitrile **21** (61 mg, 58%) was obtained from amide **52** (110 mg, 0.22 mmol), following HPLC purification (ACE 10 AQ 250 × 21.2 mm column; 20 mL/min; using a linear gradient of $2\%_{v/v} \rightarrow 98\%_{v/v}$ acetonitrile (+$0.1\%_{v/v}$ formic acid) in water (+$0.1\%_{v/v}$ formic acid) over 32 min; t_R = 20.3 min). ^{1}H and ^{13}C NMR analysis indicated the presence of two rotamers, their relative ratio depends on the solvent used for NMR analysis; NMR data are given for the major rotamer. The occurrence of rotamers during NMR analysis of nirmatrelvir and derivatives has been reported[8]. White amorphous solid; ^{1}H NMR (600 MHz, 300 K, DMSO-d_6): δ = 8.87 (d, *J* = 8.2 Hz, 1H), 7.63 (s, 1H), 7.59 (d, *J* = 8.7 Hz, 2H), 7.03 (d, *J* = 8.7 Hz, 2H), 4.97−4.92 (m, 2H), 4.80 (d, *J* = 15.8 Hz, 1H), 4.11 (s, 1H), 3.87 (dd, *J* = 10.4, 5.5 Hz, 1H), 3.54 (d, *J* = 10.2 Hz, 1H), 3.00−2.97 (m, 1H), 2.84−2.80 (m, 1H), 2.32−2.26 (m, 1H), 2.10−2.06 (m, 1H), 2.01−1.97 (m, 1H), 1.74 (ddd, *J* = 13.6, 9.5, 6.3 Hz, 1H), 1.62−1.57 (m, 2H), 1.32 (d, *J* = 7.5 Hz, 1H), 1.03 (s, 3H), 0.90 ppm (s, 3H); ^{13}C NMR (150 MHz, 300 K, DMSO-d_6): δ = 177.5, 170.7, 165.4, 160.8, 126.6 (q, *J* = 3.3 Hz), 124.5 (q, *J* = 271.0 Hz), 121.3 (q, *J* = 31.9 Hz), 119.5, 115.1, 65.6, 60.2, 45.4, 39.3 (assigned using HSQC), 38.1, 36.9, 33.7, 30.2, 27.2, 27.0, 25.8, 18.9, 12.7 ppm; ^{19}F NMR (565 MHz, 300 K, DMSO-d_6): δ = −59.8 ppm (s, 3F); IR (film): ṽ = 3276, 2980, 2930, 1674, 1616, 1593, 1520, 1458, 1433, 1379, 1329, 1314, 1237, 1183, 1162, 1113, 1064, 1011 cm^{-1}; HRMS (ESI): *m/z* calculated for $C_{24}H_{28}O_4N_4F_3$ $[M+H]^+$: 493.2057, found: 493.2043.

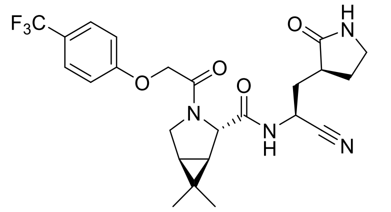

Benzyl ((*S*)-4-methyl-1-oxo-1-(((*S*)-1-((*S*)-2-oxopyrrolidin-3-yl)but-3-yn-2-yl)amino)pentan-2-yl)carbamate (23). According to General Procedure E, GC376 alkyne derivative **23** (31 mg, 43%) was obtained from alkyne **11** (45 mg, 0.24 mmol) and commercially-sourced *N*-(benzyloxycarbonyl)-L-leucine (76 mg, 0.29 mmol) as a 5:1 mixture of diastereomers, following HPLC purification (ACE 10 AQ 250 × 21.2 mm column; 20 mL/min; using a linear gradient of $2\%_{v/v} \rightarrow 98\%_{v/v}$ acetonitrile (+$0.1\%_{v/v}$ formic acid) in water (+$0.1\%_{v/v}$ formic acid) over 32 min; t_R = 19.1 min). White amorphous solid; NMR data are provided for the major diastereomer: ^{1}H NMR (600 MHz, 300 K, DMSO-d_6): δ = 8.37(d, *J* = 8.6 Hz, 1H), 7.57 (s, 1H), 7.39 (d, *J* = 8.1 Hz, 1H), 7.35−7.29 (m, 5H), 5.03 (d, *J* = 12.8 Hz, 1H), 4.98 (d, *J* = 12.7 Hz, 1H), 4.68−4.64 (m, 1H), 3.97 (ddd, *J* = 9.8, 8.2, 5.1 Hz, 1H), 3.17 (d, *J* = 2.3 Hz, 1H), 3.11−3.08 (m, 1H), 3.03−2.98 (m, 1H), 2.30−2.25 (m, 1H), 2.13−2.09 (m, 1H), 2.05−2.00 (m, 1H), 1.64−1.57 (m, 2H), 1.53−1.49 (m, 1H), 1.46−1.42 (m, 1H), 1.34 (ddd, *J* = 13.6, 8.6, 5.1 Hz, 1H), 0.87 (d, *J* = 6.6 Hz, 3H), 0.84 ppm (d, *J* = 6.4 Hz, 3H); ^{13}C NMR (150 MHz, 300 K, DMSO-d_6): δ = 178.1, 171.7, 155.9, 137.1, 128.3, 127.6, 127.5, 84.3, 72.8, 65.2, 53.0, 40.5, 39.4 (assigned using HSQC), 38.3, 37.4, 36.7, 27.6, 24.2, 23.0, 21.5 ppm; IR (film): ṽ = 3280, 3056, 2956, 2932, 2871, 1685, 1670, 1540, 1457, 1439, 1387, 1368, 1267, 1242, 1174, 1118, 1047, 1029 cm^{-1}; HRMS (ESI): *m/z* calculated for $C_{22}H_{30}O_4N_4$ $[M+H]^+$: 400.2231, found: 400.2220.

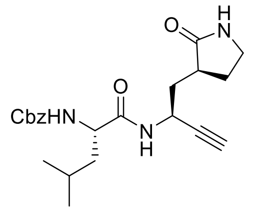

***tert*-Butyl ((*S*)-1-((*S*)-2-oxopyrrolidin-3-yl)-4-phenylbut-3-yn-2-yl)carbamate (25a).** According to General Procedure G, alkyne **25a** (62 mg, 46%) was obtained from alkyne **24** (104 mg, 0.41 mmol) and commercially-sourced iodobenzene (109 mg, 0.536 mmol), following column chromatography (10 g Sfär cartridge; 40 mL/min; initially, $100\%_{v/v}$ cyclohexane (2 CV), followed by a linear gradient (15 CV): $0\%_{v/v} \rightarrow 45\%_{v/v}$ acetone in cyclohexane). Pale yellow amorphous solid; ^{1}H NMR (600 MHz, 300 K, DMSO-d_6): δ = 7.61 (s, 1H), 7.43 (brd, *J* = 8.5 Hz, 1H), 7.40−7.38 (m, 2H), 7.37−7.35 (m, 3H), 4.64 (dd, *J* = 15.6, 7.8 Hz, 1H), 3.17−3.11 (m, 2H), 2.30−2.22 (m, 2H), 2.06−2.03 (m, 1H), 1.74−1.68 (m, 1H), 1.61 (ddd, *J* = 13.4, 8.7, 7.1 Hz, 1H), 1.39 ppm (s, 9H); ^{13}C NMR (150 MHz, 300 K, DMSO-d_6): δ = 178.0, 154.9, 131.3, 128.6, 128.4,

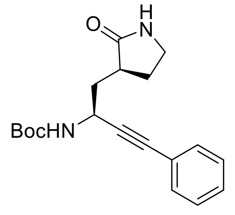

122.4, 90.5, 81.6, 78.2, 41.2, 39.3 (assigned using HSQC), 37.8, 37.1, 28.2, 27.7 ppm; IR (film): ṽ = 3660, 3301, 2981, 2889, 1692, 1514, 1491, 1442, 1391, 1368, 1296, 1251, 1167, 1070, 1026 cm^{-1}; HRMS (ESI): *m/z* calculated for $C_{19}H_{25}O_3N_2$ $[M+H]^+$: 329.1860, found: 329.1852.

(*S*)-3-((*S*)-2-Amino-4-phenylbut-3-yn-1-yl)pyrrolidin-2-one hydrochloride (26a). According to General Procedure D, alkyne **26a** (32 mg, app. quant.) was obtained from alkyne **25a** (40 mg, 0.12 mmol) as hydrochloride salt, which was used in the next step without further purification. Pale brown solid; ^{1}H NMR (600 MHz, 300 K, DMSO-*d₆*): δ = 8.61 (brs, 3H), 7.84 (s, 1H), 7.49−7.46 (m, 2H), 7.45−7.41 (m, 3H), 4.61 (dd, *J* = 8.8, 5.9 Hz, 1H), 3.21−3.17 (m, 2H), 2.56−2.51 (m, 1H), 2.36−2.31 (m, 1H), 2.21−2.16 (m, 1H), 1.85−1.80 (m, 1H), 1.79−1.74 ppm (m, 1H); ^{13}C NMR (150 MHz, 300 K, DMSO-d_6): δ = 177.9, 131.5, 129.4, 128.9, 121.0, 86.0, 85.5, 41.7, 39.5 (assigned using HSQC), 38.1, 35.1, 28.0 ppm; IR (film): ṽ = 3659, 3389, 3235, 2981, 2890, 2069, 1679, 1492, 1380, 1252, 1168, 1073, 1049 cm^{-1}; HRMS (ESI): *m/z* calculated for $C_{14}H_{17}ON_2$ $[M–Cl]^+$: 229.1335, found: 229.1331.

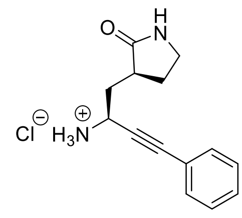

(1*R*,2*S*,5*S*)-3-((*S*)-3,3-Dimethyl-2-(2,2,2-trifluoroacetamido)butanoyl)-6,6-dimethyl-*N*-((*S*)-1-((*S*)-2-oxopyrrolidin-3-yl)-4-phenylbut-3-yn-2-yl)-3-azabicyclo[3.1.0]hexane-2-carboxamide (27a). According to General Procedure E, nirmatrelvir alkyne derivative **27a** (10 mg, 17%) was obtained from alkyne **26a** (25 mg, 0.10 mmol) and carboxylic acid **12**[8] (44 mg, 0.12 mmol), following HPLC purification (ACE 10 AQ 250 × 21.2 mm column; 20 mL/min; using a linear gradient of 2$\%_{v/v}$ → 75$\%_{v/v}$ acetonitrile (+0.1$\%_{v/v}$ formic acid) in water (+0.1$\%_{v/v}$ formic acid) over 40 min; t_R = 33.0 min). White amorphous solid; ^{1}H NMR (600 MHz, 300 K, DMSO-d_6): δ = 9.39 (brs, 1H), 8.65 (d, *J* = 9.0 Hz, 1H), 7.55 (s, 1H), 7.42−7.40 (m, 2H), 7.39−7.36 (m, 3H), 4.92 (ddd, *J* = 10.8, 9.1, 4.3 Hz, 1H), 4.42 (brs, 1H), 4.19 (s, 1H), 3.91 (dd, *J* = 10.2, 5.5 Hz, 1H), 3.68 (d, *J* = 10.4 Hz, 1H), 3.14−3.11 (m, 1H), 3.04−3.00 (m, 1H), 2.44−2.41 (m, 1H), 2.20−2.12 (m, 2H), 1.71−1.65 (m, 1H), 1.59 (ddd, *J* = 13.5, 11.3, 4.2 Hz, 1H), 1.53 (dd, *J* = 7.4, 5.7 Hz, 1H), 1.30 (d, *J* = 7.7 Hz, 1H), 1.01 (s, 3H), 0.98 (s, 9H), 0.84 ppm (s, 3H); ^{13}C NMR (150 MHz, 300 K, DMSO-d_6): δ = 178.2, 170.0, 167.3, 156.9 (q, *J* = 37.9 Hz), 131.4, 128.7, 128.6, 122.2, 115.8 (q, *J* = 287.6 Hz), 90.0, 81.5, 60.2, 58.2, 47.7, 39.4 (assigned using HSQC), 38.5, 37.7, 37.2, 34.6, 30.5, 27.5, 27.2, 26.3, 25.8, 18.7, 12.4 ppm; ^{19}F NMR (565 MHz, 300 K, DMSO-d_6): δ = –72.9 ppm (s, 3F); IR (film): ṽ = 3275, 2980, 2970, 2885, 1685, 1636, 1541, 1508, 1490, 1456, 1441, 1397, 1373, 1298, 1267, 1212, 1178, 1159, 1055, 1028, 1007 cm^{-1}; HRMS (ESI): *m/z* calculated for $C_{30}H_{38}O_4N_4F_3$ $[M+H]^+$: 575.2840, found: 575.2822.

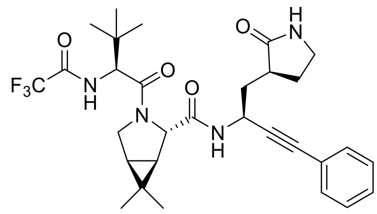

***tert*-Butyl ((*S*)-4-(4-nitrophenyl)-1-((*S*)-2-oxopyrrolidin-3-yl)but-3-yn-2-yl)carbamate (25b).** According to General Procedure G, alkyne **25b** (100 mg, 44%) was obtained from alkyne **24** (152 mg, 0.60 mmol) and commercially-sourced 1-iodo-4-nitrobenzene (195 mg, 0.78 mmol), following column chromatography (10 g Sfär cartridge; 40 mL/min; initially, 100$\%_{v/v}$ cyclohexane (2 CV), followed by a linear gradient (15 CV): 0$\%_{v/v}$ → 45$\%_{v/v}$ acetone in cyclohexane). Clear orange oil; ^{1}H NMR (600 MHz, 300 K, DMSO-d_6): δ = 8.21−8.20 (m, 2H), 7.68−7.65 (m, 2H), 7.62 (brs, 1H), 7.52 (brd, *J* = 8.5 Hz, 1H), 4.71 (dd, *J* = 15.3, 7.6 Hz, 1H), 3.18−3.11 (m, 2H), 2.34−2.29 (m, 1H), 2.26−2.21 (m, 1H), 2.11−2.04 (m, 1H), 1.75−1.63 (m, 2H), 1.40 ppm (s, 9H); ^{13}C NMR (150 MHz, 300 K, DMSO-d_6): δ = 178.0, 154.9, 146.8, 132.6, 129.2, 123.8, 95.9, 80.3, 78.4, 41.2, 39.3 (assigned using HSQC), 37.7, 36.8, 28.2, 27.6 ppm; IR (film): ṽ = 3659, 3262, 2981, 2918, 2850, 1692, 1594, 1518, 1492, 1473, 1462, 1390, 1367, 1344, 1252, 1165, 1108, 1071, 1013 cm^{-1}; HRMS (ESI): *m/z* calculated for $C_{19}H_{24}O_5N_3$ $[M+H]^+$: 374.1711, found:

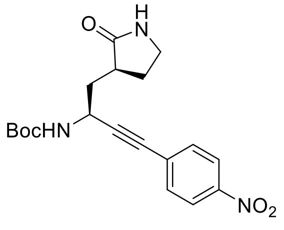

374.1702.

(*S*)-3-((*S*)-2-Amino-4-(4-nitrophenyl)but-3-yn-1-yl)pyrrolidin-2-one hydrochloride (26b). According to General Procedure D, alkyne **26b** (90 mg, app. quant.) was obtained from alkyne **25b** (100 mg, 0.27 mmol) as

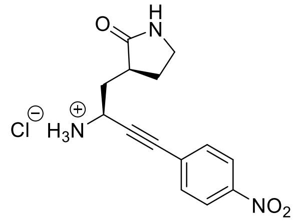

hydrochloride salt, which was used in the next step without further purification. Brown solid; ^{1}H NMR (600 MHz, 300 K, DMSO-d_6): δ = 8.79 (brs, 3H), 8.27−8.26 (m, 2H), 7.84 (s, 1H), 7.75−7.73 (m, 2H), 4.69 (dd, *J* = 8.8, 6.0 Hz, 1H), 3.21−3.15 (m, 2H), 2.59−2.54 (m, 1H), 2.36−2.31 (m, 1H), 2.23−2.18 (m, 1H), 1.87 (ddd, *J* = 13.6, 8.8, 6.8 Hz, 1H), 1.80−1.73 ppm (m, 1H); ^{13}C NMR (150 MHz, 300 K, DMSO-d_6): δ = 177.9, 147.4, 132.8, 127.8, 124.0, 90.4, 84.3, 41.5, 39.5 (assigned using HSQC), 38.0, 34.8, 27.9 ppm; IR (film): ṽ = 3395, 2981, 2889, 1706, 1689, 1594, 1519, 1491, 1460, 1381, 1345, 1305, 1282, 1256, 1173, 1119, 1081, 1011 cm^{-1}; HRMS (ESI): *m/z* calculated for $C_{14}H_{16}O_3N_3$ [M–Cl]$^+$: 274.1186, found: 274.1179.

(1*R*,2*S*,5*S*)-3-((*S*)-3,3-Dimethyl-2-(2,2,2-trifluoroacetamido)butanoyl)-6,6-dimethyl-*N*-((*S*)-4-(4-nitrophenyl)-1-((*S*)-2-oxopyrrolidin-3-yl)but-3-yn-2-yl)-3-azabicyclo[3.1.0]hexane-2-carboxamide (27b).

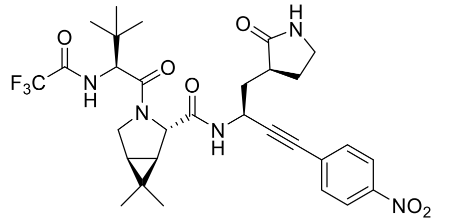

According to General Procedure E, nirmatrelvir alkyne derivative **27b** (20 mg, 11%) was obtained from alkyne **26b** (90 mg, 0.29 mmol) and carboxylic acid **12**[8] (127 mg, 0.35 mmol), following HPLC purification (ACE 10 AQ 250 × 21.2 mm column; 20 mL/min; using a linear gradient of 2%$_{v/v}$ → 75%$_{v/v}$ acetonitrile (+0.1%$_{v/v}$ formic acid) in water (+0.1%$_{v/v}$ formic acid) over 40 min; t_R = 31.5 min). Pale brown solid; ^{1}H NMR (600 MHz, 300 K, DMSO-d_6): δ = 9.40 (d, *J* = 8.5 Hz, 1H), 8.73 (d, *J* = 8.9 Hz, 1H), 8.23−8.21 (m, 2H), 7.70−7.68 (m, 2H), 7.57 (s, 1H), 4.98 (ddd, *J* = 10.8, 9.0, 4.4 Hz, 1H), 4.42 (d, *J* = 8.5 Hz, 1H), 4.19 (s, 1H), 3.91 (dd, *J* = 10.3, 5.5 Hz, 1H), 3.68 (d, *J* = 10.4 Hz, 1H), 3.14−3.11 (m, 1H), 3.05−3.00 (m, 1H), 2.47−2.42 (m, 1H), 2.19−2.15 (m, 2H), 1.72−1.61 (m, 2H), 1.54 (dd, *J* = 7.5, 5.6 Hz, 1H), 1.32 (d, *J* = 7.7 Hz, 1H), 1.01 (s, 3H), 0.98 (s, 9H), 0.84 ppm (s, 3H); ^{13}C NMR (150 MHz, 300 K, DMSO-d_6): δ = 178.1, 170.1, 167.3, 156.9 (q, *J* = 37.1 Hz), 146.8, 132.7, 129.0, 123.8, 115.8 (q, *J* = 288.0 Hz), 95.3, 80.1, 60.2, 58.2, 47.7, 39.4 (assigned using HSQC), 38.6, 37.3, 37.1, 34.6, 30.5, 27.4, 27.2, 26.3, 25.8, 18.7, 12.4 ppm; ^{19}F NMR (565 MHz, 300 K, DMSO-d_6): δ = –72.9 ppm (s, 3F); IR (film): ṽ = 3659, 3423, 2981, 2888, 1687, 1642, 1595, 1519, 1440, 1380, 1344, 1212, 1178, 1156, 1051, 1027, 1004 cm^{-1}; HRMS (ESI): *m/z* calculated for $C_{30}H_{37}O_6N_5F_3$ [M+H]$^+$: 620.2690, found: 620.2672.

(1*R*,2*S*,5*S*)-3-((*S*)-3,3-Dimethyl-2-(2,2,2-trifluoroacetamido)butanoyl)-6,6-dimethyl-*N*-((*S*)-5,5,5-trifluoro-1-((*S*)-2-oxopyrrolidin-3-yl)pent-3-yn-2-yl)-3-azabicyclo[3.1.0]hexane-2-carboxamide (28). A mixture of CuI

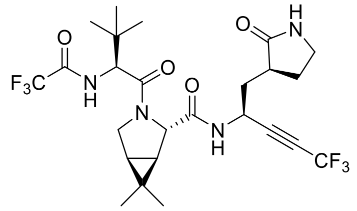

(12 mg, 0.06 mmol, 1.5 equiv.), K_2CO_3 (17 mg, 0.12 mmol, 3.0 equiv.), and *N*,*N*,*N'*,*N'*-tetramethylethylenediamine (TMEDA; 9 µL, 0.06 mmol, 1.5 equiv.) in anhydrous *N*,*N*-dimethylformamide (DMF; 200 µL) was stirred at room temperature under ambient atmosphere for 15 min during which it turned deep blue. Then, $TMSCF_3$ (12 µL, 0.08 mmol, 2.0 equiv.) was added to the mixture and the resulting deep green mixture was stirred for 5 min under air atmosphere, followed by cooling to 0 °C. An ice-cold solution of nirmatrelvir alkyne derivative **13** (20 mg, 0.04 mmol, 1.0 equiv.) and $TMSCF_3$ (12 µL, 0.08 mmol, 2.0 equiv.) in anhydrous DMF (200 µL) was added to the reaction mixture. The reaction mixture was stirred for 30 min at 0 °C under ambient atmosphere, and for 24 h at ambient temperature before being diluted with deionized water. The mixture was extracted with diethyl ether (three times); the combined organic layers were sequentially washed with water (three times) and brine, dried over

anhydrous Na_2SO_4, filtered, and evaporated. The crude product was purified using HPLC (ACE 10 AQ 250 × 21.2 mm column; 20 mL/min; using a linear gradient of 2%$_{v/v}$ → 98%$_{v/v}$ acetonitrile (+0.1%$_{v/v}$ formic acid) in water (+0.1%$_{v/v}$ formic acid) over 32 min; t_R = 22.5 min). Fractions containing pure **28** were lyophilized to afford the alkyne **28** (6 mg, 26%). ^{1}H and ^{13}C NMR analysis indicated the presence of two rotamers, their relative ratio depends on the solvent used for NMR analysis; NMR data are given for the major rotamer. The occurrence of rotamers during NMR analysis of nirmatrelvir and derivatives has been reported[8]. White amorphous solid; ^{1}H NMR (600 MHz, 300 K, DMSO-d_6): δ = 9.41 (d, J = 8.3 Hz, 1H), 7.63 (s, 1H), 4.91 (s, 1H), 4.44 (d, J = 8.3 Hz, 1H), 3.90 (dd, J = 10.6, 5.2 Hz, 1H), 3.88−3.81 (m, 3H), 3.11−3.08 (m, 2H), 2.78 (dd, J = 14.0, 3.5 Hz, 1H), 2.47−2.40 (m, 2H), 2.01−1.96 (m, 1H), 1.72−1.65 (m, 1H), 1.54 (dd, J = 7.4, 5.3 Hz, 1H), 1.44 (d, J = 7.6 Hz, 1H), 1.01 (s, 3H), 0.94 (s, 9H), 0.87 ppm (s, 3H); ^{13}C NMR (150 MHz, 300 K, DMSO-d_6): δ = 177.7, 167.8, 162.4, 157.0 (q, J = 37.1 Hz), 137.6, 137.3 (q, J = 3.3 Hz), 125.0 (q, J = 277.7 Hz), 115.9 (q, J = 287.7 Hz), 64.9, 58.7, 54.2, 47.4, 40.3, 34.5, 30.8, 29.0 (q, J = 31.4 Hz), 27.2, 26.8, 26.1, 25.8, 25.7(7), 19.0, 12.0 ppm; ^{19}F NMR (565 MHz, 300 K, DMSO-d_6): δ = –63.9 (m, 3F), –72.9 ppm (s, 3F); IR (film): ṽ = 3652, 3433, 2981, 2888, 1692, 1639, 1551, 1437, 1376, 1357, 1308, 1260, 1213, 1148, 1071 cm^{-1}; HRMS (ESI): *m/z* calculated for $C_{25}H_{33}O_4N_4F_6$ $[M+H]^+$: 567.2401, found: 567.2386.

6. References

1. Malla, T. R.; Tumber, A.; John, T.; Brewitz, L.; Strain-Damerell, C.; Owen, C. D.; Lukacik, P.; Chan, H. T. H.; Maheswaran, P.; Salah, E.; Duarte, F.; Yang, H.; Rao, Z.; Walsh, M. A.; Schofield, C. J., Mass spectrometry reveals potential of β-lactams as SARS-CoV-2 Mpro inhibitors. *Chem. Commun.* **2021,** *57* (12), 1430-1433.
2. Malla, T. R.; Brewitz, L.; Muntean, D.-G.; Aslam, H.; Owen, C. D.; Salah, E.; Tumber, A.; Lukacik, P.; Strain-Damerell, C.; Mikolajek, H.; Walsh, M. A.; Schofield, C. J., Penicillin derivatives inhibit the SARS-CoV-2 main protease by reaction with its nucleophilic cysteine. *J. Med. Chem.* **2022,** *65* (11), 7682-7696.
3. Brewitz, L.; Kamps, J. J. A. G.; Lukacik, P.; Strain-Damerell, C.; Zhao, Y.; Tumber, A.; Malla, T. R.; Orville, A. M.; Walsh, M. A.; Schofield, C. J., Mass spectrometric assays reveal discrepancies in inhibition profiles for the SARS-CoV-2 papain-like protease. *ChemMedChem* **2022,** *17*, e202200016.
4. Engel-Andreasen, J.; Wellhöfer, I.; Wich, K.; Olsen, C. A., Backbone-fluorinated 1,2,3-triazole-containing dipeptide surrogates. *J. Org. Chem.* **2017,** *82* (21), 11613-11619.
5. Nahm, S.; Weinreb, S. M., N-Methoxy-N-methylamides as effective acylating agents. *Tetrahedron Lett.* **1981,** *22* (39), 3815-3818.
6. Ohira, S., Methanolysis of dimethyl (1-diazo-2-oxopropyl) phosphonate: generation of dimethyl (diazomethyl) phosphonate and reaction with carbonyl compounds. *Synth. Commun.* **1989,** *19* (3-4), 561-564.
7. Müller, S.; Liepold, B.; Roth, G. J.; Bestmann, H. J., An improved one-pot procedure for the synthesis of alkynes from aldehydes. *Synlett* **1996,** *1996* (06), 521-522.
8. Owen, D. R.; Allerton, C. M. N.; Anderson, A. S.; Aschenbrenner, L.; Avery, M.; Berritt, S.; Boras, B.; Cardin, R. D.; Carlo, A.; Coffman, K. J.; Dantonio, A.; Di, L.; Eng, H.; Ferre, R.; Gajiwala, K. S.; Gibson, S. A.; Greasley, S. E.; Hurst, B. L.; Kadar, E. P.; Kalgutkar, A. S.; Lee, J. C.; Lee, J.; Liu, W.; Mason, S. W.; Noell, S.; Novak, J. J.; Obach, R. S.; Ogilvie, K.; Patel, N. C.; Pettersson, M.; Rai, D. K.; Reese, M. R.; Sammons, M. F.; Sathish, J. G.; Singh, R. S. P.; Steppan, C. M.; Stewart, A. E.; Tuttle, J. B.; Updyke, L.; Verhoest, P. R.; Wei, L.; Yang, Q.; Zhu, Y., An oral SARS-CoV-2 Mpro inhibitor clinical candidate for the treatment of COVID-19. *Science* **2021,** *374* (6575), 1586-1593.
9. El-Faham, A.; Albericio, F., COMU: a third generation of uronium-type coupling reagents. *J. Pept. Sci.* **2010,** *16* (1), 6-9.
10. Atkins Jr., G. M.; Burgess, E. M., The reactions of an N-sulfonylamine inner salt. *J. Am. Chem. Soc.* **1968,** *90* (17), 4744-4745.
11. Burgess, E. M.; Penton Jr., H. R.; Taylor, E. A., Thermal reactions of alkyl N-carbomethoxysulfamate esters. *J. Org. Chem.* **1973,** *38* (1), 26-31.
12. Zhang, J.-H.; Chung, T. D. Y.; Oldenburg, K. R., A simple statistical parameter for use in evaluation and validation of high throughput screening assays. *J. Biomol. Screen.* **1999,** *4* (2), 67-73.
13. Qiao, J.; Li, Y.-S.; Zeng, R.; Liu, F.-L.; Luo, R.-H.; Huang, C.; Wang, Y.-F.; Zhang, J.; Quan, B.; Shen, C.; Mao, X.; Liu, X.; Sun, W.; Yang, W.; Ni, X.; Wang, K.; Xu, L.; Duan, Z.-L.; Zou, Q.-C.; Zhang, H.-L.; Qu, W.; Long, Y.-H.-P.; Li, M.-H.; Yang, R.-C.; Liu, X.; You, J.; Zhou, Y.; Yao, R.; Li, W.-P.; Liu, J.-M.; Chen, P.; Liu, Y.; Lin, G.-F.; Yang, X.; Zou, J.; Li, L.; Hu, Y.; Lu, G.-W.; Li, W.-M.; Wei, Y.-Q.; Zheng, Y.-T.; Lei, J.; Yang, S., SARS-CoV-2 Mpro inhibitors with antiviral activity in a transgenic mouse model. *Science* **2021,** *371* (6536), 1374-1378.
14. Yang, K. S.; Leeuwon, S. Z.; Xu, S.; Liu, W. R., Evolutionary and structural insights about potential SARS-CoV-2 evasion of nirmatrelvir. *J. Med. Chem.* **2022,** *65* (13), 8686-8698.
15. Douangamath, A.; Fearon, D.; Gehrtz, P.; Krojer, T.; Lukacik, P.; Owen, C. D.; Resnick, E.; Strain-

Damerell, C.; Aimon, A.; Ábrányi-Balogh, P.; Brandão-Neto, J.; Carbery, A.; Davison, G.; Dias, A.; Downes, T. D.; Dunnett, L.; Fairhead, M.; Firth, J. D.; Jones, S. P.; Keeley, A.; Keserü, G. M.; Klein, H. F.; Martin, M. P.; Noble, M. E. M.; O'Brien, P.; Powell, A.; Reddi, R. N.; Skyner, R.; Snee, M.; Waring, M. J.; Wild, C.; London, N.; von Delft, F.; Walsh, M. A., Crystallographic and electrophilic fragment screening of the SARS-CoV-2 main protease. *Nat. Commun.* **2020,** *11* (1), 5047.

16. Kim, Y.; Lovell, S.; Tiew, K.-C.; Mandadapu, S. R.; Alliston, K. R.; Battaile, K. P.; Groutas, W. C.; Chang, K.-O., Broad-spectrum antivirals against 3C or 3C-like proteases of picornaviruses, noroviruses, and coronaviruses. *J. Virol.* **2012,** *86* (21), 11754-11762.
17. Hu, Y.; Lewandowski, E. M.; Tan, H.; Zhang, X.; Morgan, R. T.; Zhang, X.; Jacobs, L. M. C.; Butler, S. G.; Gongora, M. V.; Choy, J.; Deng, X.; Chen, Y.; Wang, J., Naturally occurring mutations of SARS-CoV-2 main protease confer drug resistance to nirmatrelvir. *bioRxiv* **2022**, 2022.06.28.497978; doi: https://doi.org/10.1101/2022.06.28.497978.
18. Moghadasi, S. A.; Heilmann, E.; Khalil, A. M.; Nnabuife, C.; Kearns, F. L.; Ye, C.; Moraes, S. N.; Costacurta, F.; Esler, M. A.; Aihara, H.; von Laer, D.; Martinez-Sobrido, L.; Palzkill, T.; Amaro, R. E.; Harris, R. S., Transmissible SARS-CoV-2 variants with resistance to clinical protease inhibitors. *bioRxiv* **2022**, 2022.08.07.503099; doi: https://doi.org/10.1101/2022.08.07.503099.
19. Shin, D.; Mukherjee, R.; Grewe, D.; Bojkova, D.; Baek, K.; Bhattacharya, A.; Schulz, L.; Widera, M.; Mehdipour, A. R.; Tascher, G.; Geurink, P. P.; Wilhelm, A.; van der Heden van Noort, G. J.; Ovaa, H.; Müller, S.; Knobeloch, K.-P.; Rajalingam, K.; Schulman, B. A.; Cinatl, J.; Hummer, G.; Ciesek, S.; Dikic, I., Papain-like protease regulates SARS-CoV-2 viral spread and innate immunity. *Nature* **2020,** *587* (7835), 657-662.
20. Klemm, T.; Ebert, G.; Calleja, D. J.; Allison, C. C.; Richardson, L. W.; Bernardini, J. P.; Lu, B. G. C.; Kuchel, N. W.; Grohmann, C.; Shibata, Y.; Gan, Z. Y.; Cooney, J. P.; Doerflinger, M.; Au, A. E.; Blackmore, T. R.; van der Heden van Noort, G. J.; Geurink, P. P.; Ovaa, H.; Newman, J.; Riboldi-Tunnicliffe, A.; Czabotar, P. E.; Mitchell, J. P.; Feltham, R.; Lechtenberg, B. C.; Lowes, K. N.; Dewson, G.; Pellegrini, M.; Lessene, G.; Komander, D., Mechanism and inhibition of the papain-like protease, PLpro, of SARS-CoV-2. *EMBO J.* **2020,** *39* (18), e106275.

7. ^{1}H and ^{13}C NMR spectra of novel alkyne-containing SARS-CoV-2 M^{pro} inhibitors prepared for this study

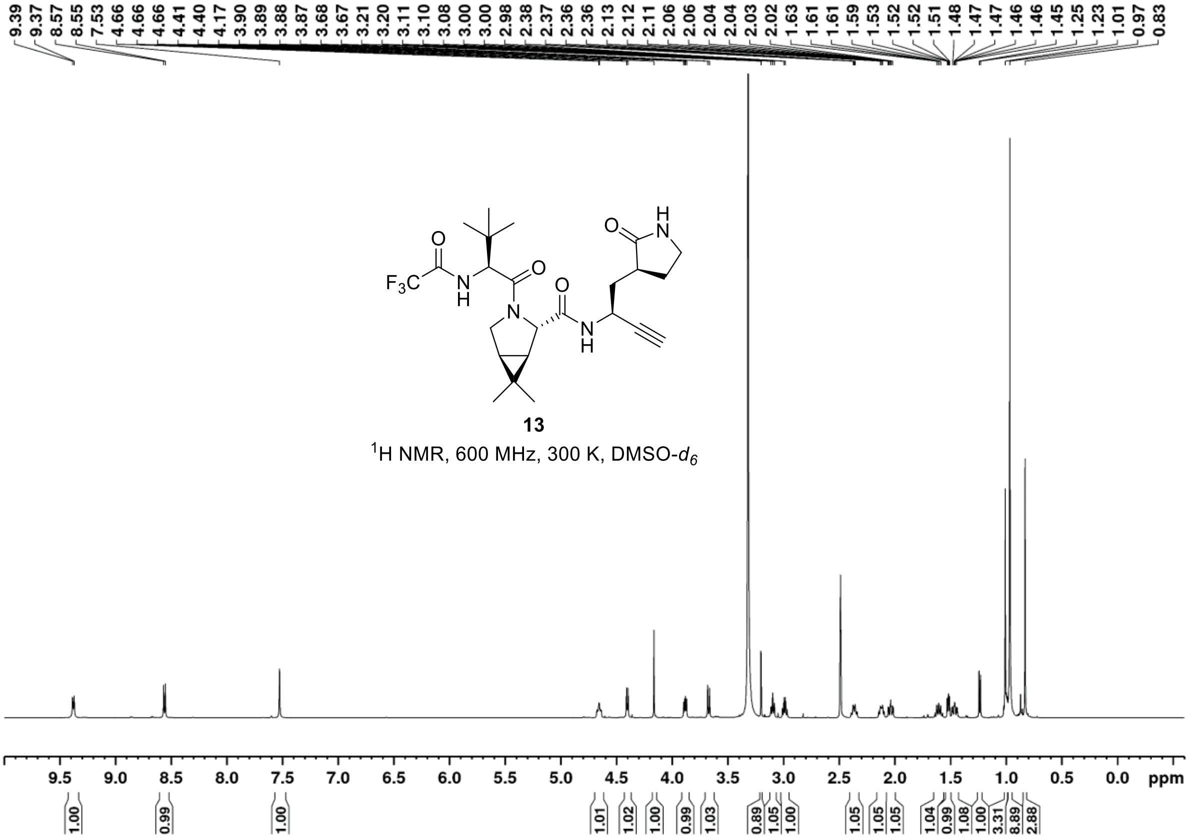

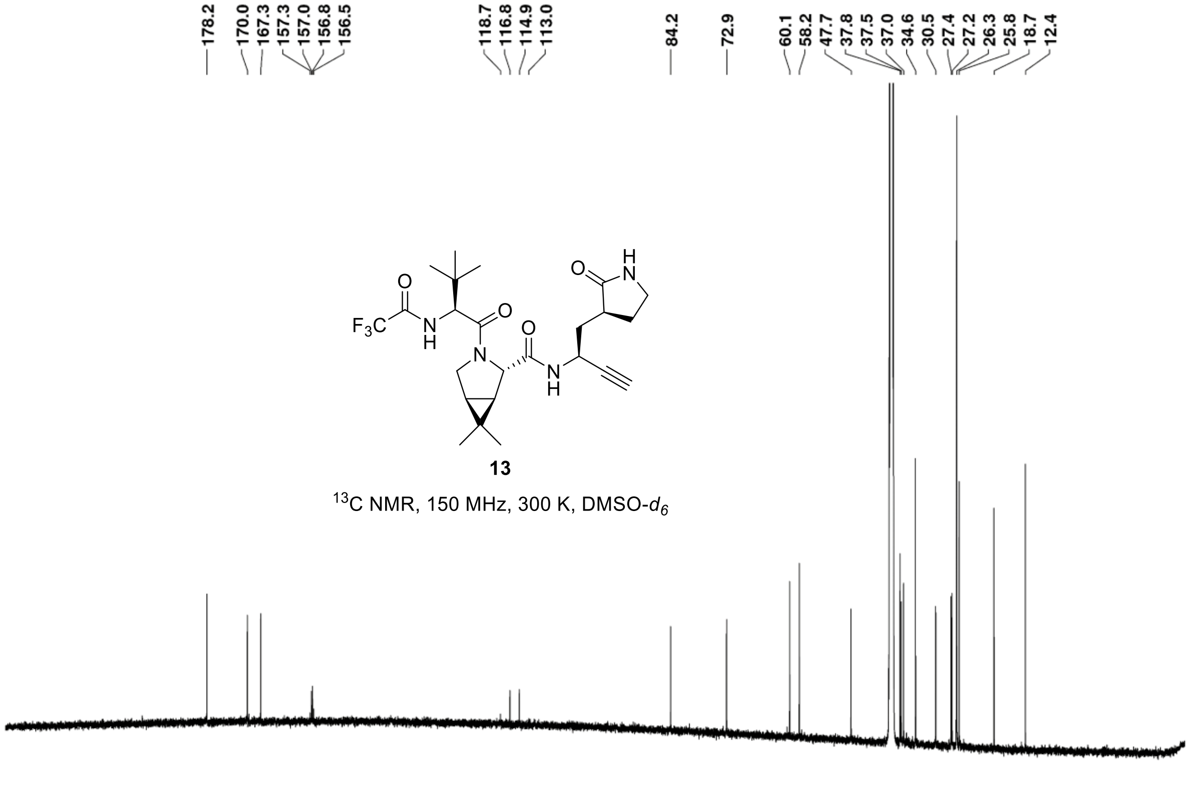

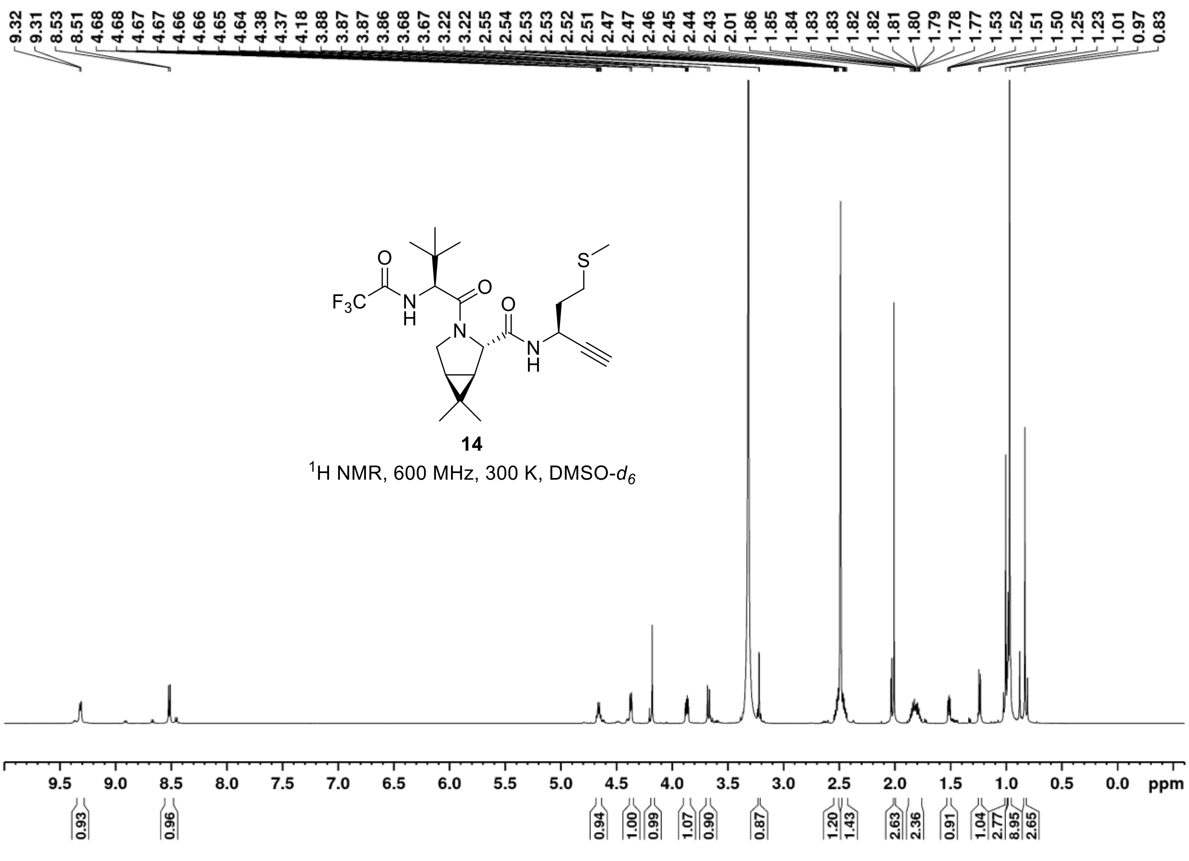
9.32 9.31 8.53 8.51 4.68 4.68 4.67 4.67 4.66 4.66 4.65 4.64 4.38 4.37 4.18 3.88 3.87 3.87 3.86 3.68 3.67 3.67 3.22 3.22 2.55 2.54 2.53 2.53 2.52 2.51 2.47 2.47 2.46 2.45 2.44 2.43 2.01 1.86 1.85 1.84 1.83 1.83 1.82 1.82 1.81 1.80 1.79 1.78 1.77 1.53 1.52 1.51 1.50 1.25 1.23 1.01 0.97 0.83
F3C
O
N
H
O
N
O
N
H
S
14
1H NMR, 600 MHz, 300 K, DMSO-d6
9.5 9.0 8.5 8.0 7.5 7.0 6.5 6.0 5.5 5.0 4.5 4.0 3.5 3.0 2.5 2.0 1.5 1.0 0.5 0.0 ppm
0.93 0.96 0.94 1.00 0.99 1.07 0.90 0.87 1.20 1.43 2.63 2.36 0.91 1.04 2.77 8.95 2.65

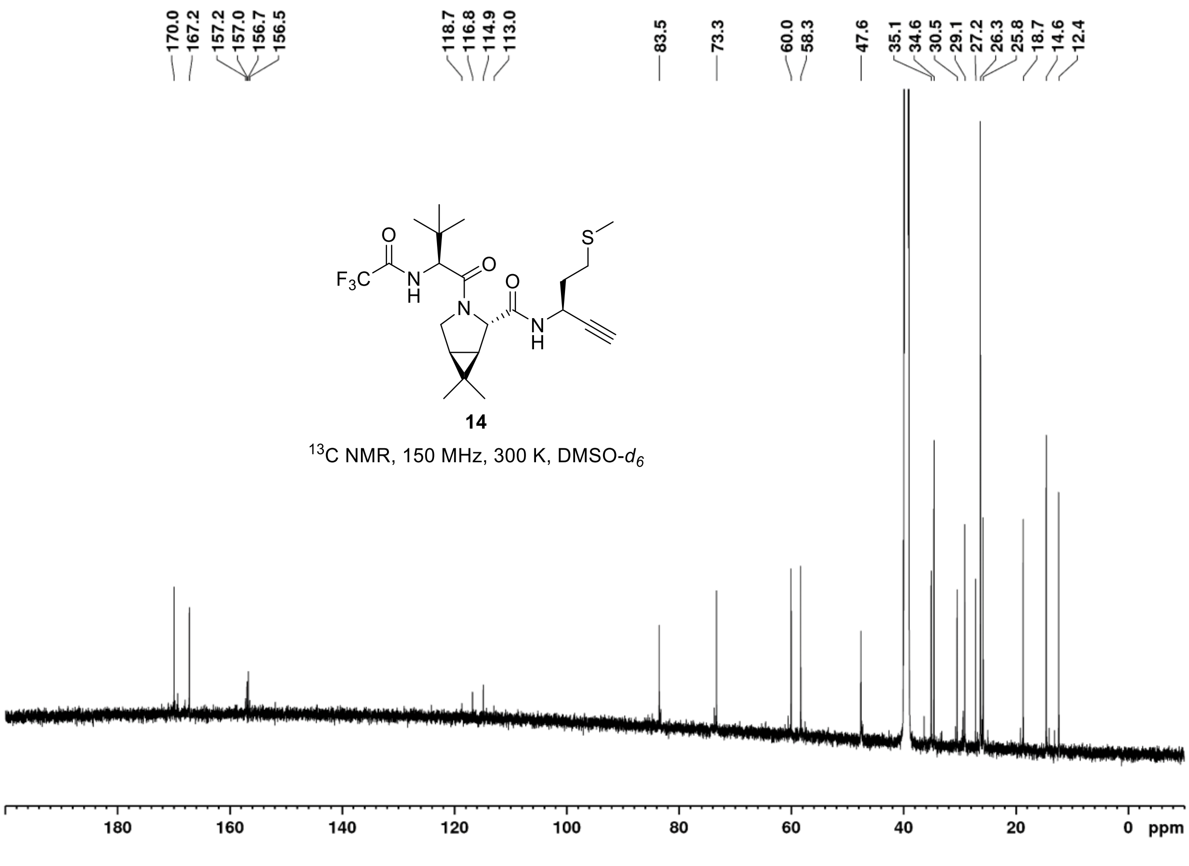
170.0 167.2 157.2 157.0 156.7 156.5 118.7 116.8 114.9 113.0 83.5 73.3 60.0 58.3 47.6 35.1 34.6 30.5 29.1 27.2 26.3 25.8 18.7 14.6 12.4
F3C
O
N
H
O
N
O
N
H
S
14
13C NMR, 150 MHz, 300 K, DMSO-d6
180 160 140 120 100 80 60 40 20 0 ppm

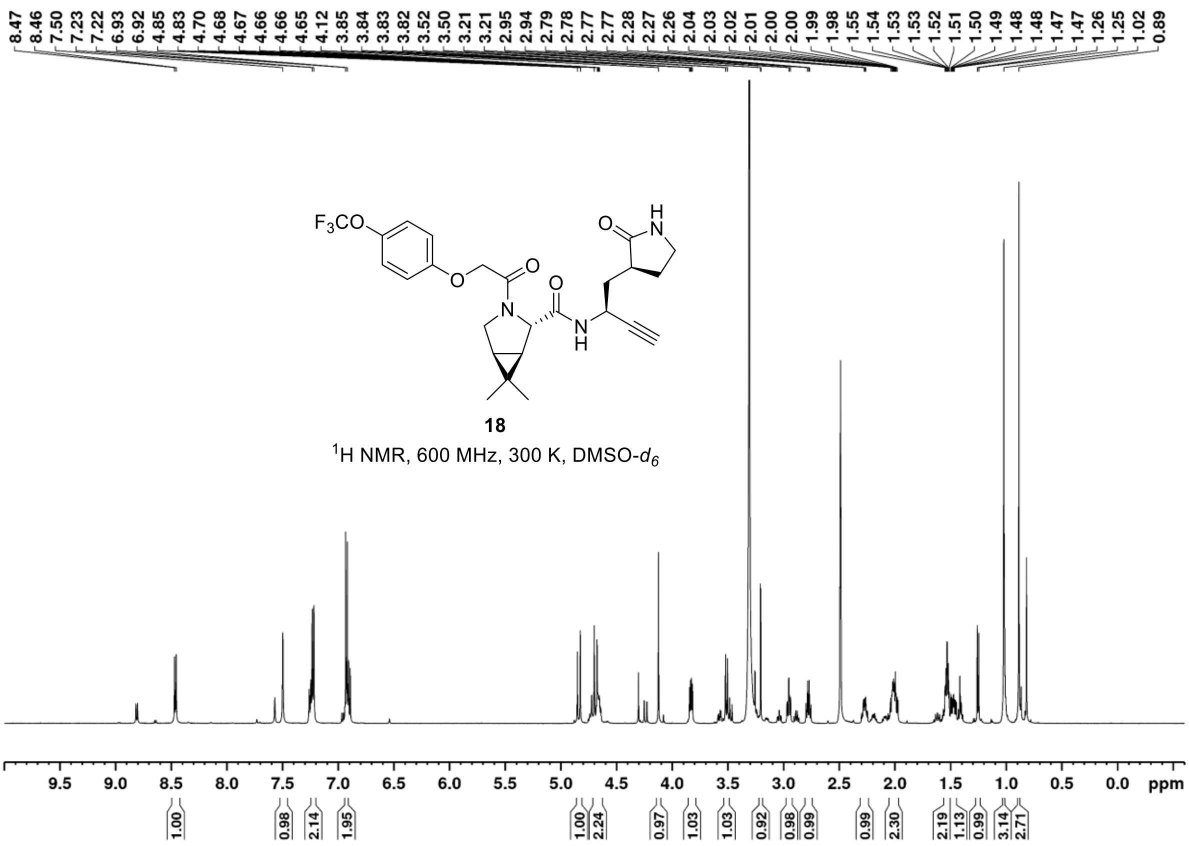

18
^{1}H NMR, 600 MHz, 300 K, DMSO-d_6

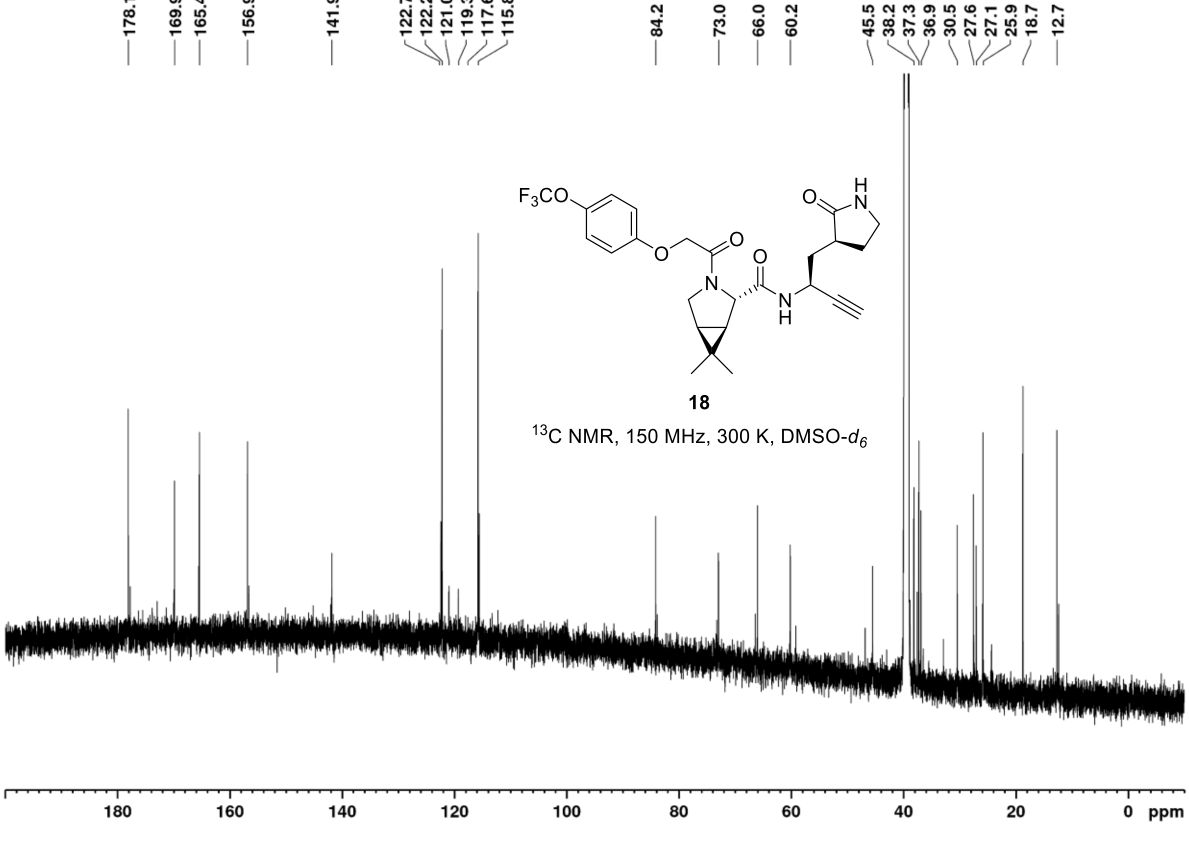

18
^{13}C NMR, 150 MHz, 300 K, DMSO-d_6

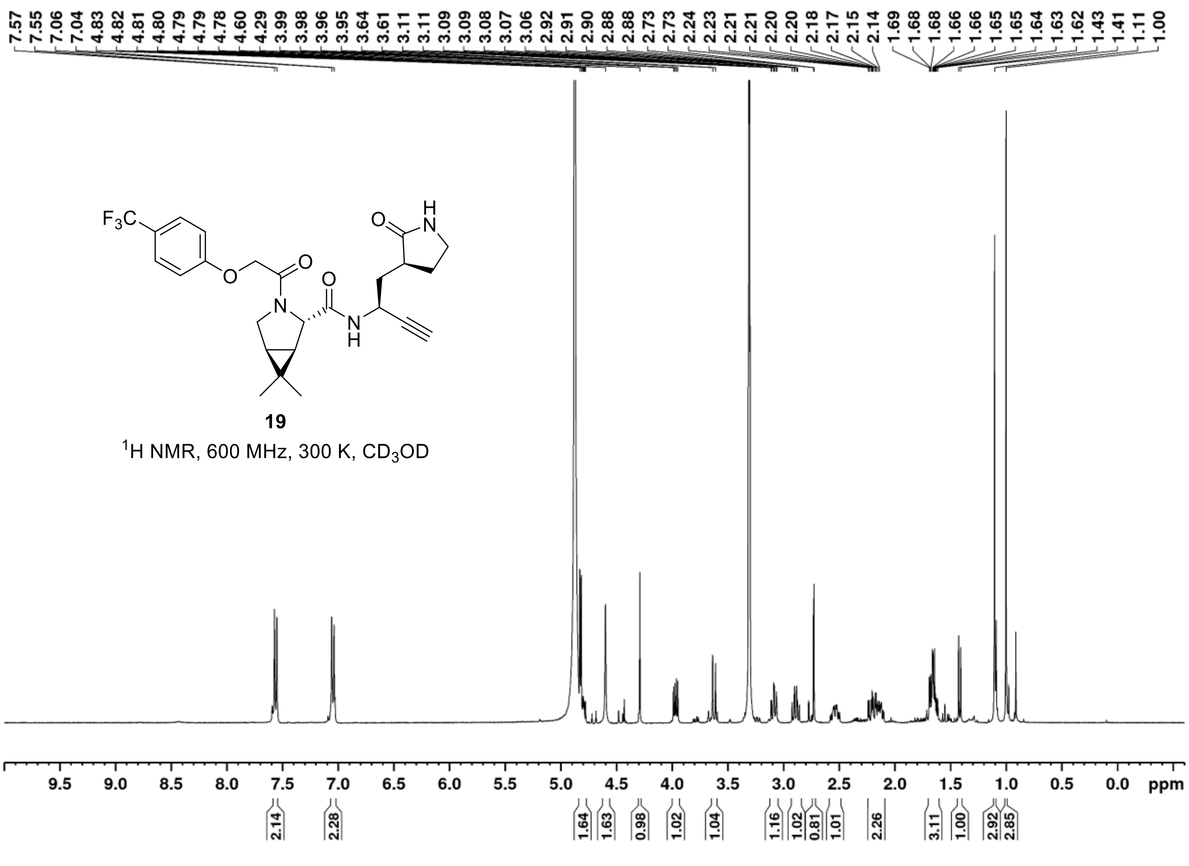
7.57 7.55 7.06 7.04 4.83 4.82 4.81 4.80 4.79 4.79 4.78 4.60 4.29 3.99 3.98 3.96 3.95 3.64 3.61 3.11 3.11 3.09 3.09 3.08 3.07 3.06 2.92 2.91 2.90 2.88 2.88 2.73 2.73 2.24 2.23 2.21 2.21 2.20 2.20 2.18 2.17 2.15 2.14 1.69 1.68 1.68 1.66 1.66 1.65 1.65 1.64 1.63 1.62 1.43 1.41 1.11 1.00
19
^{1}H NMR, 600 MHz, 300 K, CD_3OD
ppm
2.14 2.28 1.64 1.63 0.98 1.02 1.04 1.16 1.02 0.81 1.01 2.26 3.11 1.00 2.92 2.85

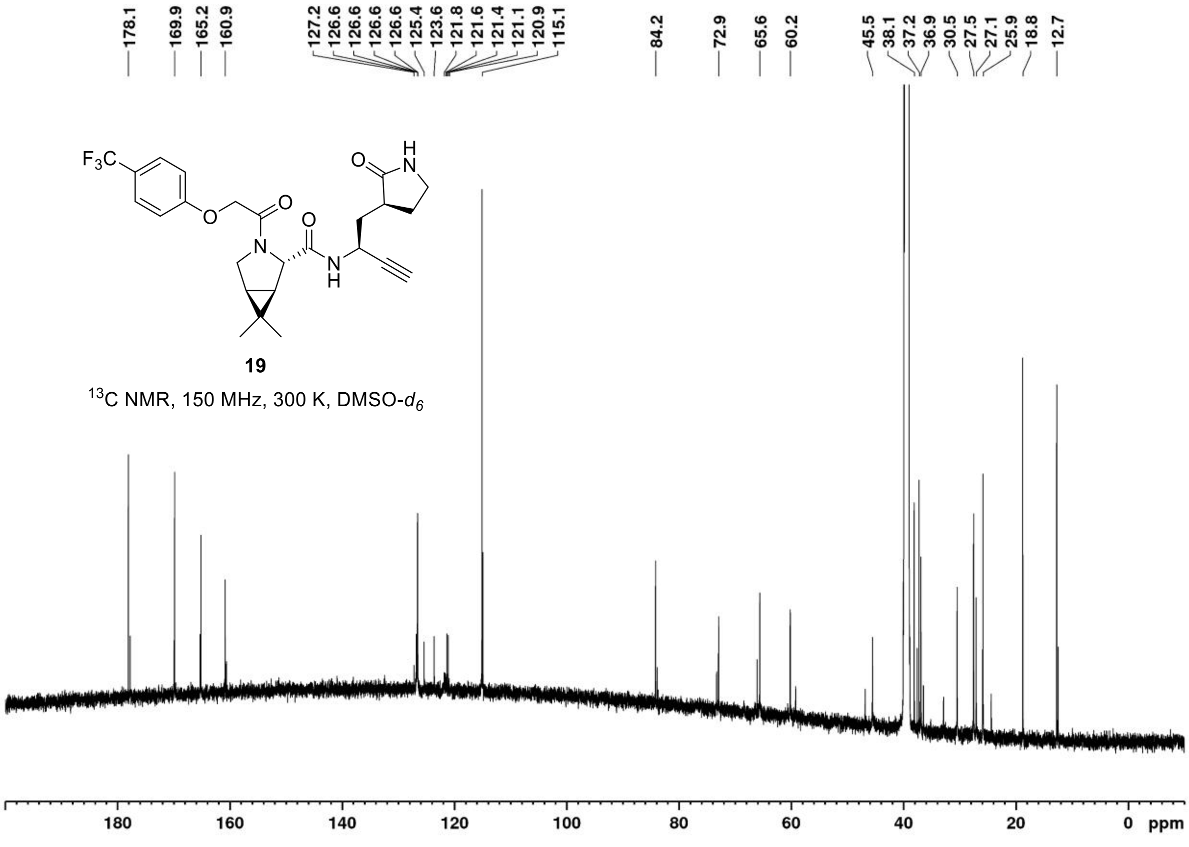
178.1 169.9 165.2 160.9 127.2 126.6 126.6 126.6 125.4 123.6 121.8 121.6 121.4 121.1 120.9 115.1 84.2 72.9 65.6 60.2 45.5 38.1 37.2 36.9 30.5 27.5 27.1 25.9 18.8 12.7
19
^{13}C NMR, 150 MHz, 300 K, DMSO-d_6
ppm

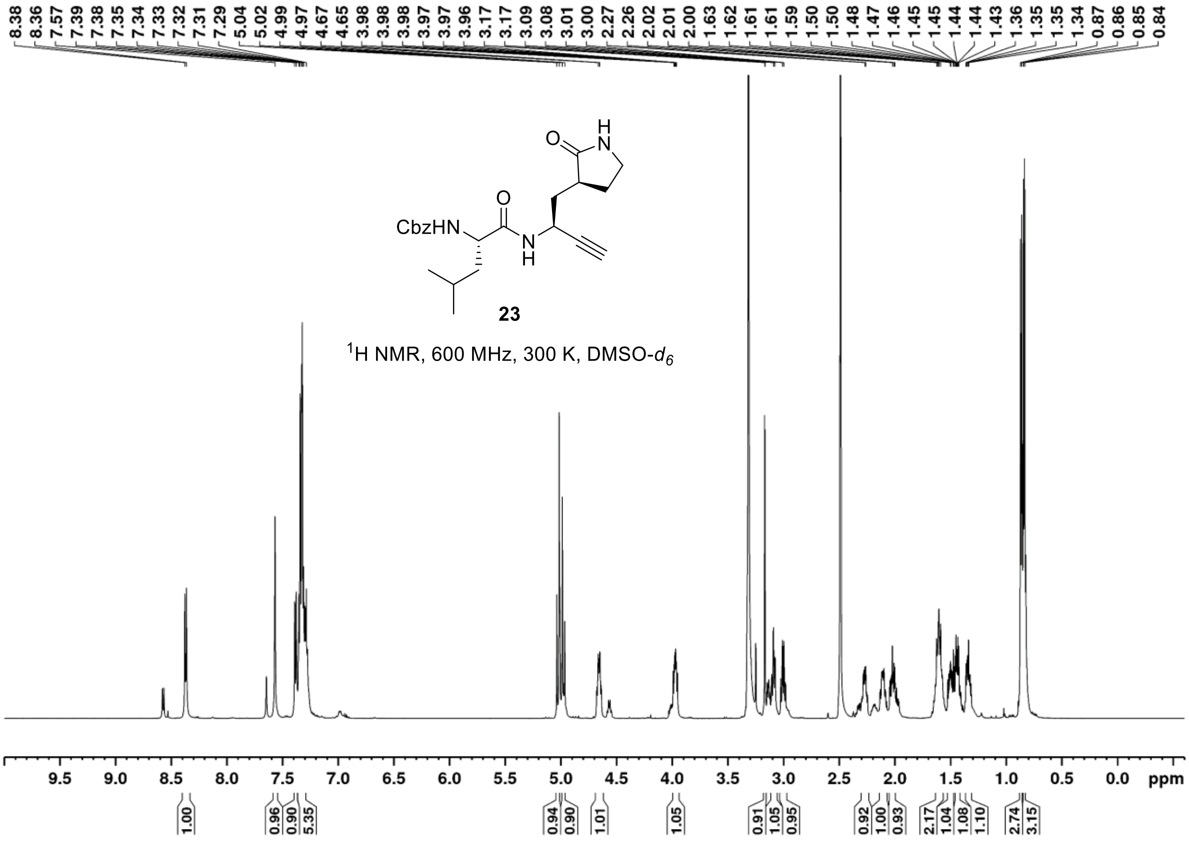
8.38 8.36 7.57 7.39 7.38 7.35 7.34 7.33 7.32 7.31 7.29 5.04 5.02 4.99 4.97 4.67 4.65 3.98 3.98 3.98 3.97 3.97 3.96 3.17 3.09 3.08 3.01 3.00 2.27 2.26 2.02 2.01 2.00 1.63 1.62 1.61 1.61 1.59 1.50 1.50 1.48 1.47 1.46 1.45 1.45 1.44 1.44 1.43 1.36 1.35 1.35 1.34 0.87 0.86 0.85 0.84
O
H
N
CbzHN
O
N
H
23
^{1}H NMR, 600 MHz, 300 K, DMSO-d_6
9.5 9.0 8.5 8.0 7.5 7.0 6.5 6.0 5.5 5.0 4.5 4.0 3.5 3.0 2.5 2.0 1.5 1.0 0.5 0.0 ppm
1.00 0.96 0.90 5.35 0.94 0.90 1.01 1.05 0.91 1.05 0.95 0.92 1.00 0.93 2.17 1.04 1.08 1.10 2.74 3.15

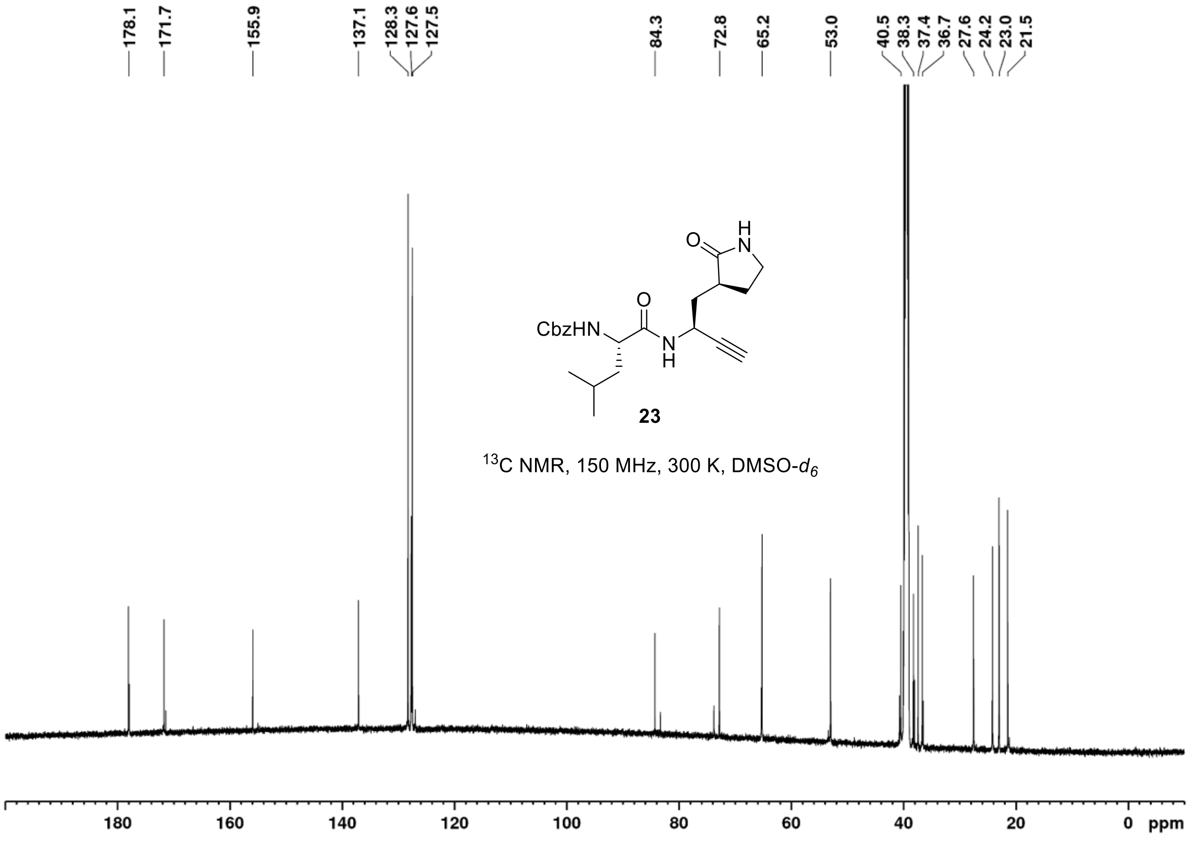
178.1 171.7 155.9 137.1 128.3 127.6 127.5 84.3 72.8 65.2 53.0 40.5 38.3 37.4 36.7 27.6 24.2 23.0 21.5
O
H
N
CbzHN
O
N
H
23
^{13}C NMR, 150 MHz, 300 K, DMSO-d_6
180 160 140 120 100 80 60 40 20 0 ppm

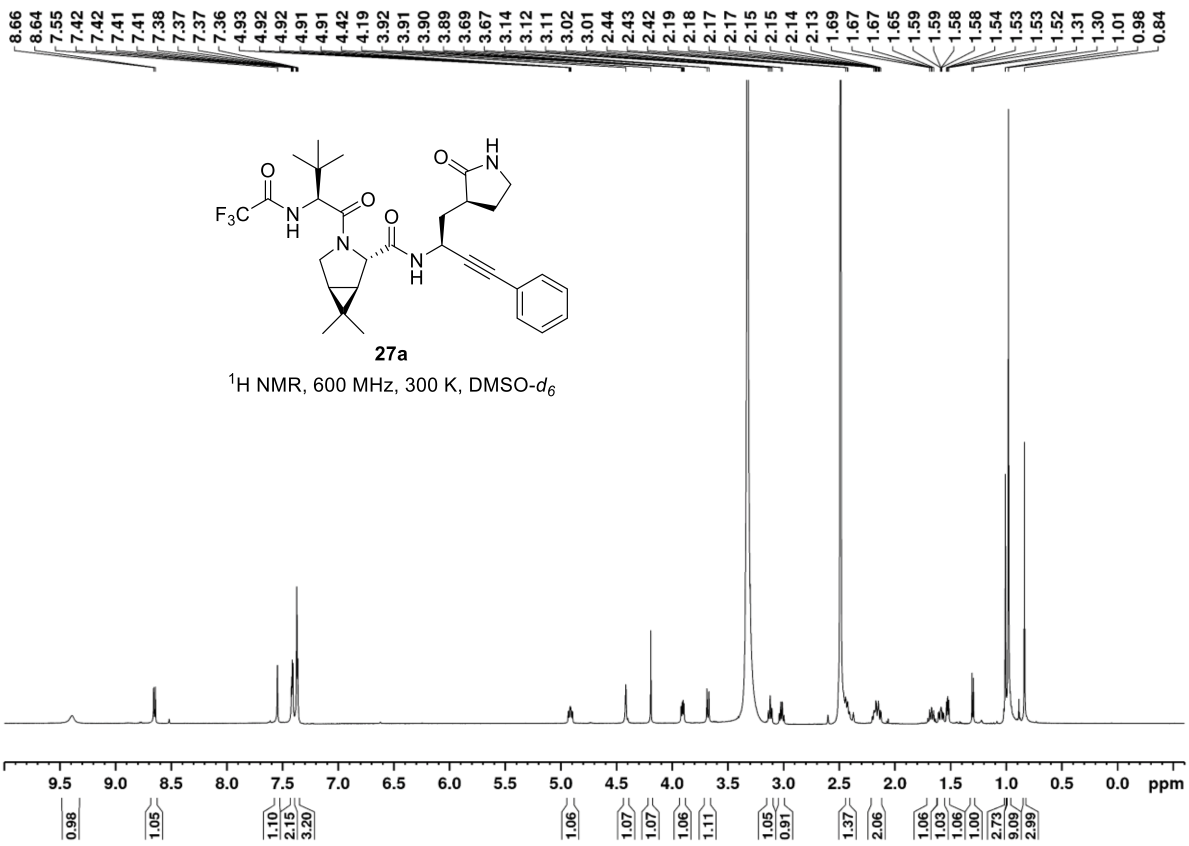

8.66 8.64 7.55 7.42 7.42 7.41 7.41 7.38 7.37 7.37 7.36 4.93 4.92 4.92 4.91 4.91 4.42 4.19 3.92 3.91 3.90 3.89 3.69 3.67 3.14 3.12 3.11 3.02 3.01 2.44 2.43 2.42 2.19 2.18 2.17 2.17 2.15 2.15 2.14 2.13 1.69 1.67 1.67 1.65 1.59 1.59 1.58 1.58 1.54 1.53 1.53 1.52 1.31 1.30 1.01 0.98 0.84
27a
^{1}H NMR, 600 MHz, 300 K, DMSO-d_6
ppm

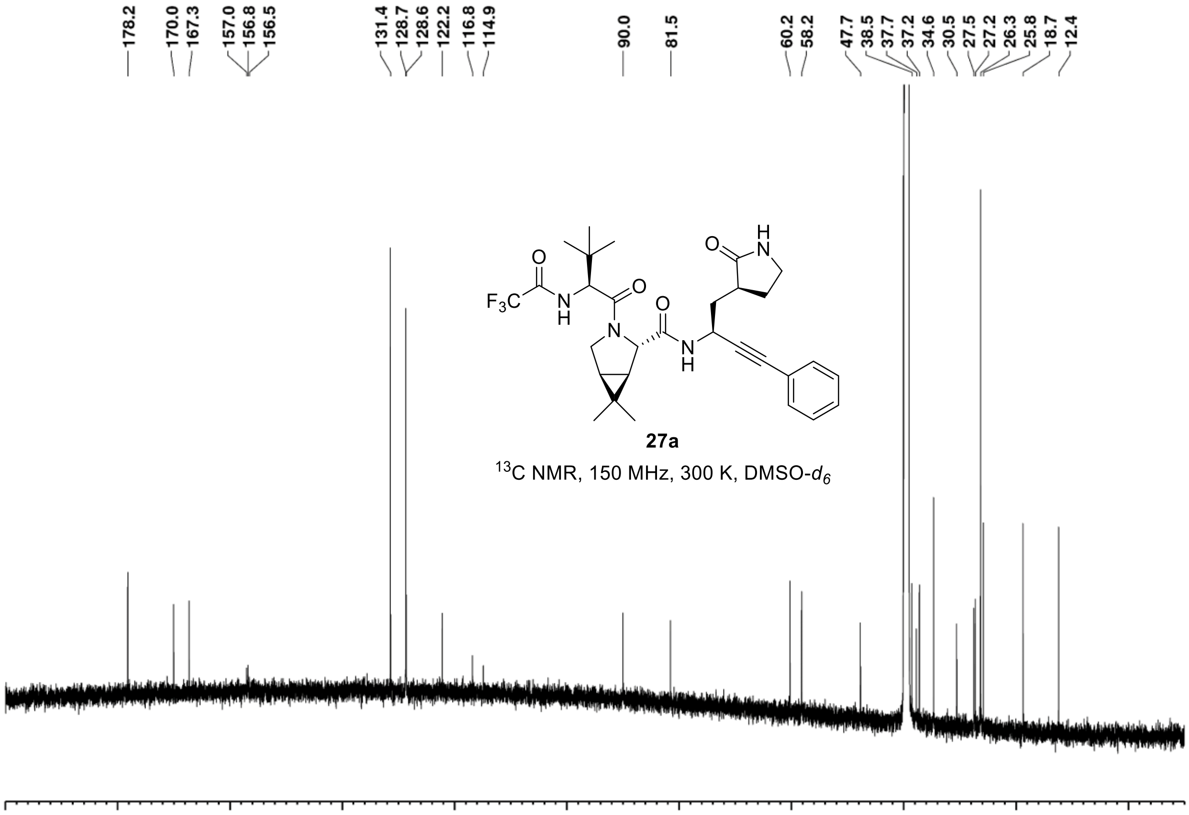

178.2 170.0 167.3 157.0 156.8 156.5 131.4 128.7 128.6 122.2 116.8 114.9 90.0 81.5 60.2 58.2 47.7 38.5 37.7 37.2 34.6 30.5 27.5 27.2 26.3 25.8 18.7 12.4
27a
^{13}C NMR, 150 MHz, 300 K, DMSO-d_6
ppm

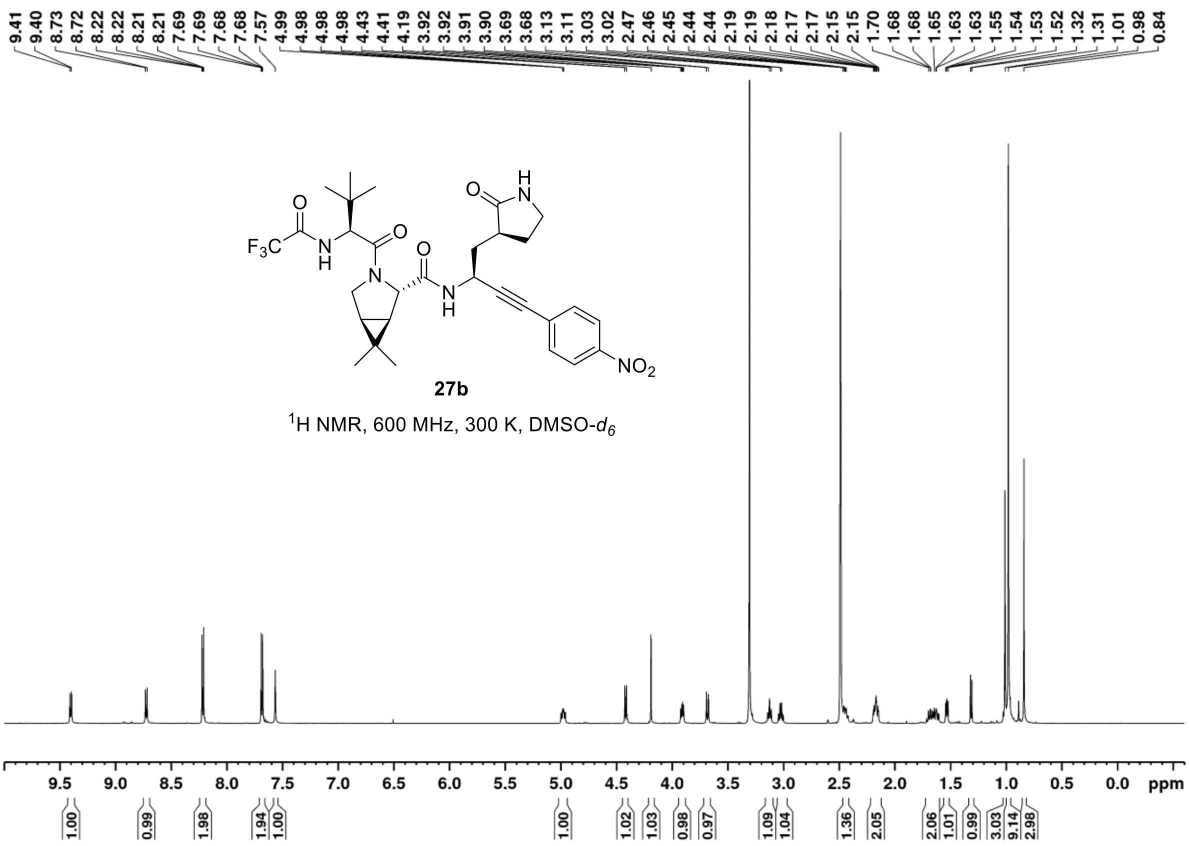

27b

^{1}H NMR, 600 MHz, 300 K, DMSO-d_6

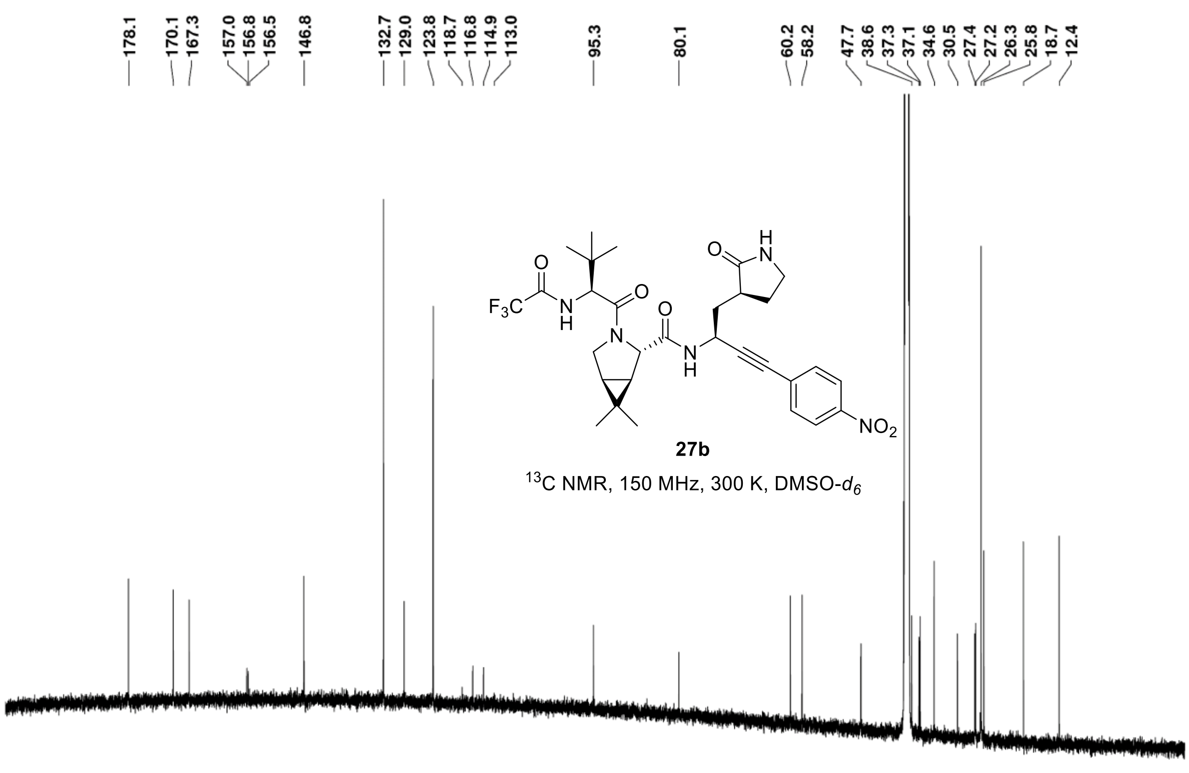

27b

^{13}C NMR, 150 MHz, 300 K, DMSO-d_6

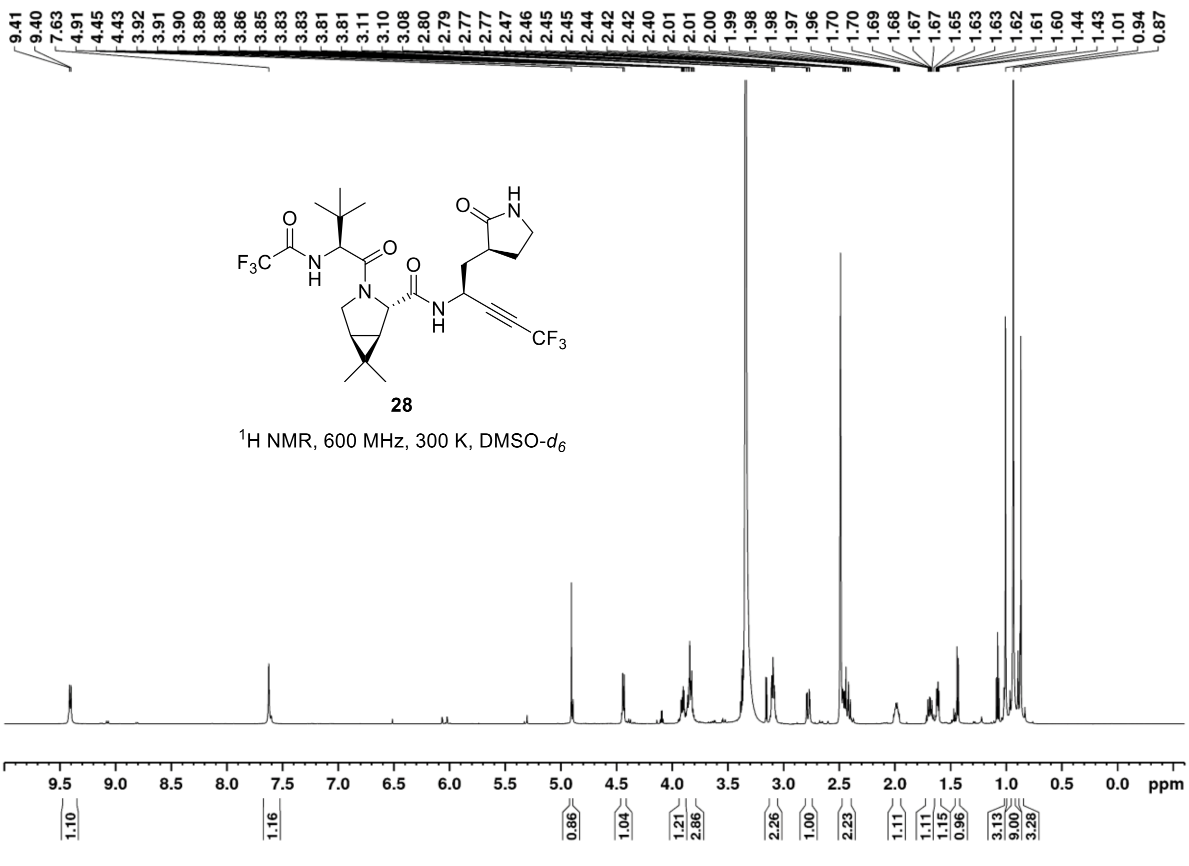
9.41 9.40 7.63 4.91 4.45 4.43 3.92 3.91 3.90 3.89 3.88 3.86 3.85 3.83 3.83 3.81 3.81 3.11 3.10 3.08 2.80 2.79 2.77 2.77 2.47 2.46 2.45 2.45 2.44 2.42 2.42 2.40 2.01 2.01 2.00 1.99 1.98 1.98 1.97 1.96 1.70 1.70 1.69 1.68 1.67 1.67 1.65 1.63 1.63 1.62 1.61 1.60 1.44 1.43 1.01 0.94 0.87
O
N
H
F3C
H
N
O
N
O
N
H
CF3
28
1H NMR, 600 MHz, 300 K, DMSO-d6
9.5 9.0 8.5 8.0 7.5 7.0 6.5 6.0 5.5 5.0 4.5 4.0 3.5 3.0 2.5 2.0 1.5 1.0 0.5 0.0 ppm
1.10 1.16 0.86 1.04 1.21 2.86 2.26 1.00 2.23 1.11 1.11 1.15 0.96 3.13 9.00 3.28

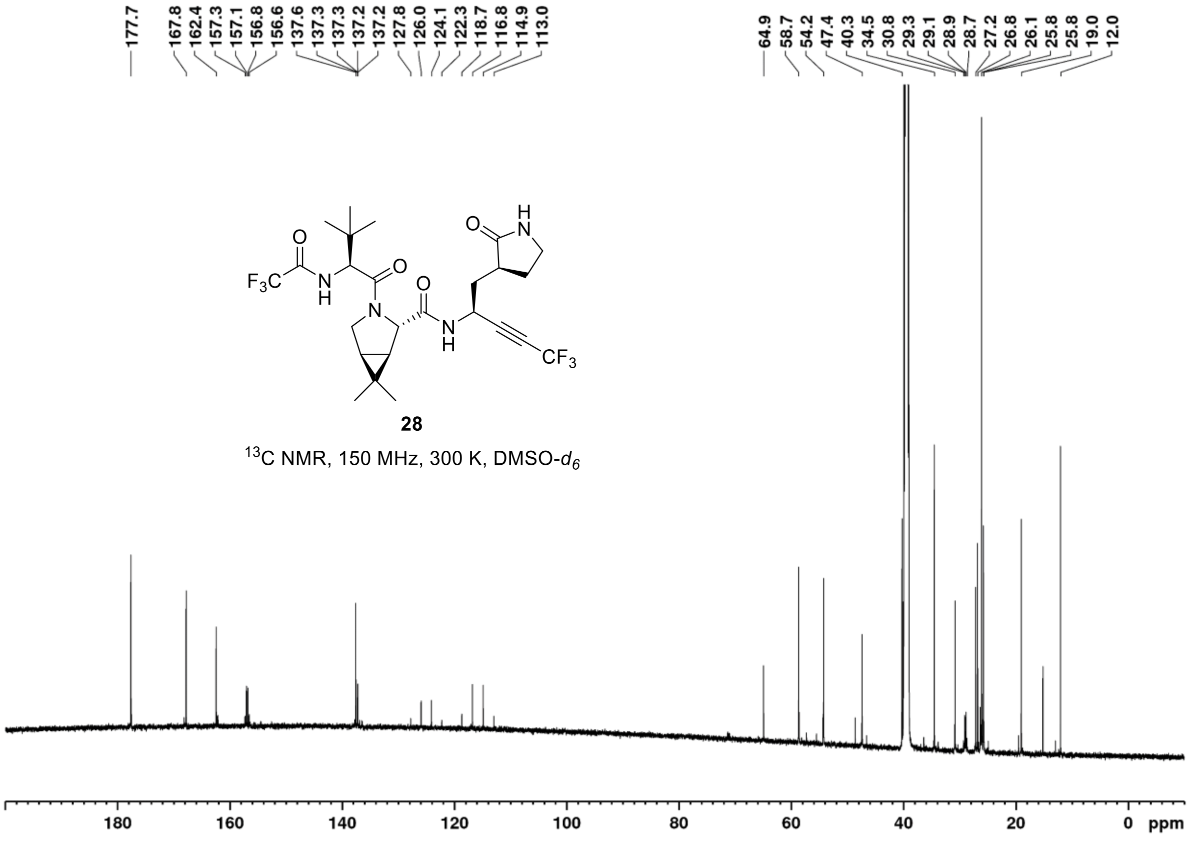
177.7 167.8 162.4 157.3 157.1 156.8 156.6 137.6 137.3 137.3 137.2 137.2 127.8 126.0 124.1 122.3 118.7 116.8 114.9 113.0
64.9 58.7 54.2 47.4 40.3 34.5 30.8 29.3 29.1 28.9 28.7 27.2 26.8 26.1 25.8 19.0 12.0
O
N
H
F3C
H
N
O
N
O
N
H
CF3
28
13C NMR, 150 MHz, 300 K, DMSO-d6
180 160 140 120 100 80 60 40 20 0 ppm

8. HPLC traces of selected SARS-CoV-2 M^{pro} inhibitors prepared for this study. HPLC traces were recorded using a semi-preparative HPLC machine (Shimadzu UK Ltd.) equipped with a reverse phase column (ACE 5 C18, dimensions: 100 mm length, 21.2 mm inner diameter, 5.0 µm particle size). A linear gradient (2−98%$_{v/v}$ over 15 min) of acetonitrile in water (each containing 0.1%$_{v/v}$ trifluoroacetic acid) was used as eluent (flow rate: 12 mL/min; wavelength monitored: 220 nm). The area% of the major peak (labelled with the retention time, t_R) is ≥95% with respect to the sum of the area% of all peaks detected (excluding the injection peaks at ~1 to ~4 min; note that alkynes **13** and **28** were injected as DMSO solutions).

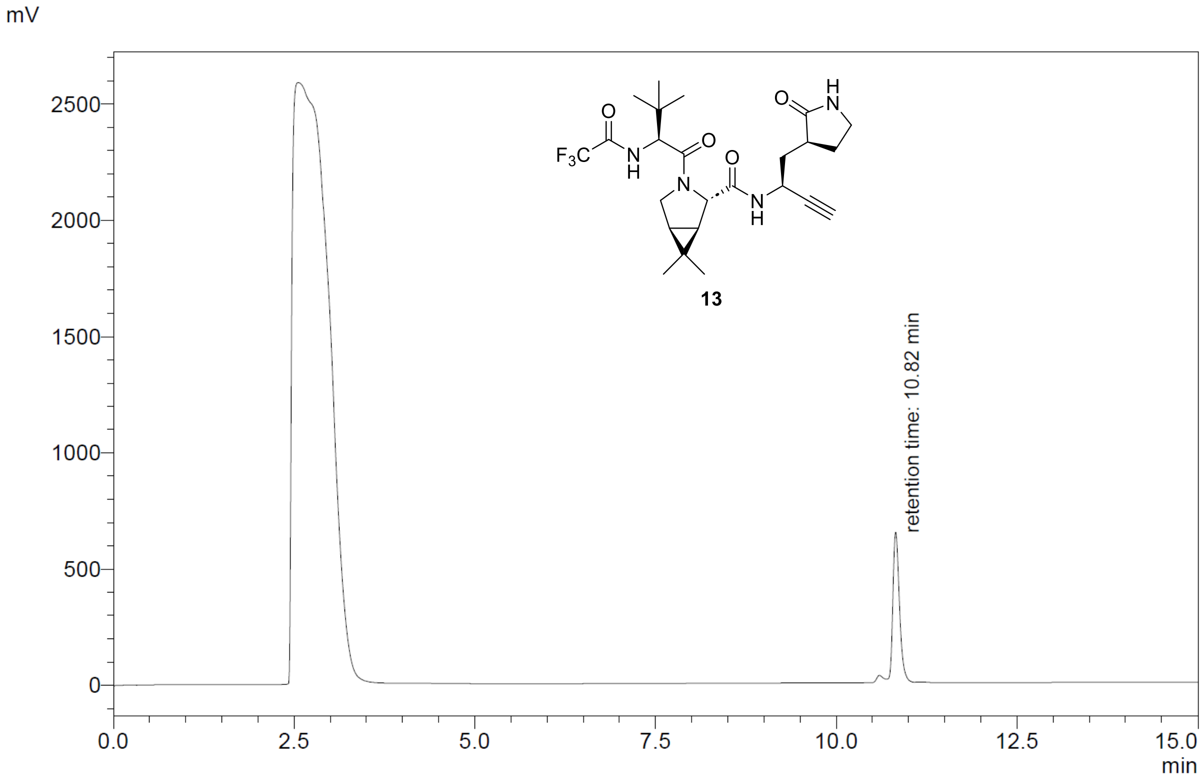

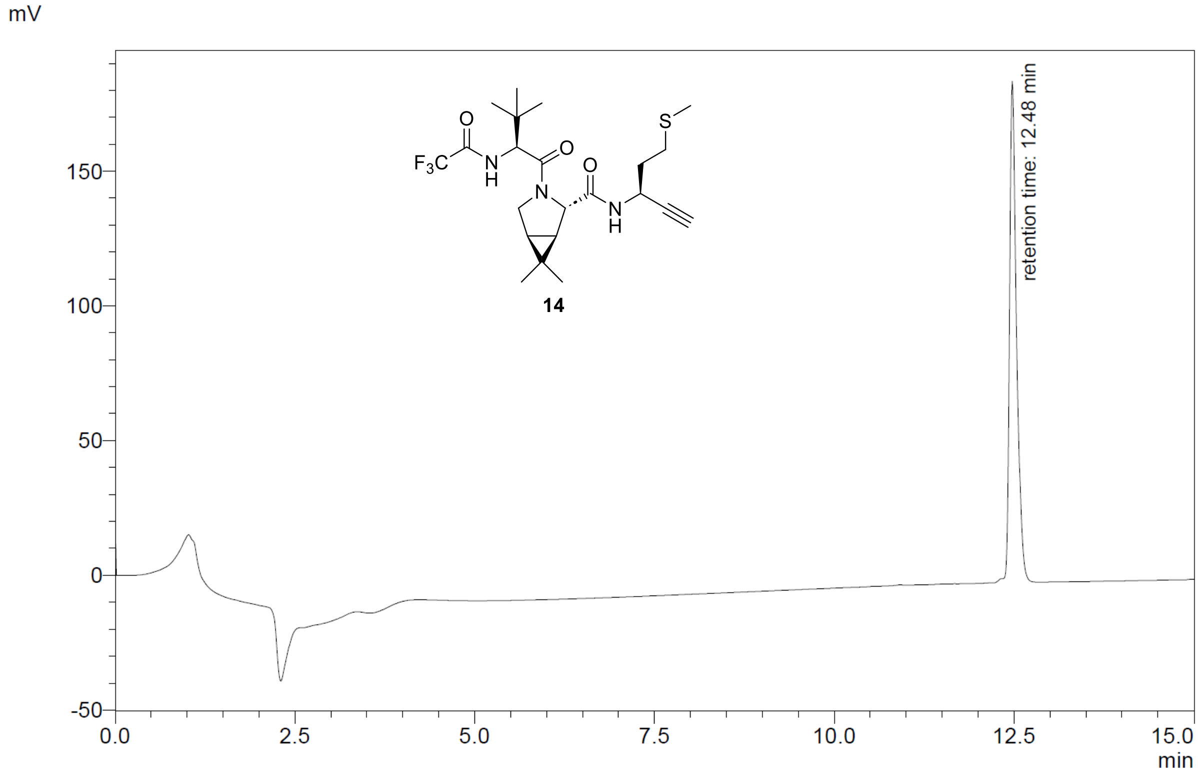
mV
150
100
50
0
-50
0.0
2.5
5.0
7.5
10.0
12.5
15.0
min
retention time: 12.48 min
F_3C
O
N
H
N
O
O
N
H
S
14

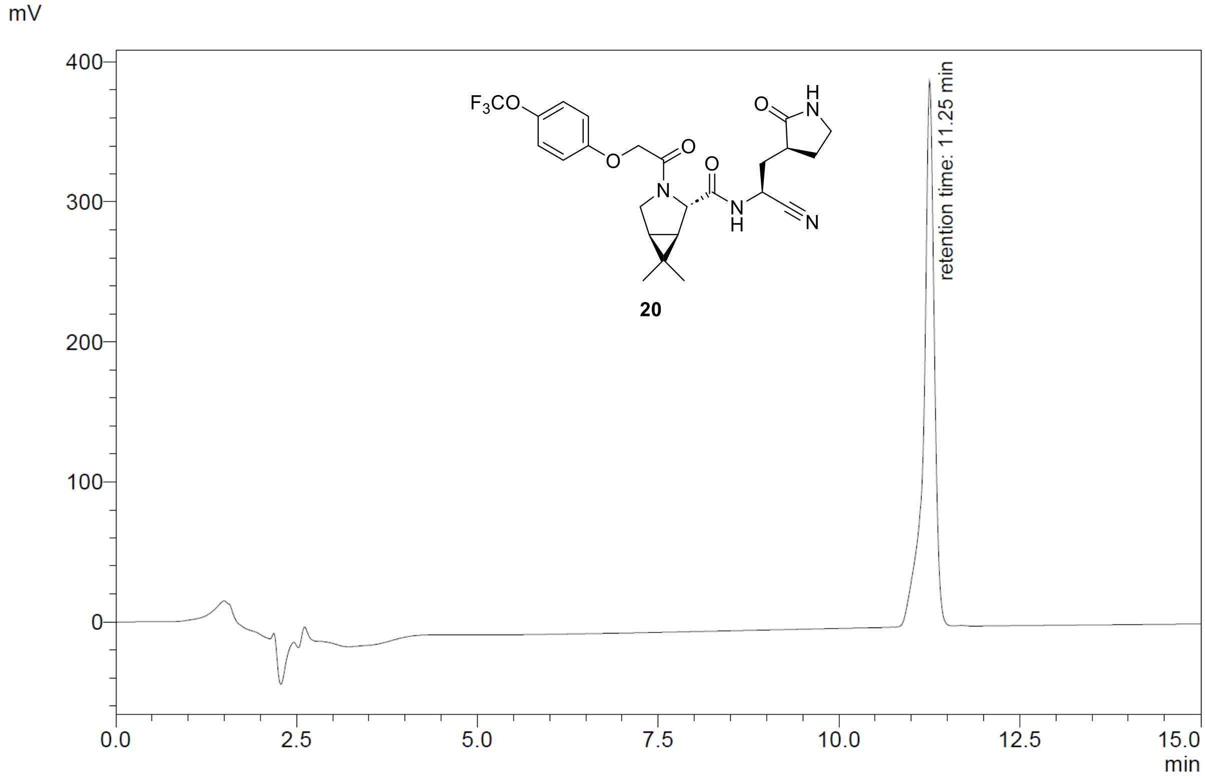
mV
400
300
200
100
0
0.0
2.5
5.0
7.5
10.0
12.5
15.0
min
retention time: 11.25 min
F_3CO
O
O
N
O
N
H
N
H
O
20

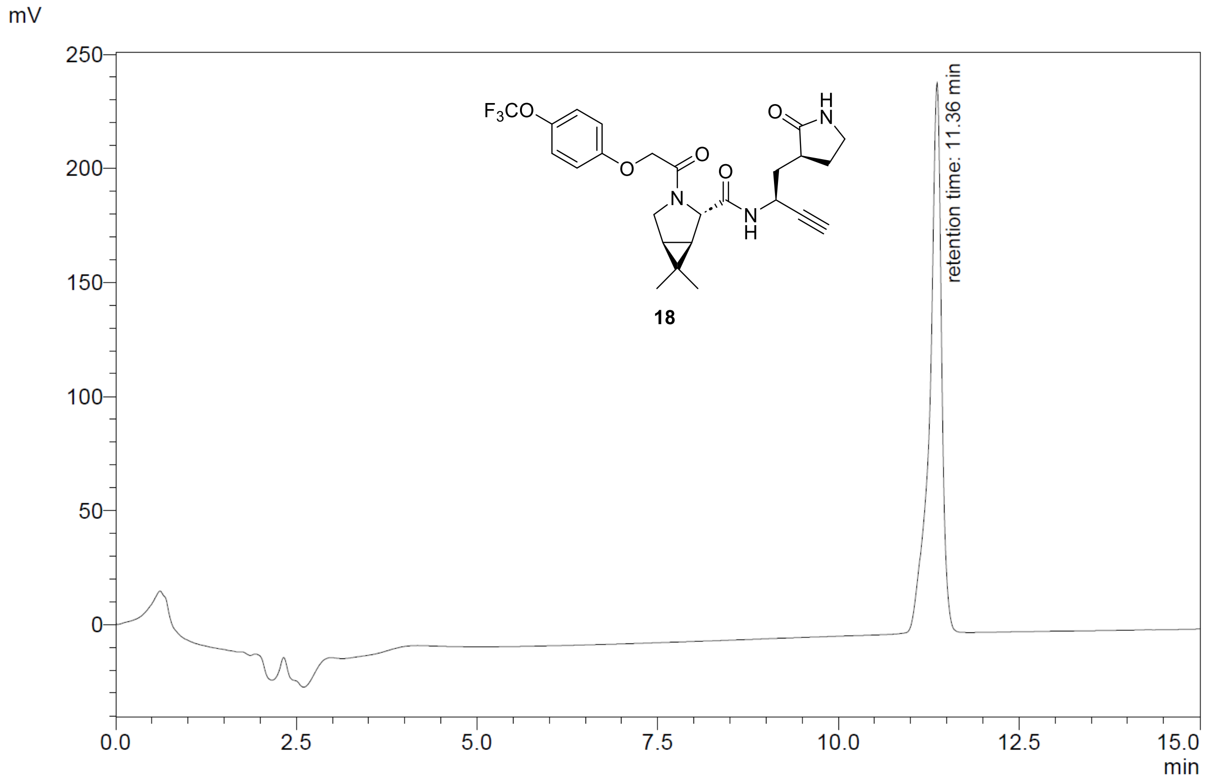
mV
250
200
150
100
50
0
0.0
2.5
5.0
7.5
10.0
12.5
15.0
min
retention time: 11.36 min
F3CO
O
O
N
O
N
H
O
H
N
18

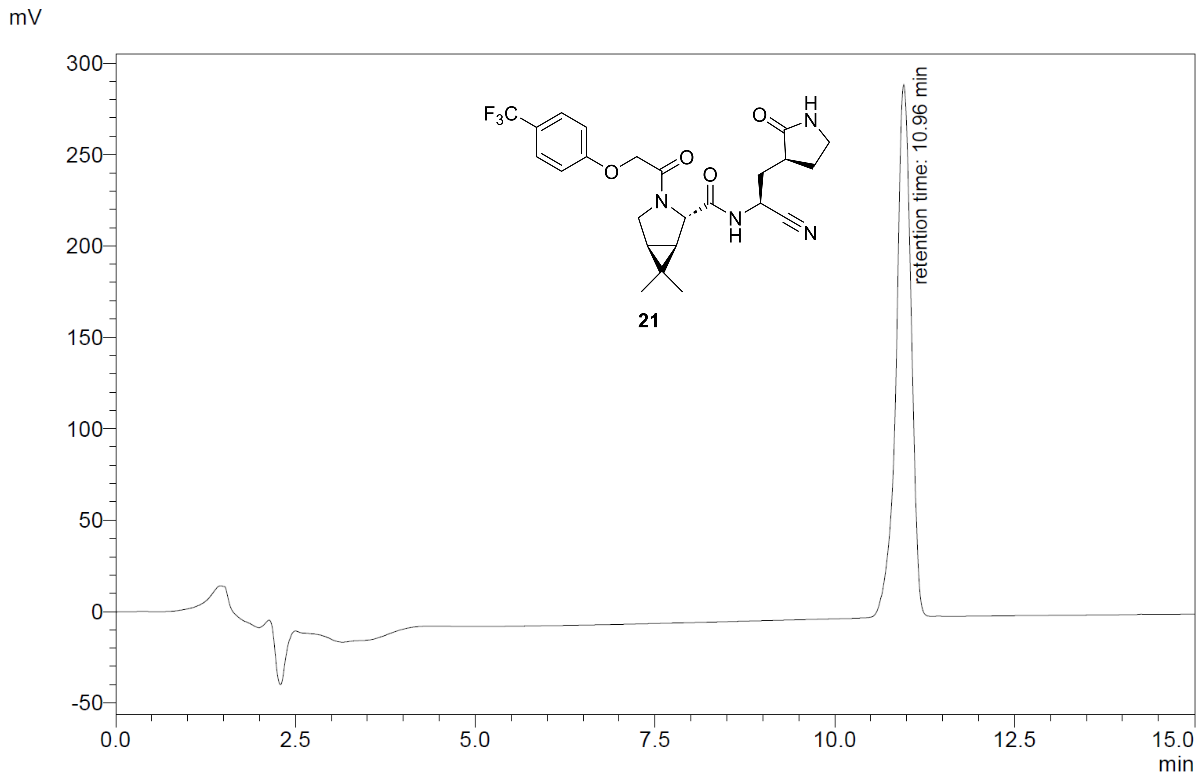
mV
300
250
200
150
100
50
0
-50
0.0
2.5
5.0
7.5
10.0
12.5
15.0
min
retention time: 10.96 min
F3C
O
O
N
O
N
H
N
O
H
N
21

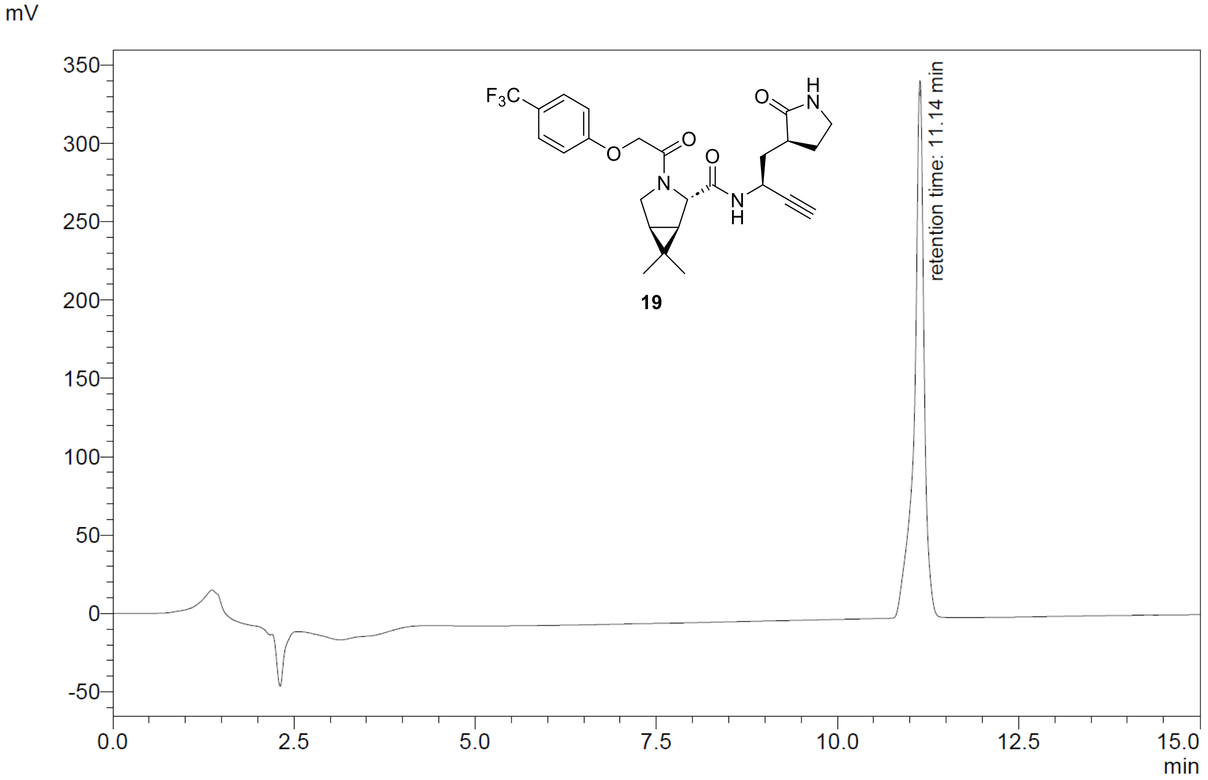
mV
350
300
250
200
150
100
50
0
-50
0.0
2.5
5.0
7.5
10.0
12.5
15.0
min
retention time: 11.14 min
19

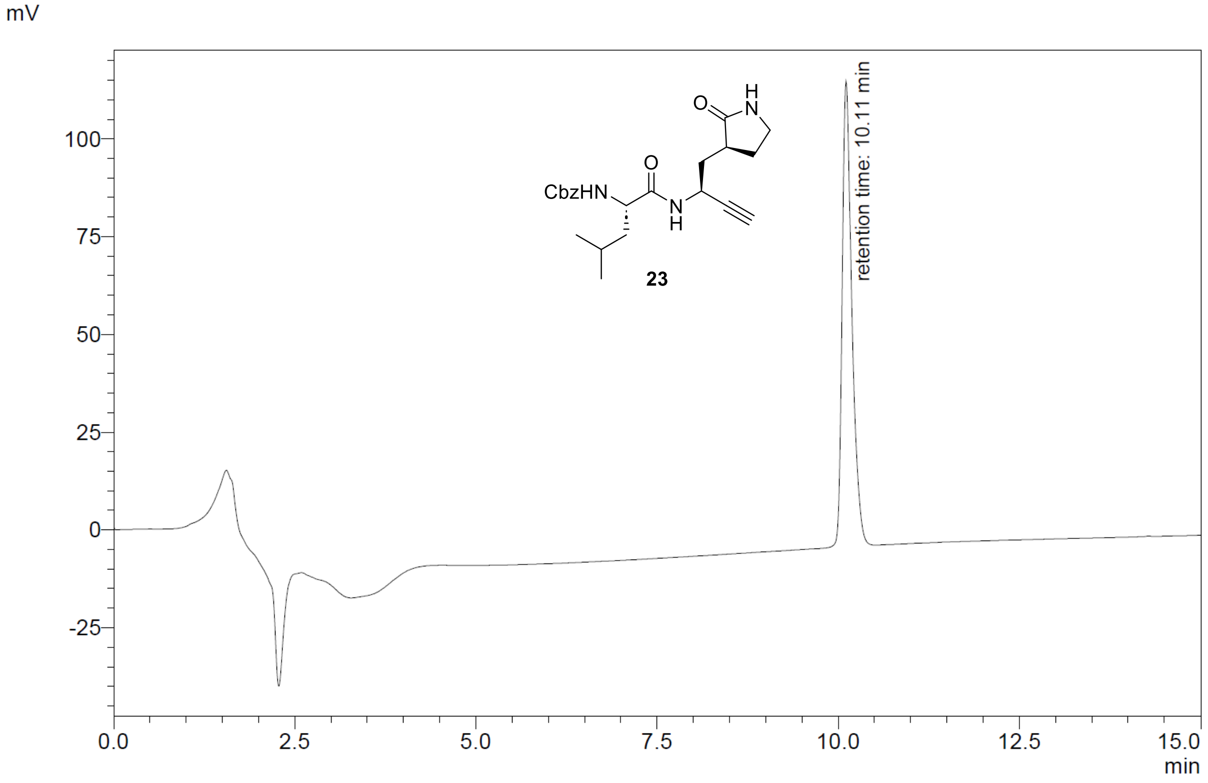
mV
100
75
50
25
0
-25
0.0
2.5
5.0
7.5
10.0
12.5
15.0
min
retention time: 10.11 min
CbzHN
23

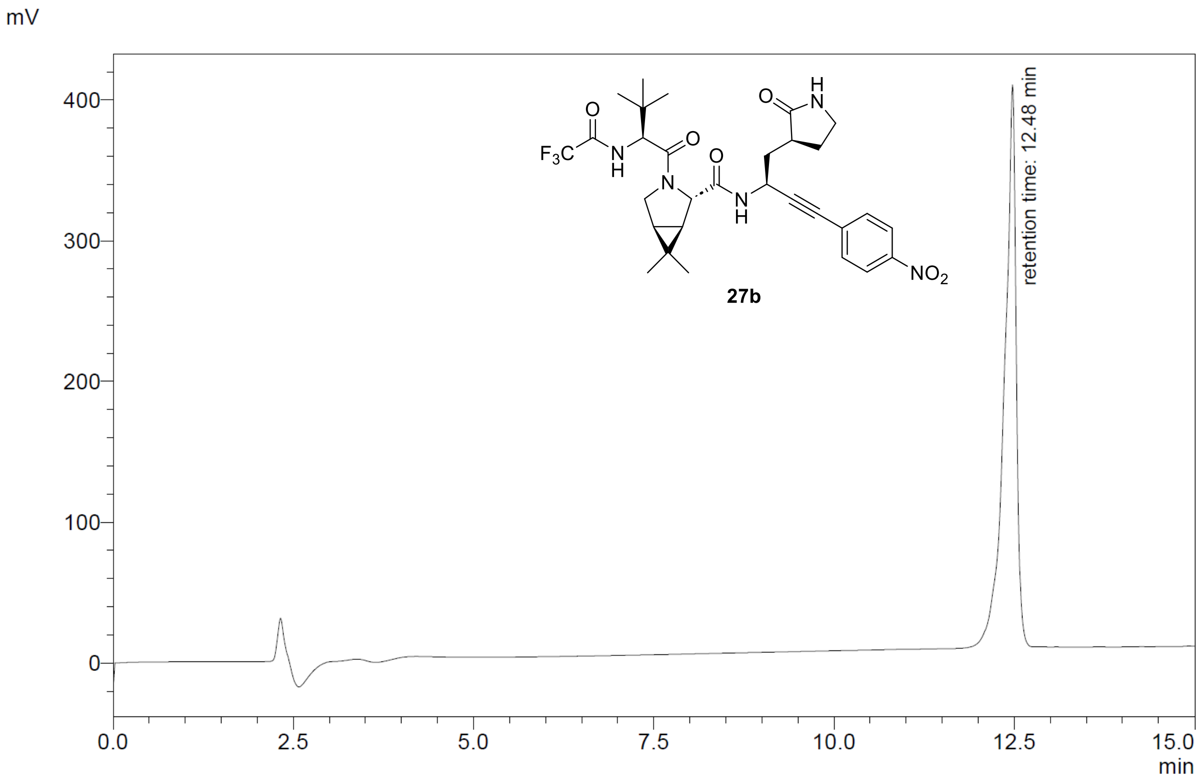
mV
400
300
200
100
0
0.0
2.5
5.0
7.5
10.0
12.5
15.0
min
retention time: 12.48 min
27b

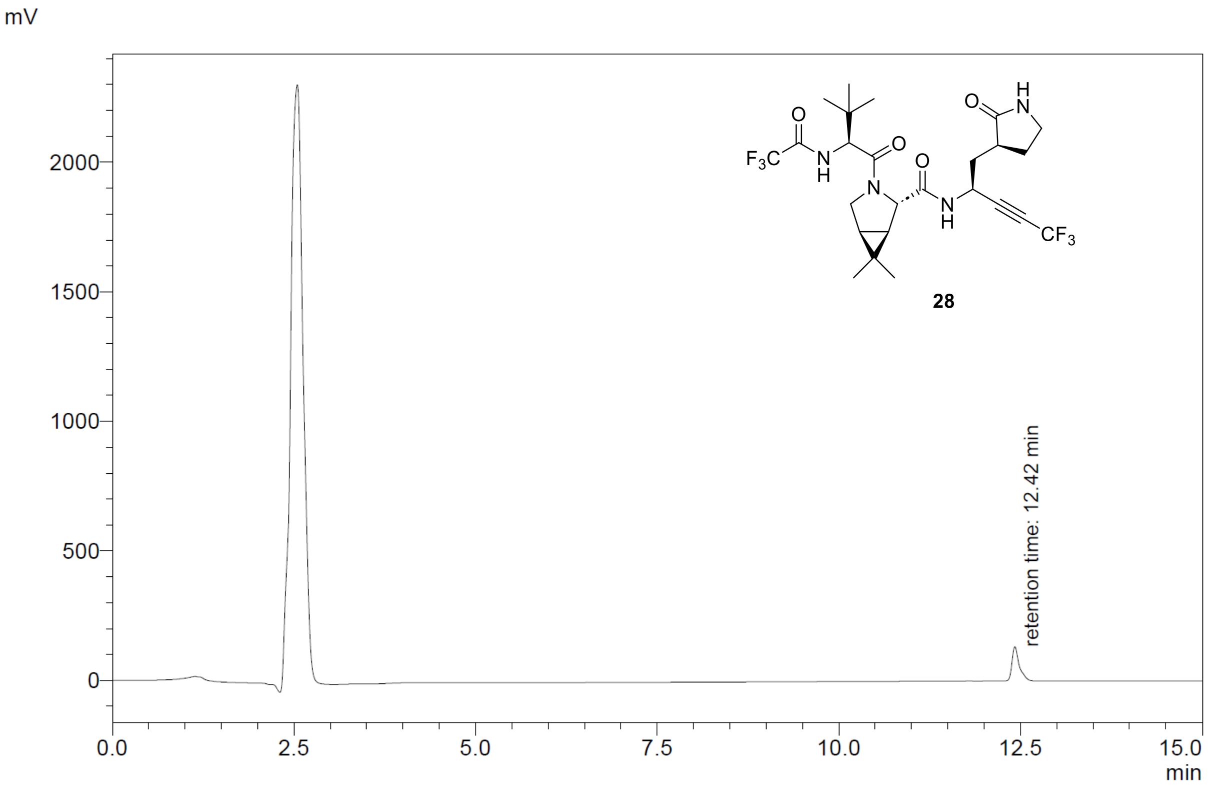
mV
2000
1500
1000
500
0
0.0
2.5
5.0
7.5
10.0
12.5
15.0
min
retention time: 12.42 min
28
